# Direct measurement of protein dipoles reveals the mechanism for like-charge adsorption on silica

**DOI:** 10.1038/s41467-026-76501-8

**Published:** 2026-09-15

**Authors:** Luis Lechaptois, Antoine Miche, Qingao Li, Souhir Boujday, Sierin Lim, Olivier Pluchery

**Affiliations:** 1 Institut des Nanosciences de Paris (INSP), Sorbonne Université, CNRS, UMR7588, Paris, France; 2 School of Chemistry, Chemical Engineering and Biotechnology, Nanyang Technological University, Singapore, Singapore; 3 Laboratoire de Réactivité de Surface (LRS), Sorbonne Université, CNRS, UMR7197, Paris, France

**Keywords:** Nanoscale biophysics, Applications of AFM, Biomaterials - proteins

## Abstract

Negatively charged particles are expected to adsorb with difficulty onto negatively charged surfaces. However, some proteins do. In this work, we use ferritin as a model protein particle to study its adsorption onto a negatively charged silica surface. Using Kelvin Probe Force Microscopy (KPFM), we provide direct measurement of the dipole moment of the protein ferritin on silica. The positive dipole value indicates that the negative end of the protein faces the surface. In this immobilization mechanism, our results unveil the pivotal role of sodium cations in the buffer solution. Changing the iron loading inside the ferritin, appears to add positive charges and the dipole is modulated without inducing structural changes. Comparison of quartz crystal microbalance results with KPFM reveals a strong correlation between rigidity and internal charge distribution. Our measurements provide insights on the relationship between dipole, protein adsorption, and the contribution of internal charge distribution.

## Abstract (web summary)

Negatively charged particles are expected to adsorb with difficulty onto negatively charged surfaces but some proteins do. In this study, the authors use ferritin as a model protein particle to study its adsorption onto a negatively charged silica surface and explain the electrostatics of this like-charge attraction.

## Introduction

Protein interactions with surfaces and their eventual adsorption are essential in many fundamental biological (*e.g*., transmembrane signaling, blood coagulation cascade)^1,2^, chemical (*e.g*., biocatalysis, biosensors, assays), as well as industrial processes (*e.g*., protein chromatography, filtration)^3–6^. It plays a key role in the biomedical field as it is the first phenomenon that occurrs upon introduction of biological implants in a human body^7^.

Despite considerable experimental and theoretical studies, a predictive understanding of protein adsorption is lacking^8,9^. Protein adsorption differs from that of small molecules and inorganic nanoparticles because proteins are softer and conformationally flexible, adapting their shape to the surface. Recent reviews consolidate the current knowledge on protein adsorption and present a unifying mechanism comprising three *stages*: (i) *bulk stage* where the migration is controlled by Brownian motion and convective forces; (ii) *boundary layer stage* where van der Waals forces and electrostatic forces, introduced by the proximal surface, control the protein approach; and (iii) *binding stage* where the physical contact between protein and surface results in bond formation with hydrogen and covalent as the dominant bonds^9,10^.

Local electrostatic forces are suggested to play a pivotal role within the boundary layer in *stage* (ii). Specifically, dipole moments of proteins give rise to dipole-dipole interactions between charged surfaces and proteins^11–13^. The interaction of proteins with surfaces is typically described by the Derjaguin–Landau–Verwey–Overbeek (DLVO) theory, which was initially developed to explain the kinetic stability of colloidal dispersions and later extended to encompass interactions between charged species in general^14,15^. In DLVO theory, repulsive electrostatic forces between like-charged entities in aqueous media prevent their close approach and interaction. When sufficiently strong, these repulsive forces confer colloidal stability by inhibiting aggregation or adsorption. In many cases, this description is inadequate because it does not take into account the fine-grained details related to local molecular (re)organization that gives rise to strong dipolar contributions^16^.

These local dipole-dipole interactions explain many recent counter-intuitive observations where like-charged particles attract. While the symmetric repulsion between like charges, regardless of the sign of the charge, is the foundational principle of electrostatics, reports of the adsorption of charged proteins on electrostatically like-charged surfaces (negative proteins on negative surfaces or positive proteins on positive surfaces) abound^17,18^. A recent study by Krishnan et al. provides a striking example of solution-mediated asymmetric attraction where negatively charged microparticles attract, but the positive ones repel^16^. The authors suggest that this unusual charge asymmetry arises because of the unique ways in which solvent orients at the interface, and generates a long-range electrosolvation force. Surfaces in contact with water develop a charged layer, called the “electrical double layer” (EDL), that includes polarized water molecules, counterions, solvated species. A recent study by Rorke et al. points the important role played by the hydrated sodium ions in the EDL of silica^19^. In our study, we investigate how hydrated sodium cations serve as *electrostatic bridges* and facilitate adsorption of negative species onto negatively charged surfaces.

These observations of like-charge attraction resulting from interface-mediated solvent reorganization naturally begs a question: Could surface charge asymmetry (and thus dipole moments) of particles themselves be altered by interfaces? This question is not only theoretical. Unlike solid inorganic microparticles or nanoparticles that typically assume spherical shapes, proteins – particularly those with multiple subunits and cage-like structures (e.g., ferritin, viral capsid) – have inherent structural symmetry but rarely a perfect sphere; instead they assume polyhedral shapes (e.g., cube, octahedron, dodecahedron, icosahedron)^20,21^. Because they fold in specific ways within their tertiary structure, their side chains are distributed in specific geometric arrangements presenting different surface properties (*e.g*., hydrophobicity and surface charge) along different facets of the molecule. Consequently, most folded proteins exhibit a well-defined dipole moment, reflecting the precise charge distribution^17^, which can be readily calculated using the Poisson-Boltzmann Equation (PBE) for free protein in solution^13,22^. But, in the vicinity of a macroscopic surface, proteins often undergo gross conformational and orientation remodeling. As a result, the dipole moment of proteins immobilized on surfaces is expected to change compared to its values in solution.

**Fig. 1:**
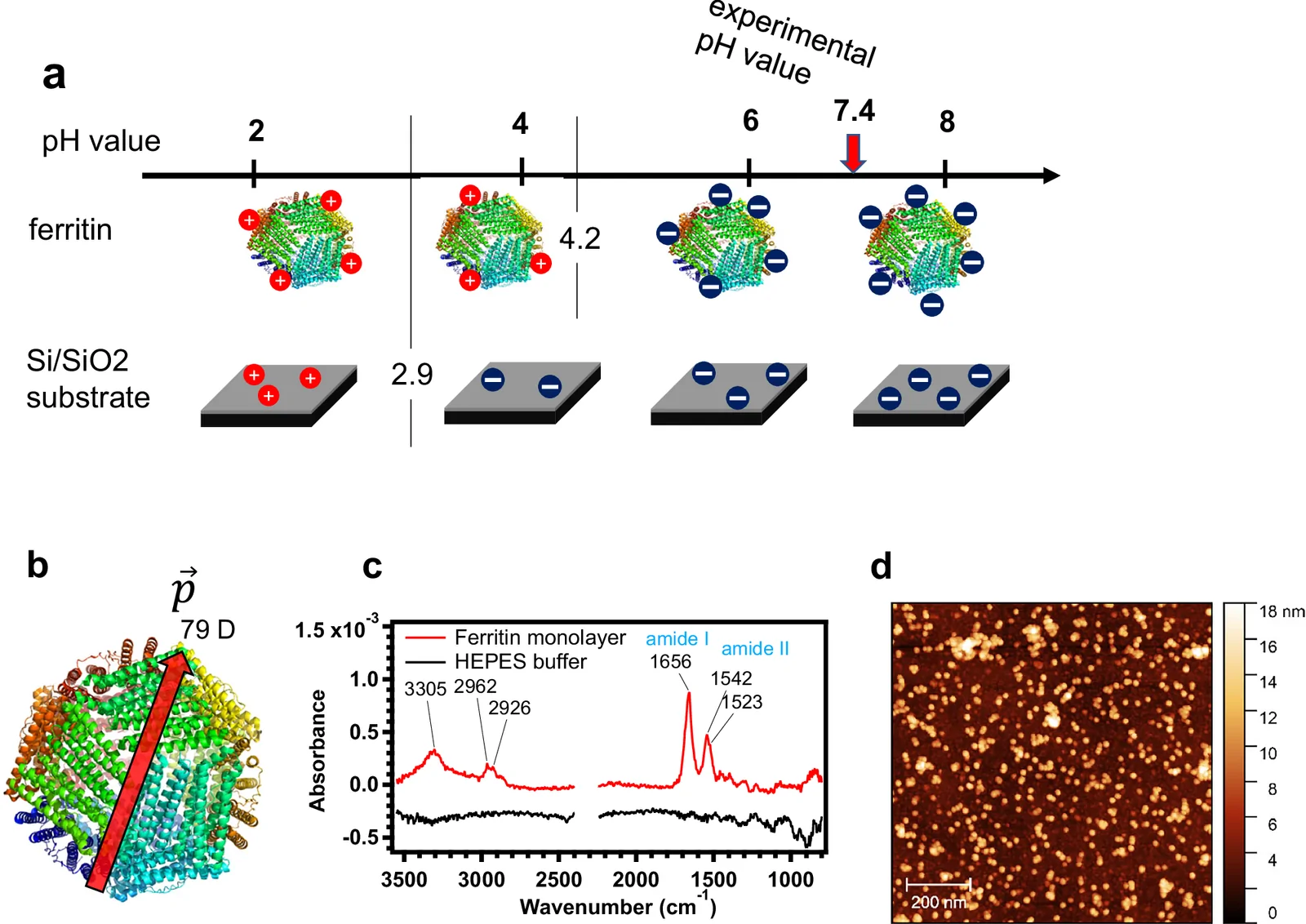
Evidence of the adsorption of ferritin nanocages on silica. **a** Representation as a function of pH of the electrical charges as a function of pH for the ferritin nanocage (isoelectric point at 4.2) and for the silica surface (point of zero charge at 2.9). **b** Representation of the ferritin nanocage, with the calculated electric dipole moment of the bulk phase. **c** Differential FTIR spectrum (3500 − 800 cm^−1^) of a (Fe4800)AfFtn-AA monolayer (red curve) and HEPES buffer (black curve) on a silicon wafer (FZ). The blank space at 2250–2400 cm^−1^ corresponds to the CO_2_ vibration peaks that have been erased on both curves for the sake of clarity. **d** AFM image of (Fe4800)AfFtn-AA drop-cast on silica obtained with a sharp AFM tip ScanAsyst-Air® (nominal tip radius is 2 nm) with scan area of 1 × 1 µm².

While the dipole moments of some proteins have been experimentally determined in solutions using electric dichroism and dielectric measurements^22–25^, they have never been measured on a surface. The direct measurement of the electric dipole would shed light on the adsorption process. In this study, we use ferritin as a model protein. Ferritin is an ubiquitous iron-storage protein found in all life forms and is known for its stability and structural properties^26^. The model ferritin is derived from the archaeon *Archaeoglobus fulgidus* (AfFtn; tetrahedron) and engineered to reduce iron release from the protein core (AfFtn-AA; octahedron)^27,28^. Ferritin is formed by the self-assembly of 24 protein subunits into a 12-nm nanocage (Fig. 1b). It is negatively charged at physiological pH of 7.4 and remains intact upon adsorption on surfaces and upon transfer in air. Iron is stored by converting iron(II) to iron(III) inside its core (see the *Method* section for details)^29,30^. The amount of iron atoms inside the core can be precisely controlled, with a maximal capacity of approximately 4800 iron atoms. Ferritin has been studied for various biomedical applications, such as a contrast agent for magnetic resonance imaging (MRI), bimodal fluorescence/MR imaging, and drug carrier^31–34^.

Here, we prepared five AfFtn-AA samples loaded with different amounts of iron ranging from 600 to 4800 atoms per ferritin that were drop-cast on silicon oxide surface. The adsorption was characterized in dry state using surface sensitive FTIR spectroscopy, atomic force microscopy (AFM) and Quartz Crystal Microbalance with Dissipation (QCM-D). The electrical charge distribution within the ferritin was investigated using Kelvin Probe Force Microscopy (KPFM) and the values of the dipole moment for the adsorbed samples were measured. AfFtn-AA is unlikely to adsorb at the physiological pH of 7.4 as depicted in Fig. 1a. However, we show that AfFtn-AA undergoes a stable adsorption on a negative silica surface in the presence of sodium cations. The combination of KPFM data with QCM-D provide correlation between the dipole and the rigidity of the iron core of ferritin.

## Results

In the first set of experiments, we deposited ferritin as monolayer on the thin silica layer grown on a silicon substrate. This initial surface was checked with AFM and exhibited an RMS roughness of 0.14 nm (Fig. S1). Solutions of AfFtn-AA nanocages in HEPES buffer (25 mM HEPES, 50 mM NaCl, pH 7.4) were drop-cast on substrates that were previously thoroughly cleaned, rinsed and dried (see *Methods* for details).

### Negatively charged ferritin forms a monolayer on negatively charged silica

Ferritin monolayer was obtained by drop-casting AfFtn-AA loaded with 4800 iron atoms (Fe4800) for 2 h. The solution was removed, rinsed with MiliQ water, and blow-dried with nitrogen. The deposition of the ferritin on the surface was confirmed by differential FTIR spectroscopy performed in transmission mode (see the *Methods* section). Figure 1c displays the FTIR spectrum obtained with a ferritin monolayer (red curve) while the spectrum with only the HEPES buffer (black curve) served as a control. It proves that in the absence of ferritin, no other chemical species remain after the rinsing step. In contrast, the ferritin monolayer spectrum shows peaks associated with the ferritin structural unit^35^. The 1656 cm^-1^ peak is assigned to the amide I band and those at 1523 and 1542 cm^-1^ to the amide II. These peaks are the footprint of protein amino acids. The other bands at 2926-2962 cm^-1^ and 3305 cm^-1^ correspond to CH_2_ and −OH binding vibrations, respectively, of ferritin. The surface topography image of (Fe4800)AfFtn-AA deposited on silica surface was recorded and shown in Fig. 1d. From the displayed image, it is possible to distinguish and count individual proteins on the surface.

FTIR and AFM results confirm the deposition of AfFtn-AA. The observation is counter-intuitive since the silica surface and the protein are both negatively charged at pH 7.4 as depicted in Fig. 1a. Silica is terminated with silanolates (SiO^−^)^36^ since it exhibits a point of zero charge around 2.9^37,38^ and the isoelectric point for ferritin is at 4.2 (*zeta* potential was −18 ± 4 mV)^39^. PyMOL electrostatic calculations also confirms that the external surface of the ferritin exhibits a dominant negative potential (see Fig. S9 in Supplementary Information).

### Single ferritin shows similar deposition process as the monolayer on silica

We questioned whether the like-charge deposition of the ferritin forming a monolayer was also observed at single-particle level. Hence, we measured the charge distribution of a single ferritin at varied iron loading in AfFtn-AA samples. Ferritin solutions were drop-casted for 1 min to allow for sparse deposition, followed by a rinse with MiliQ water.

**Fig. 2:**
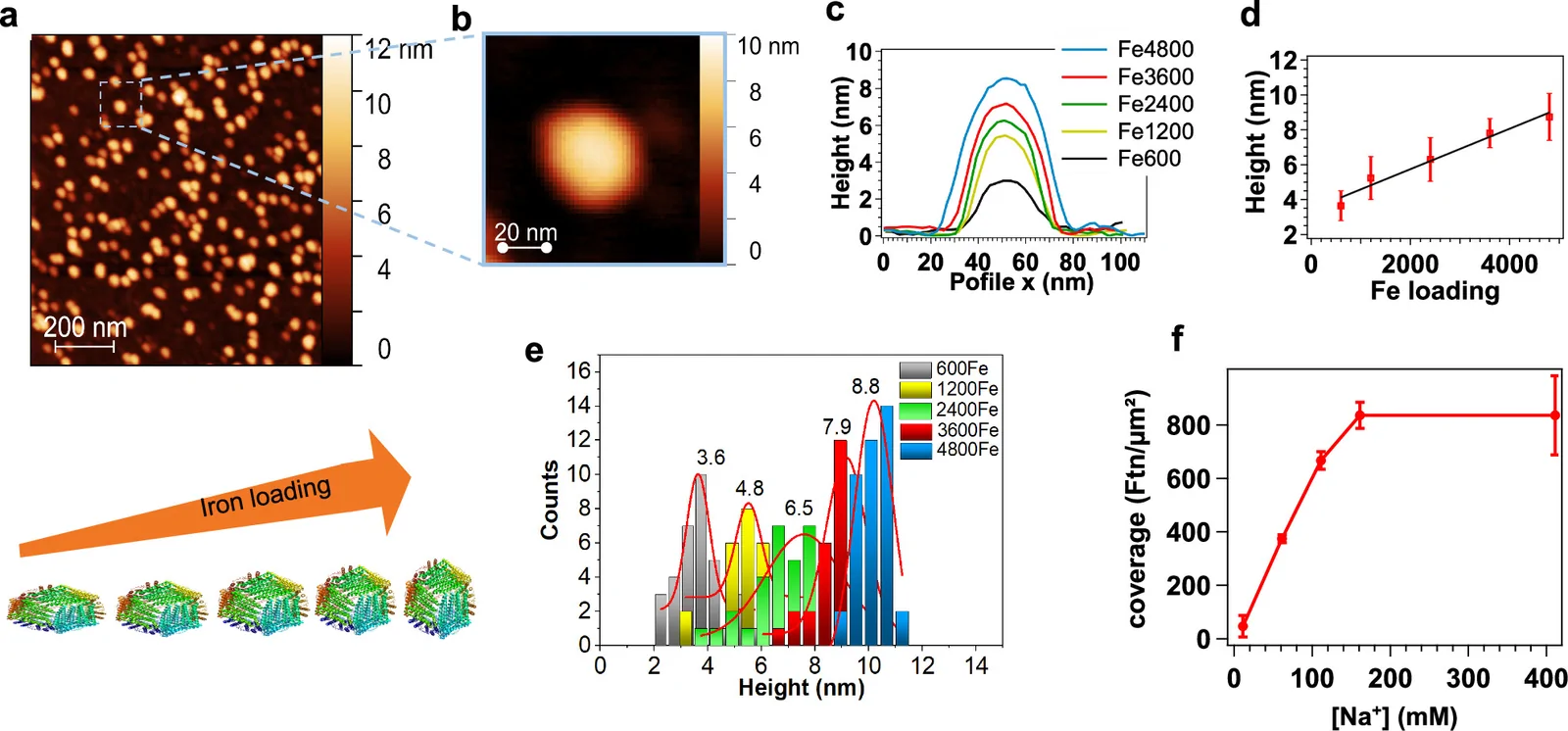
Topography results from AFM measurements of ferritin (AfFtn-AA) on a silica surface. **a** Topography image (1 × 1 µm²) of ferritin nanocages Fe4800 immobilized on silica recorded with the KPFM tip SCM-Pit-v2® (radius of curvature 25 nm). **b** Zoom in on a single ferritin particle. **c** Height profiles over ferritin particles as a function of their iron loading. **d** Plot of the average measured heights of ferritin at different loadings on silica with a linear fit of the data. **e** Height distributions of ferritin particles with different loadings on silica and the corresponding fits with Gaussian distributions. Average height from each distribution is: (Fe4800) *d* = 8.8 ± 0.6 nm; (Fe3600) *d* = 7.9 ± 0.6 nm; (Fe2400) *d* = 6.5 ± 1.2 nm; (Fe1200) *d* = 4.8 ± 0.4 nm; Fe600 *d* = 3.6 ± 0.4. **f** Surface coverage of AfFtn-AA drop-cast on silica surface for 1 min, as a function of Na^+^ concentration. The surface coverage was measured with AFM.

Figure 2a shows an AFM image of (Fe4800)AfFtn-AA on a 1 × 1 µm² area. Individual nanostructures (yellow dots) are clearly distinguishable (Fig. 2b). Topographic images of the other samples with different loadings, show similar observations (see Fig. S3). The sizes of the nanostructures measured from the profiles show an average height of ${{\boldsymbol{d}}}$ = 8.8 ± 0.6 nm, which agrees with the size of the ferritin measured using TEM ( ~ 12 nm). The evaluated height distributions of the five samples are displayed in Fig. 2c–e. The height of the adsorbed ferritin increases with the iron content from ${{\boldsymbol{d}}}$ = 3.6 ± 0.4 nm (Fe600) to ${{\boldsymbol{d}}}$ = 8.8 ± 0.6 nm (Fe4800) showing that the ferritin expands when its iron core is progressively loaded. Similar behavior has been observed for AfFtn-AA immobilized on gold^40^ and graphene surfaces^41^. For all loadings, the AfFtn-AA lateral width remains at 32 ± 1.5 nm when its height changes. A decrease of the lateral width of 2.2 nm is expected but not detected due to the convolution with the KPFM tip (SCM-Pit-v2®, radius of curvature of 25 nm (SI, Section 4). Recording with a sharper tip (ScanAsyst-air®, radius of curvature of 2 nm, Figure S6) shows lateral widths of ~19 nm for (Fe2400)AfFtn-AA which is ~2 nm greater than (Fe4800)AfFtn-AA as expected. The data confirm the deformation of AfFtn-AA at different loadings upon adsorption on the surface and agree with previous observation^40^. Figure 2d shows that the height on a silica surface varies linearly with the amount of iron atoms in its core. Unlike other studies using horse spleen ferritin on graphite^42^, these AFM results show that AfFtn-AA does not denature upon adsorption on silica. It only exhibits a mechanical deformation, where the vertical compression is countered by the rigidity of the iron core.

### Unraveling the role played by the sodium cation in the immobilization of ferritins

Marchioro et al. reports that the solvated Na^+^ are located in the EDL^19^. We hypothesize that these positive charges constitute an *electrostatic bridge* that immobilizes the negatively charged ferritins. Sodium cations is a component of the buffer solution in the AfFtn-AA preparation during the drop-casting process. Figure 2e shows that the surface concentration of ferritin increases as the sodium concentration increases (SI, Section 7; Fig. S9 for the raw AFM images). This is confirmed by QCM experiments performed in stop-flow configuration. The frequency shift is $\Delta f=-2.5{{\rm{Hz}}}$ in the absence of Na^+^ and increases to −165 Hz in the presence of NaCl (Fig. S13). KPFM experiments show that when the buffer solution is drop-cast on silica, positively charged species are present on the surface until a rinsing step is carried out. We show that when the buffer solution containing 50 mM NaCl was in contact with silica for 2 h, then blown away, the KPFM measured a global potential drop of 240 mV. This is indicative of positive charges on the surface (SI, Section 8). If a rinse with MiliQ water was carried out, the surface potential barely changed of +20 mV (Figs. S10 and S11). These experiments confirm that the sodium cations are essential for the immobilization of the AfFtn-AA on silica.

### Evidence of the outward dipole moment of the adsorbed single ferritins

**Fig. 3:**
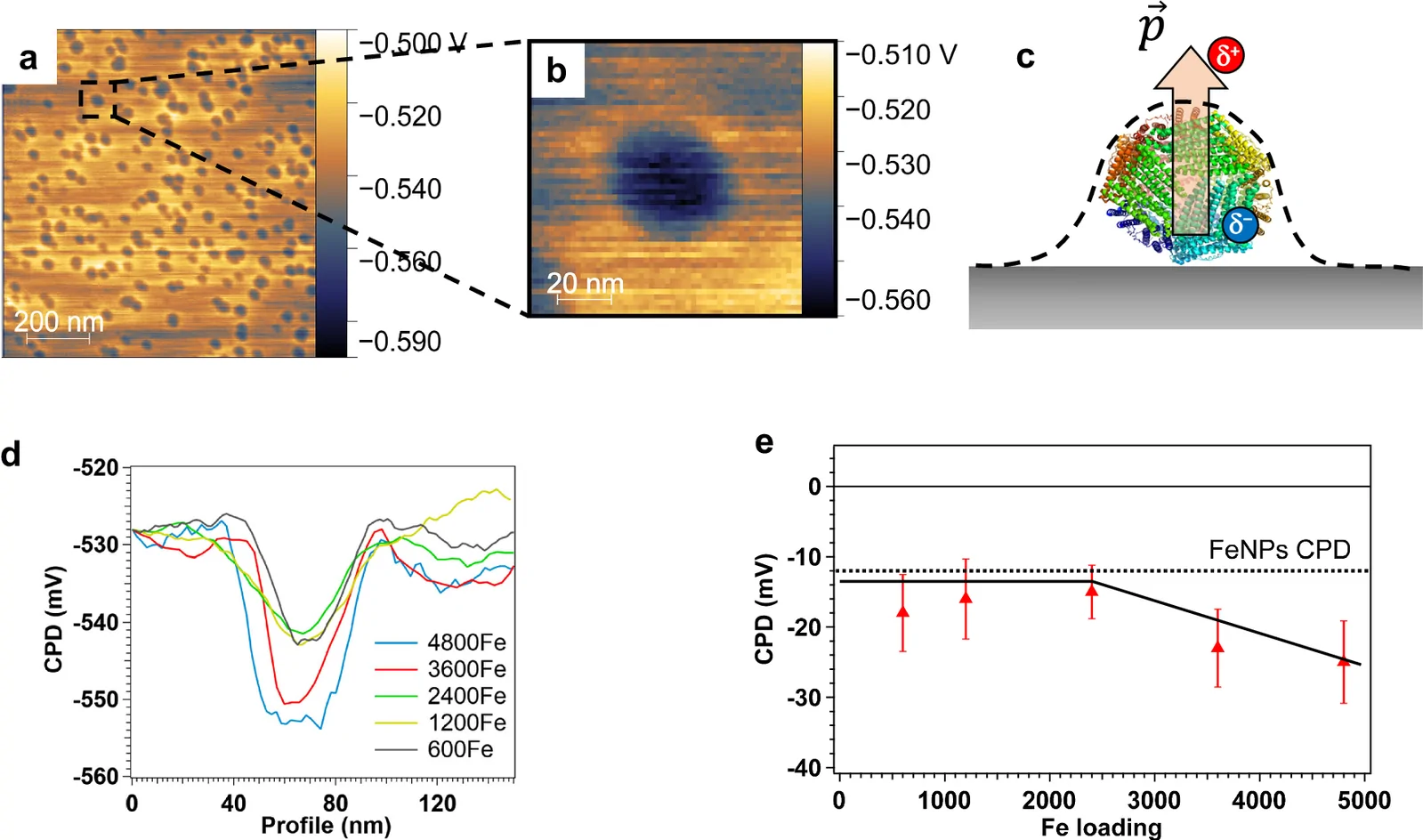
CPD results from KPFM measurements of ferritin on silica of exactly the same area as in Fig. 2. **a** CPD image of single ferritin nanoparticles (Fe4800) on silica. **b** Zoom-in on one ferritin nanoparticle. **c** Schematic of AfFtn-AA adsorption configuration along with the measured dipole. **d** CPD profiles taken on one ferritin at different loadings. Profiles were shifted and superposed for the sake of comparison. **e** Average measured CPD of single ferritins with different iron content. CPD of ferritin nanocages is measured compared to the silica one (i.e., CPD = CPD (ferritin) – CPD (SiO_2_)). Black dotted line represents the average CPD of single FeNPs. CPD values of ferritin with error bar are: Fe600: CPD = −18 ± 5.5 mV; Fe1200: CPD = − 16 ± 5.7 mV; Fe2400: CPD = −15 ± 3.8 mV; Fe3600: CPD = −23 ± 5.5 mV; Fe4800: −25 ± 5.9 mV.

To elucidate the reason of the deposition of negatively charged ferritin on silica, we measured the electrical properties and charge distribution at the interface of ferritin and silica using KPFM. Topography and contact potential difference (CPD) images of the ferritin samples loaded with different iron contents were recorded simultaneously in a two-pass process (see Fig. 2a, b, Fig. 3a, b and the *Methods* section). During the second pass, the tip scanned over the adsorbed ferritins, with a *lift scan height* (LSH) of 15 nm. In Fig. 3a, the relative CPD image shows the (Fe4800)AfFtn-AA samplesas in the topographic image of Fig. 2a. In Fig. 3b the CPD of a single ferritin (dark blue colour) is clearly distinguishable from the silica surface (yellow colour) and is shown to be negative, with a − 25 mV difference. The recorded relative CPD images for other loadings (Fig. S4) show a fainter contrast as the iron content decreases. At Fe600, the CPD of the nanocages (Fig. S4a) is below −10 mV and is barely distinguishable, compared to the background noise. These systematic negative values of the CPD are indicative of a specific charge distribution induced by the ferritins, where the negative charges accumulate closer to the surface and the positive ones further away as illustrated in Fig. 3c. This *outward dipole* moment is an unexpected result and will be discussed in detail.

Figure 3d shows the overlap of the CPD profiles over isolated AfFtn-AA for different loadings. The CPD values tend to decrease when the iron loading is higher than Fe2400. It is confirmed in Fig. 3e, where average CPD values (on 40 different ferritins) computed from the five samples are displayed. AfFtn-AA loaded with Fe600, Fe1200 and Fe2400 show similar CPD values (−18 mV, −16 mV and −15 mV respectively), and lower ones for AfFtn-AA at Fe3600 (−23 mV) and Fe4800 (−25 mV). This result demonstrates the influence of the iron oxide core on the CPD of AfFtn-AA nanocages. When the iron loading increases, the CPD decreases but not in a linear way: a threshold at Fe2400 is detected.

As the CPD depends on the nature of the nanostructure, we compared these values with the CPD of pure iron nanoparticles (FeNPs). Silica surfaces with iron oxide (II, III) nanoparticles (FeNPs) of similar sizes (15 nm) were prepared and analyzed by KPFM as well. The recorded images (Fig. S7a, b) show clearly the FeNPs both in topography and in CPD. The average measured CPD of FeNPs ( − 12 mV) is reported in Fig. 3e (black dotted line) and shows that the CPD change is less pronounced than for AfFtn-AA. Therefore, the CPD measured for ferritin is not merely due to the iron content, and the major contribution comes from the protein shell.

Our measurements show that KPFM provides information on the charge distribution of individual proteins. However, these values cannot be used for extracting a quantitative information because of the limited lateral resolution of KPFM^43^. The resolution for CPD was limited by the size of the tip (25 nm) and the LSH (15 nm), and was estimated at ~30 nm. Since ferritin diameter is 12 nm, the method only provides qualitative information for individual ferritins. Nevertheless, the consistent negative values of CPD are indicative of a charge distribution depicted in Fig. 3c that we will address in detail in the next section.

### Measuring the values of dipole moment of ferritin on silica

**Fig. 4:**
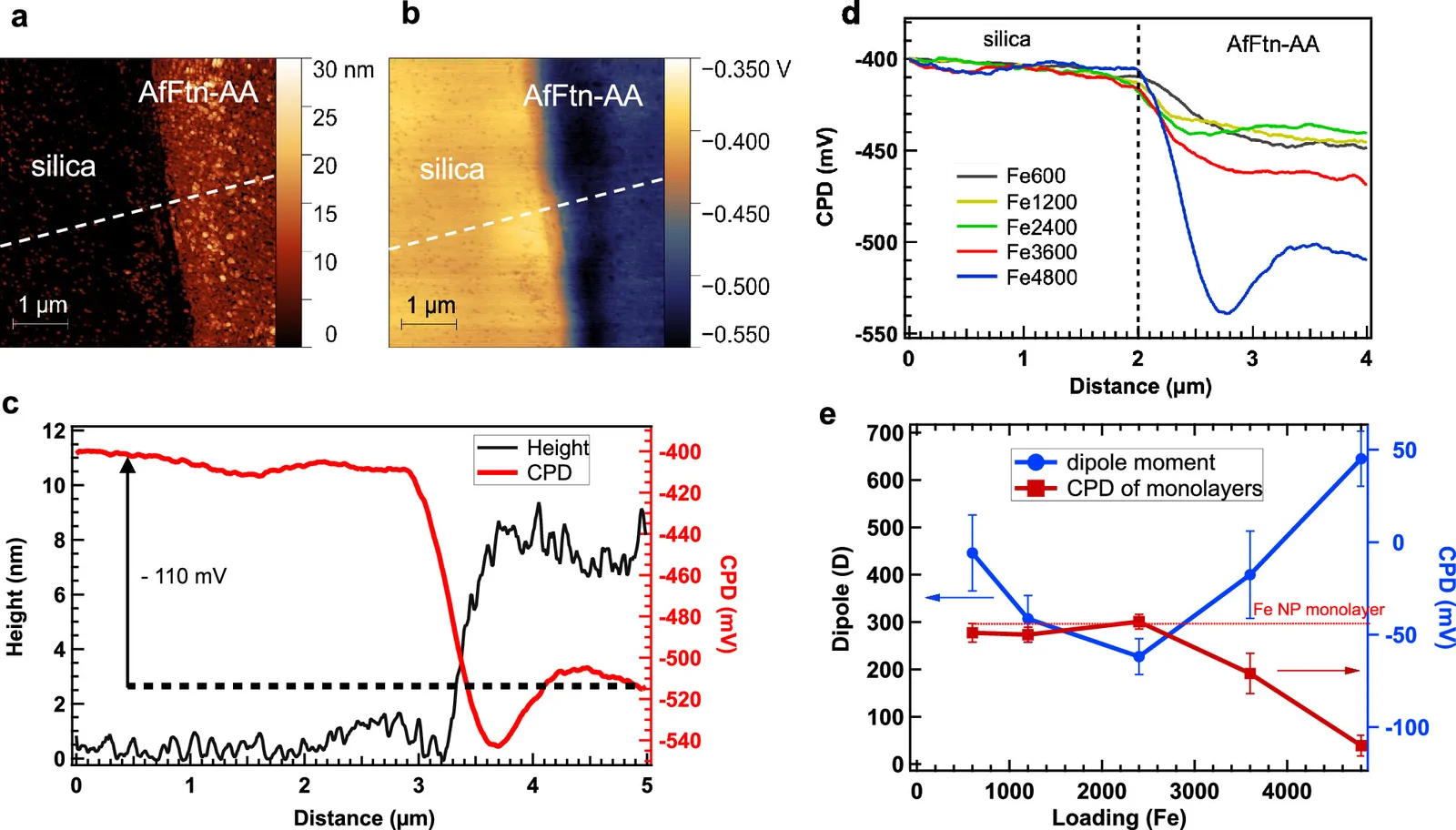
CPD results from the KPFM measurements of ferritin monolayers on silica. **a** Topography and (**b**) CPD images of a ferritin Fe4800 monolayer on silica. **c** CPD (red) and height (black) profiles along the white dashed lines. **d** CPD profiles taken across the silica/ferritin monolayer at different loadings. **e** Average CPD values of ferritin monolayer at different loadings, with error bars. The red dotted line represents the average CPD value of a FeNPs monolayer. The dipole moment of the ferritin is also displayed.

To obtain quantitative values of the dipole moment of adsorbed ferritins and to overcome the limited spatial resolution of the KPFM, we analyzed AfFtn-AA monolayers and compared it with FeNPs monolayer. Figure 4a shows the topography of the (Fe4800)AfFtn-AA monolayer sample recorded at the edge of the AfFtn-AA area. The height of the monolayer is measured at around 8 nm from the height profile (Fig. 4c, black line), which corresponds to the sizes measured on the single (Fe4800)AfFtn-AA sample. On the relative CPD image in Fig. 4b, we can clearly distinguish the AfFtn-AA monolayer (blue) from the silica (yellow), with a CPD drop of –110 mV (Fig. 4c, red line). Topography (Fig. S8a, e) and CPD (Fig. S8f, j) images of AfFtn-AA monolayer at other loadings and FeNPs monolayer (Fig. S7d, e) show similar edges between silica and AfFtn-AA areas. CPD profiles from each AfFtn-AA monolayer samples are displayed and overlapped in Fig. 4d. The average CPD values for the AfFtn-AA monolayers were calculated from the KPFM images and are reported in Fig. 4e. The three ferritin monolayers with the lower iron content show similar CPD values of –49 mV, −50 mV, and −43 mV for Fe600, Fe1200, and Fe2400, respectively. Beyond Fe2400, we see a net amplification of the CPD values with −71 mV for Fe3600 and −110 mV for Fe4800. In the case of FeNPs monolayer, however, the CPD remains at −44 mV as shown in Fig. 4e. The results show that for the FeNPs monolayer, the CPD is weaker than all AfFtn-AA monolayers.

These values are essential for understanding the local charge distribution induced by the adsorption of ferritin. The CPD contrast measured with a KPFM is made of three contributions, that add up: the work function (WF) of the substrate, the presence of electric charges and the presence of electric dipoles^44,45^. In Fig. S2, we show how KPFM is sensitive to the WF of the substrate materials, with CPD values evolving from −1100 mV, −430 mV and +0.030 mV when scanning through alumina, silica and gold, respectively. The CPD increase presented in Fig. 3 and in Fig. 4 when increasing the iron loading is not due to the increasing influence of the WF of iron. If it were the case, the CPD measured on the FeNPs of 15 nm should have been the most pronounced. Our results show the contrary. The CPD change is not due to permanent local charges, because they would have been compensated by counterions when the ferritin was drop-cast on the substrate or rinsed away as shown in section 8 of the SI. Therefore, these negative CPD values are due to a permanent dipole moment as discussed herein^46–48^. Our observations suggest that the CPD values are comparable to FeNP up to Fe2400 loading where a consistent gradual change was observed. This unique observation invites exploration on the effect of iron loading on the CPD which we will discuss in subsequent sections.

Using the Helmholtz formula, we calculated the value of the dipole moment of the ferritin from the CPD values^46^. The Helmholtz formula states that the electrostatic energy change $\Delta \varphi$ at a surface, induced by a layer of dipole moment of value p is given by $\Delta \varphi=-p/{\varepsilon }_{0}A$, where A is the average area occupied by one protein. A is simply linked to the surface density σ by A=1/σ, which is evaluated from the AFM images. The surface density for all iron loadings are found to be between 290 and 500 ferritin particles/µm² (Table 1 and Fig. S12).

**Table 1:** Adsorption of ferritins with various iron loadings on silica surfaces

Ferritin	Fe600	Fe1200	Fe2400	Fe3600	Fe4800	apo-AfFtn in solution
${{\boldsymbol{\sigma }}}$ (Ftn/µm²)	290 ± 25	430 ± 35	500 ± 40	470 ± 35	450 ± 35	-
CPD (mV)	−49 ± 5	−50 ± 4	−43 ± 4	−71 ± 11	−110 ± 6	**-**
${{\boldsymbol{p}}}$ (debye)	450 ± 80	310 ± 49	230 ± 38	400 ± 92	650 ± 58	79

Values of the surface coverage σ, measured with AFM in Ftn/µm². The CPD is directly obtained from the KPFM and is used to compute the net dipole moment *p* with the Helmholtz formula. The data are calculated for the AfFtn-AA monolayers at different loadings. The net dipole moment of apo-AfFtn-AA is known in solution only (last column)^49^.

The results in Table 1 show that AfFtn-AA dipole undergoes a slight evolution which is not monotonic with the iron content. The Fe600 AfFtn-AA exhibits a value of 450 D and the Fe4800 a value of 650 D. Interestingly, the dipole moment goes through a minimum value of 230 D for (Fe2400). These values are notably larger than the ferritin in solution whose dipole moment was calculated to be 79 D^49^. Our dipole values scale with those calculated for other proteins, such as hydrophobin (7-15 kDa), that exhibits a dipole moment of 104 D^50^, or BSA with a dipole moment of 650 D^51^. However, to our knowledge, this is the first time the dipole moment of an adsorbed protein, particularly multi-subunit protein, is measured on a surface. Our results show that adsorption leads to a strong increase of this dipole, an effect which has been predicted by a theoretical study for cytochrome c with an increase from 279 D (free proteins) to 661 D (adsorption on a negatively charged COO^−^ SAM)^52^. This sharp increase reveals a profound reorganization of the charges at the interface which includes two contributions: the morphological change of the ferritin, evidenced by our AFM measures and a modification of the boundary layer as predicted by Kubiak-Ossowska for water^51^, and evidenced by our results of the role of the Na^+^ cations.

Moreover, Fig. 4e also points to the striking change of slope in the evolution of the CPD as a function of the iron loading. As discussed above, CPD measures the local charge organization that eventually leads to the formation of a permanent dipole. For Fe600, Fe1200, Fe2400, the ferritin particles exhibit almost no internal charge re-arrangement, even if the morphology shows a regular height increase (Fig. 2e). For Fe3600 and Fe4800, the permanent dipole tends to grow. The dipole moment evolution undergoes a clear threshold at Fe2400. This points to changes to the internal structures involving the interactions between amino acid shell and the iron core.

### Changes in electrical dipole correlate to the changes in iron core structure

The abrupt change in slope of the CPD related to the variation of iron loading may indicate changes in the internal structure of the iron core in ferritin and reflects also the progressive uptake of positive charges from iron(II) and iron(III). We hypothesized that it would result in a change in viscoelastic properties of ferritin. Hence, we performed coupled QCM-D and LSPR (localized surface plasmon resonance) measurement on ferritin monolayers adsorbed on nanostructured quartz^53^. The experiments were first carried out in a dynamic regime using a peristaltic pump. In these conditions, flowing the ferritin solutions onto the quartz did not lead to their adsorption because the ferritin barely entered the boundary layer and could not adsorb on the negatively charged surface. We therefore modified the surface by grafting aminopropyltriethoxysilane (APTES) to generate positively charged surfaces with amino groups which favor ferritin adsorption despite the flow^54^.

**Fig. 5:**
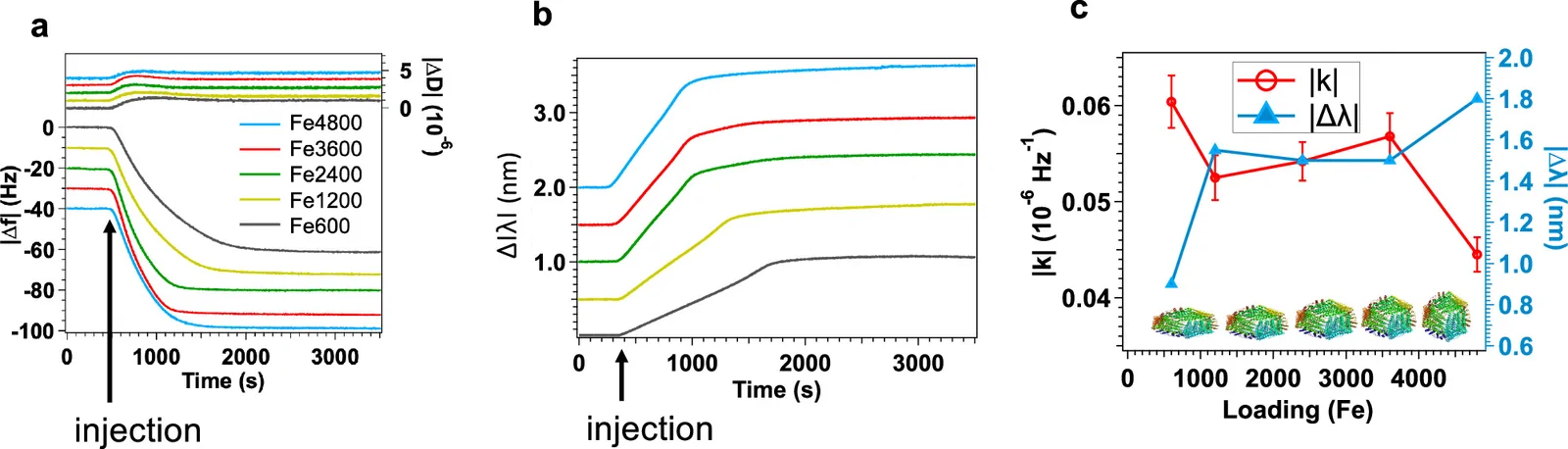
QCM-D and LSPR data related to ferritin adsorption on silica surfaces. **a** Changes of frequency Δ*f* and the dissipation Δ*D* with the increase of iron content (the curves were offset to facilitate visual reading). **b** Shift in the LSPR wavelength Δ*λ*, measured simultaneously to the QCM data (the curves were also offset). **c** The slopes *k* of the Δ*D vs*. Δ*f* plots and the final LSPR wavelength as function of the iron content in the ferritins.

The QCM-D data in Fig. 5a shows the shift in frequency *f* upon ferritin adsorption and the related dissipation *D* increase. The curves show the initial stabilization in buffer, then, upon injection of ferritin solutions, the frequency decreases rapidly as a result of a mass uptake at the quartz surface while the dissipation slightly increases, then, both *f* and *D* reach a plateau and stabilize. In all cases, the final frequency shift is approx. 60 Hz. Even if this shift is similar for all loadings, the plot of the dissipation variation as a function of the frequency shift allows calculating the slope *k* of the linear portion. This provides a deeper understanding of the viscoelastic properties of the layers. The *D* − *f* plots for the different loadings are shown in Fig. S14 and the slopes *k* are represented in Fig. 5c. Clear differences can be seen when comparing the lowest iron content to the highest one. Fe600 leads to a *k* value 35 % higher than that measured for Fe4800, denoting considerably higher viscoelasticity of the formed layer. Interestingly, for the three intermediate iron loadings, no significant differences are observed and the *k* values remain within a 5 % interval. These values confirm that (Fe600)AfFtn-AA forms a soft protein layer, whereas (Fe4800)AfFtn-AA forms a considerably more rigid one.

Figure 5b displays the results obtained for the same experiments by LSPR. As for QCM data, there is the initial stabilization, then an increase of the LSPR wavelength position upon injection of ferritin solutions finalized by a plateau at the equilibrium. The final wavelength shifts are plotted as a function of the iron content in Fig. 5c. Unlike the frequency shifts measured by QCM-D, the LSPR responses behaved differently: the highest iron loading led to a shift twice that of the lowest one, while, for the three intermediate loadings the variation remains within an interval lower than 5 %. Since LSPR is a refractometric technique, the wavelength shift is induced by the change in the refractive index on the surface induced by protein adsorption. Therefore, the water molecules possibly trapped within the protein layer, especially when this layer is not rigid, have no impact on the response. In contrast, QCM-D, is a piezoelectric technique and the measured frequency shift is the result of mass uptake at the surface which includes hydration. As a result, the acoustic masses are considerably higher than the optical masses, as can be seen in Table S5.

Finally, both QCM-D and LSPR show that the nature of the ferritin layer is dependent of the iron content. At low iron content, the formed layer is viscoelastic and highly hydrated while at high iron content the layer is rigid and compact, with a level of hydration twice lower. The data in Fig. 5c suggest that the 2400 loading is the inflection point of this profound change in the ferritin layers properties where the internal structural change occurs.

## Discussion

Protein adsorption has been largely discussed using the kinetic and thermodynamic models developed for molecular adsorption^9,10^. However, due to their specific viscoelastic properties, proteins exhibit distinctive behaviors. In particular, the adsorption of a globally negative protein on a negatively charged surface is surprising. Yet, adsorption of proteins with net negative surface charge on negative surfaces have been reported with AFM^55^ and are typically explained by the positively-charged patches that are innately present on protein surfaces^17,50^. This explanation stems from simulations but was never measured before.

**Fig. 6:**
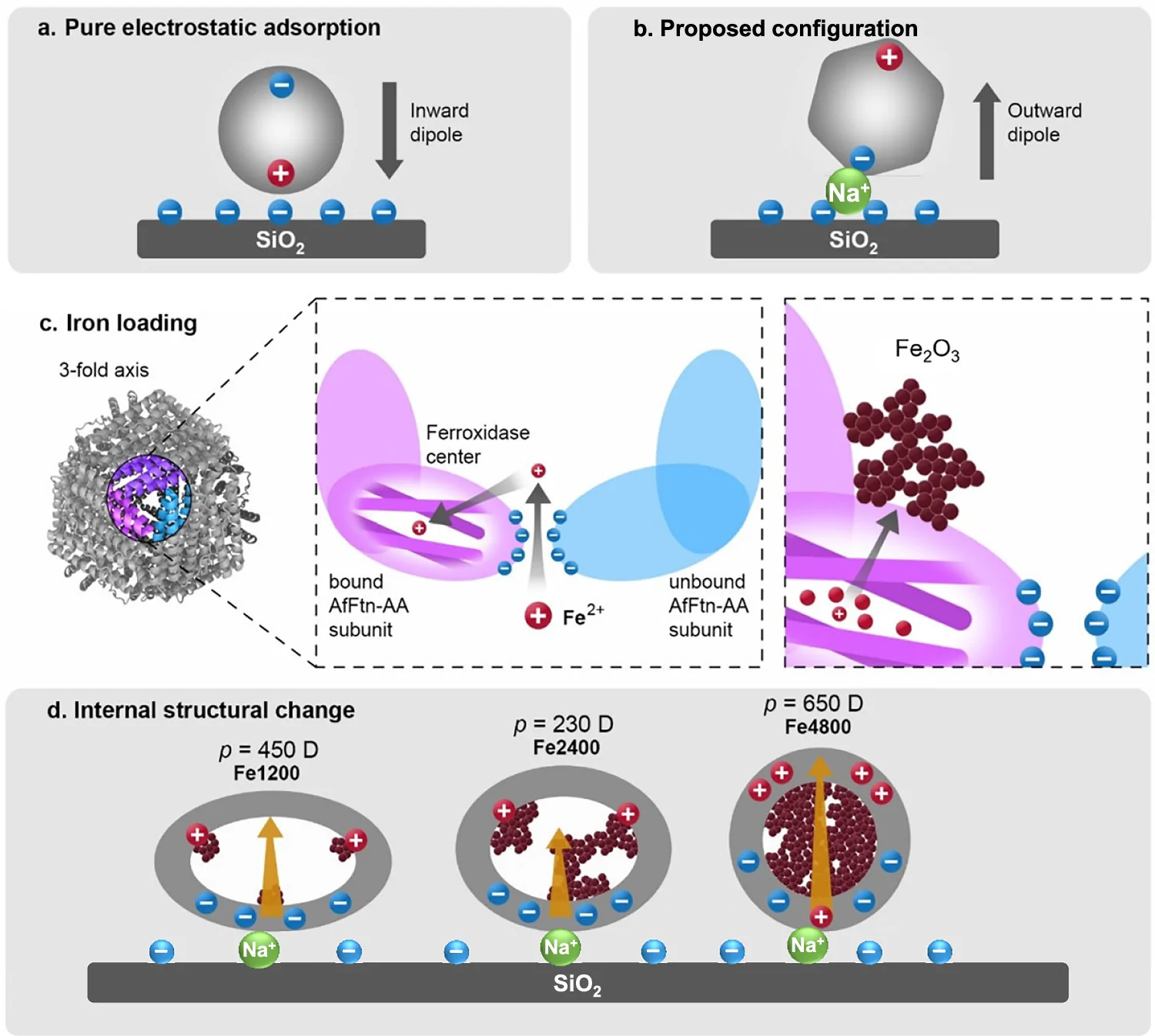
Scheme of the adsorption configuration of ferritin on silica. **a** Representation of a purely electrostatic interaction of a dipolar species adsorbed on a negatively charged surface where the positive end of the dipole binds to the surface. The dipole would be *inward* with a negative dipole moment. **b** The ferritin is measured to have an outward dipole with the negative charges being connected to the silanolate groups of the silica surface through sodium cations. **c** Schematic of the iron loading mechanism: iron atoms flow through the 3-fold axis to reach a ferroxidase center, where they are oxidized into Fe^3+^ and form the mineral core Fe_2_O_3_. This results in the uptake of local positive charges. **d** Once adsorbed on a silica surface, outward dipoles are created. The repartition of charges induced by the adsorption and by the uptake of positive iron atoms inside the ferritin, forms a dipole that tends to grow with the iron loading.

For all five iron loadings of ferritin, our results show that the protein adsorbs on silica surface and exhibits a strong outward dipole moment (positive dipole). A purely electrostatic adsorption would have led to the positive end of the dipole facing the negative silanolates of the surface as shown in Fig. 6a. However, our results prove that during the boundary layer stage of the adsorption, the solvated sodium cation acts as an *electrostatic bridge* and helps the anchoring of ferritins. This bridging cation is protected by the ferritin during the rinsing step, leading to the configuration depicted in Fig. 6b, where the protein exhibits a charge distribution with an excess of negative charges towards the surface. The dipole values are in the range of 300-600 D. These measured values for ferritin are in the same magnitude as the values calculated for BSA, hydrophobin, and cytochrome c^50–52^. The outward dipole observation can be explained by several factors.

The first factor is the presence of positive residues on the ferritin as calculated using PyMOL and shown in Fig. S15b, c. The positive residues Lys7 and Lys114 are situated at the C4 axis of the AfFtn-AA. Therefore, we can expect that these residues play a role in anchoring the protein on the surface. This is consistent with results for the adsorption of BSA on silica surfaces^11^. Kubiak-Ossowska et al. performed molecular dynamics simulations and highlighted the role played by the positive arginine residue of BSA^51^. In addition to BSA, this orientation mechanism has been predicted for cytochrome c^52^, and hydrophobin^50^. As the shape of the AfFtn-AA is octahedron, we expect that this particular adsorption mechanism, leads to ferritins precisely oriented on the silica surface with one of their eight sides facing the surface anchored at the C4 axis. The second factor that controls the adsorption of proteins is the hydrophobic interaction that dominates over the electrostatic contribution at high or low pH. We cannot rule out hydrophobic interactions. However, this is probably not the main element since silica surface is hydrophilic^56^. Finally, the last factor is the recently explained phenomena where like-charged colloids attract themselves, if they are both negative, but not if they are both positive^16^. In their study, Krishnan *et al*. stressed the crucial role played by water as solvent, since it forms a dipolar layer that eventually generate a long range force leading to negative silica particles attracting each other. In our case, a similar mechanism taking into account the sodium cations helps understanding the attraction and the immobilization of the negative ferritins on negative silica. Our results show a global increase of the dipole moment when positively charge iron (II) and iron (III) are progressively loaded into ferritin (Fig. 6d). Without changing any other adsorption factors, they prove that the dipole moment is essentially generated by the ferritin. These results do not contradict the DLVO theory, as described in the introduction—especially considering that the contribution of Na^+^ counterion supports the observation of like-charge attraction. Rather, they provide experimental data to the more detailed and refined understanding provided by molecular dynamics simulations^50,51^.

Finally, our results show a correlation between the dipole and the internal structure of ferritin. The AFM results reveal that the height of the nanocages scales linearly with the number of iron atoms in the core. The shape of the ferritin evolves from a disk at low loading (Fe600) to spherical structure at high loading (Fe4800). It was noted that at Fe2400 loading, the CPD started to change. The observation led us to investigate the change of ferritin internal structure at different loadings. The rigidity of the ferritin layer deduced from QCM-D and LSPR data exhibits a non-proportional evolution with loading. The protein layer is more viscoelastic at low loading in iron and more rigid at high loading with a stagnant phase at medium loadings (Fe1200, Fe2400, Fe3600). In our earlier modeling study, we suggested the inward growth of the iron core from the nucleation sites located at the internal surface of the protein nanocage^29^. During the iron loading process, iron(II) ions bind to the ferroxidase centers in the inner surface of the shell, are converted into iron(III) to form the mineral oxide core (Fig. 6c). The nucleation occurs at multiple sites in the core and grows into the lumen of the cavity forming crystals of iron oxide nanoparticles^57^. The formation of iron oxide nanoparticles explains the resulting change of the viscoelastic property of the ferritin. Significant changes are observed at Fe1200 and Fe3600. The midpoint of this process is Fe2400 and it correlates with the CPD result where the value abruptly changed. We speculate that the iron grows within the cavity from different nucleation sites, and eventually form contacts. For the low-loading ferritins ( ≤ Fe2400), the mineral core does not bind to all subunits that results in disrupted structures upon adsorption (Fig. 6d). On the other hand, for high-loading ferritins ( > Fe2400), all subunits are bound to the mineral core resulting in a higher resistance to surface-induced structure changes. There is a clear internal structural transition at about Fe2400. It is interesting to note that this transition point matches the change in charge distribution of the ferritin, as demonstrated by the minima at Fe2400 for evolution of the dipole moment. Since the dipole moment is $p={qd}$, we expect that p grows with the ferritin size. The observed minimal value for p probably corresponds to an intermediate state where charges are more diluted inside the ferritin which reduces q and therefore p. Due to the orientation of the protein on the surface, and since the bond is likely to be formed through the 4-fold axis (positive residues), the protein is arranged so the three other 4-fold axes point outwards as represented in Fig. 6d. The negatively charged residues may have interacted with the sodium ions in the buffer imposing additional dipole moment. Furthermore, the presence of both iron(II) and iron(III) in the core of AfFtn-AA provide mobile elements that contribute to the change of dipole in the core. With the increase of iron(III) loading, the positive charge tends to accumulate and explain the increase of the dipole until it reaches its higher value of 650 D. The minimal value of 230 D at Fe2400 should also be related with the results from Nijhuis *et al*. where they measure the charge transport through AfFtn-AA^40,41^. They report a transition in the conduction regime from a long-range tunneling to hoping mechanism at Fe2400.

The evolution of the outward dipole moment is certainly due to rearrangement of charges upon loading of iron in ferritin and is a unique phenomenon. Contrary to the other observed situation in the like-charge adsorption or self-assemblies where solvent rearrangement has been proposed as the major contributor, we observe here with ferritin, a double effect of *electrostatic bridging* mediated by the sodium cation and a *solute rearrangement* of the inner core that explains the buildup of stable and strong dipole moment on the negatively charged silica surface.

This study provides evidence of the immobilization of negatively charged proteins on a like-charged surface and we show that this behavior is mediated by the sodium cation. The local charge distributions have been measured for individual ferritins adsorbed on silica as well as for a full monolayer and allow for extraction of the dipole moment of the adsorbed protein after the final surface have been dried. The fully iron-loaded ferritin (Fe4800)AfFtn-AA exhibits an outward dipole of 650 D, where the negative charges are found to be closer to the silanolate negative surface whereas the positive ones are away from the surface. This counter-intuitive observation is explained by the intercalation of sodium cations between the surface and the ferritin. Moreover, the internal reorganization of the ferritin is also partly revealed and show a transition point at Fe2400 when the iron content grows from 600 to 4800 iron atoms.

This ability of assessing how charges reorganize for adsorbed ferritin opens new perspectives for better understanding the adsorption of proteins on solid surfaces through electrostatic bridging by cations. Understanding precise locations of the static electric charges as well as their transfer pathways will contribute to the development of design principles for biomolecular electronics.

## Methods

### Materials

Silicon wafers were used “as-received” in our experiments and the silica surfaces of this study corresponds to the 2 nm thick thermal oxide grown on the substrate. A careful cleaning was systematically carried out but without modifying the surface oxide. For KPFM measurement, the wafers were purchased from Siltronix (Czochralski grown CZ, <111 > , *n-*type, antimony dopant) with a dopant concentration of 5×10^18 ^cm^−3^ (0.01 Ω.cm) so that they are conductive. For FTIR experiments however, silicon wafers Si(100) were bought from ITME double-side polished, and with a high resistivity (20 Ω.cm, dopant concentration of 10^14 ^cm^−3^*, n-*type, doped with phosphorus) to make them transparent to infrared. Moreover, they were chosen grown with the float zone (FZ) method so that they have a very low amount of residual oxygen in the bulk of the crystal, and allow meaningful FTIR measurements in the 1000 − 1500 cm^−1^ region. MilliQ water (18.3 MΩ.cm) was used for solution preparation and rinses.

For buffer solution, HEPES (2-[4-(2-hydroxyethyl) piperazin-1-yl] ethane-1-sulfonic acid), sodium chloride (NaCl) were purchased from Sigma-Aldrich.

Iron oxide (II, III) nanoparticles and (3-Aminopropyl)triethoxysilane (APTES) were purchased from Sigma-Aldrich. Nanoparticles are dispersed in water and coated with maleic anhydrate-methyl vinyl ether copolymers.

### Protein nanocage production

The ferritin is derived from the hyperthermophilic archaeon *Archaeglobus fulgidus* (AfFtn). The wild-type AfFtn has four open pores that result in an increased rate of iron release from the core to the exterior over the time. To remedy this issue, we used a modified AfFtn with closed pores, referred to as AfFtn-AA (PDB ID: 3KX9), where two amino acids were replaced (K150A /K151A) on each subunit via site-directed mutagenesis. Production and purification of *A. fulgidus* ferritin-AA (AfFtn-AA) was performed as described elsewhere^58^. Briefly, the recombinant production was carried out in *Escherichia coli* BL21(DE3)C + RIL cells (Stratagene, Santa Clara, CA) as the host. The AfFtn-AA was purified using hydrophobic interaction chromatography and was stored in HEPES buffer (25 mM HEPES, 50 mM NaCl, pH 7.5) at 4 °C until further use.

### Iron loading of protein nanocage

Loading of 4800 molar ratio of iron(II) into AfFtn-AA results in different composition of positively charged iron(II) and iron(III) depending on the method^57^. Loading at room temperature with aerobic condition results in higher iron(II) to iron(III) ratio (60:40) while at high temperature (i.e., 65 °C) with anaerobic condition the ratio of iron(II) to iron(III) is relatively equal (55:45). In this study, iron has been loaded at room temperature with aerobic condition and we expect to have higher iron(II) composition compared to iron(III). Furthermore, multiple nucleation sites have been identified in AfFtn-AA. As more iron(II) is converted into iron(III) and nucleate, iron oxide particles form and the core fills up resulting in potentially more rigid structures that is more resistant to deformation^40^.

Iron (Fe) loading in AfFtn-AA was performed in aerobic conditions. Ferrous sulfate (FeSO_4_) solution in 0.1% hydrochloric acid (HCl) was freshly prepared and various amounts were added at intervals to a solution containing dimeric apo-AfFtn-AA (0.25 mg.ml^−1^ in HEPES buffer, pH 7.5). Samples were incubated overnight at 4 °C post loading. Fast Protein Liquid Chromatography (FPLC) with a HiPrep^TM^ 26/10 Desalting column was used to remove the unbound iron by buffer exchange with appropriate buffer solution. Iron quantification was performed using inductively coupled plasma mass spectroscopy (ICP-MS) and protein quantification using the Bradford assay (BioRad). In our experiments, amounts of FeSO_4_ were adjusted in order to obtain loadings of 600, 1200, 2400, 3600, and 4800 iron atoms per ferritin.

### Protein nanocage deposition on silica

Silicon wafers were cut into 1 × 1 cm² (3 × 1 cm² for FTIR) pieces with a diamond tip, cleaned with acetone and ethanol under sonication and dried under nitrogen flow. Prior to deposition, protein solutions were filtered with a 0.22 µm filter to remove dust particle and precipitated unbound iron. One drop of the solution (0.25 mg.ml^−1^ in HEPES buffer, pH 7.5) was drop-cast onto each of the silicon substrates. Two deposition time were used: 1 min to obtain dispersed proteins and 2 h for producing monolayers. Finally, samples were thoroughly rinsed with MilliQ water and dried under a nitrogen flow.

### Kelvin probe force microscopy (KPFM)

KPFM is an advanced technique based on atomic force microscopy (AFM). When the tip scans over the sample surface, both topography and contact potential difference (CPD) are recorded. The CPD map corresponds to the local surface potential, which in turn, depends on three main contributions: the nature of the material, the local fixed charges and the dipoles present at the surface. KPFM has been mainly used to map the surface potential or the local work function (WF) of nanoparticles^59,60^, quantum dots^56,61^ or thin films^48,62^. on solid surfaces. Previous KPFM work on biomaterials have revealed the surface potential of proteins^63^, DNA^64^, or lipids^65^, but at a low resolution or for monolayers. Leung *et al*. showed topography and surface potential images of single avidin proteins (a 58 kDa tetrameric protein) and DNA, on silicon and mica surfaces^66,67^. However, these studies only discussed the image contrast without providing any quantitative information contained in the CPD values. Previously, our group reported on a quantitative approach for metallic nanoparticles^48,59,68,69^.

More precisely, KPFM measures the local variations of the surface potential by detecting the CPD between a sample and a conductive AFM tip. It produces a CPD map. When the tip is brought close to the surface, Fermi levels align as a result of charge transfer. The electric charges are reorganized between the tip and the sample and the CPD builds up (denoted ${V}_{{CPD}}$). The CPD causes the onset of an electrical force that is nullified by applying an external bias (${V}_{{DC}}$) with the same magnitude as the ${V}_{{CPD}}$. The applied external bias is equal to the difference of work functions between the tip (${{{\rm{WF}}}}_{{{\rm{tip}}}}$) and the sample ($({{{\rm{WF}}}}_{{{\rm{sample}}}})$, defined as:

1 $${V}_{{DC}}={V}_{{CPD}}=\frac{{{{\rm{WF}}}}_{{{\rm{sample}}}}-\,{{{\rm{WF}}}}_{{{\rm{tip}}}}}{\left|q\right|}$$

where *q* is the charge of the electron. Therefore, it is possible to determine the local WF of the sample surface knowing the ${{{\rm{WF}}}}_{{{\rm{tip}}}}$^43^. See Figure S2 for detailed descriptions. In the Bruker instruments used here, the DC bias is applied to the tip.

Topography and CPD of our samples have both been measured using a Bruker Multimode 8 AFM or a Bruker Dimension XR Icon. For KPFM experiments, we used commercial tips SCM-PITv2 from Bruker, where the cantilever is made of silicon and the tip is coated with Pt-Ir to make it conductive. The nominal tip radius is 25 nm. This cantilever has a resonance frequency *f*_0_ = 75 kHz and a spring constant *k* = 3 N.m^-1^. Our data were acquired with the PF-KPFM-AM mode. Topography and CPD were acquired within two passes above the surface. The tip performs a first pass recording the topography, then lifts up at a given Lift Scan Height (LSH) and acquires the CPD, following the recorded topography of the first pass. Topography was measured by PeakForce Tapping® technique where the tip periodically approaches the sample until the force setpoint is reached. It allows for scanning of delicate surfaces, such as biomolecules, without dragging them. CPD data were acquired with the AM mode during the second pass, the lock-in amplifier working in Amplitude Modulation (AM) mode. Experiments were carried out at slow rate (500 nm.s^-1^), for 512 × 512 pixel images. LSH is set at 15 nm for single ferritin samples and 20 nm for ferritin monolayer samples. This value was chosen to fulfill two conditions: fly closely over the surface in order to probe accurately the electrostatic contribution from the ferritin^69^, and at the same time, avoid possible surface contact, which generates image artefacts^70^.

### Fourier transform infrared spectroscopy (FTIR)

Differential FTIR experiments have been carried out using a Bruker Tensor 27 FTIR spectrometer equipped with an air-dryer SDAT from Specken Drumag. Prior to sample measurement, a clean silicon substrate cut from exactly the same wafer as the sample substrate, was measured as a reference sample. Then, a drop of ferritin solution or HEPES buffer was deposited on the same silicon wafer for 2 h. Sample absorbance spectra was measured on 500 scans for a 4000–800 cm^−1^ wavelength range. Spectrum were recorded in transmission with an incidence angle of 70° (Brewster angle) through the silicon substrate.

### Combined Quartz Crystal Microbalance with dissipation (QCM-D) and Localized Surface Plasmon Resonance (LSPR)

QCM-D and LSPR measurements were carried out using the Q-Sense E1 module with Q-Sense sapphire window module (Biolin Scientific) coupled with the Insplorion Acoulyte module (Insplorion AB). Measurements were conducted using Acoulyte AT-cut quartz crystal sensors (Insplorion AB), which are QCM-D compatible substrates featuring randomly distributed gold nanodisks (100 nm diameter, 20 nm height) coated with a thin silica layer (5–10 nm). The interaction of light with these nanodisks induces a collective oscillation of conduction electrons—known as Localized Surface Plasmon Resonance (LSPR)— resulting in a pronounced absorption peak centered around 700 nm. Variations in the refractive index near the sensor surface lead to shifts in the LSPR peak position (denoted as Δλ_max), enabling real-time monitoring of biomolecular interactions. Frequency and dissipation changes were analyzed using QTools software (v3.1.33.567, Biolin Scientific, Sweden), while LSPR signal analysis was performed with Insplorer software (v1.3.1, Insplorion AB, Sweden).

## Supplementary information

Supplementary Information

Transparent Peer Review file

## Data Availability

The data generated in this study have been deposited in the Recherche Data Gouv database [https://recherche.data.gouv.fr/fr]. The dataset for the FTIR spectra, the AFM and KPFM images and related profiles, the QCM data are available at https://doi.org/10.57745/LSB5EU. Received: ((will be filled in by the editorial staff)) Revised: ((will be filled in by the editorial staff)) Published online: ((will be filled in by the editorial staff))
