# Supplementary material for: Direct measurement of protein dipoles reveals the mechanism for like-charge adsorption on silica
Source: Nat Commun. 2026 Sep 15;17:10860. doi: 10.1038/s41467-026-76501-8 (PMC13672354; doi:10.1038/s41467-026-76501-8)
Supplement: Supplementary file 1 [file 41467_2026_76501_MOESM1_ESM.pdf]

Supplementary Information to „Direct Measurement of Protein Dipoles Reveals The Mechanism For Like-Charge Adsorption on Silica"

Luis Lechaptois[1,2], Antoine Miche[3], Qingao Li[2], Souhir Boujday[3], Sierin Lim[2]*, Olivier Pluchery[1]*

[1] Institut des Nanosciences de Paris (INSP), Sorbonne Université, CNRS, UMR7588, 4 Place Jussieu, 75005, Paris, France
[2] School of Chemistry, Chemical Engineering and Biotechnology, Nanyang Technological University, 70 Nanyang Drive, Singapore 637457
[3] Laboratoire de Réactivité de Surface (LRS), Sorbonne Université, CNRS, UMR7197, 4 place Jussieu, 75005 Paris, France

*Corresponding authors: olivier.pluchery@insp.jussieu.fr / SLim@ntu.edu.sg

Content

1. AFM characterization of the roughness of the initial silica surface
2. Calibration of the KPFM tip on Bruker's standard sample
3. AFM and KPFM images of ferritin nanocages on silica
4. Lateral expansion of the ferritin nanocages when its heigh decreases
5. KPFM images of iron nanoparticles on silica
6. KPFM images of monolayers of ferritin nanocages at different loadings
7. AFM images of the immobilization of ferritin on silica as a function of the sodium concentration in the buffer solution
8. Control KPFM experiments on silica + HEPES, NaCl. Role of the sodium cations
9. AFM topography and surface density of ferritin in the monolayer
10. Comparison of the amount of ferritin immobilized on silica in stop-flow configurations with QCM-D
11. D−F plots for the different iron loadings in QCM-D
12. Estimation of acoustic and optical mass from QCM-D/LSPR measurements
13. Calculation of the electrostatic surface charge of AfFtn-AA in solution

1. AFM characterization of the roughness of the initial silica surface

The silica surfaces in our study correspond to the thermal oxide grown on silicon substrates. We have verified the quality of these surfaces with AFM, after the initial cleaning and it is shown in Figure S1. The image was recorded with KPFM tip SCM-PIT-v2® tip which as nominal radius of curvature 25 nm. It exhibits an RMS roughness of 0.14 nm, which makes this surface very suited to detect the presence of ferritin proteins of diameter of 12 nm.

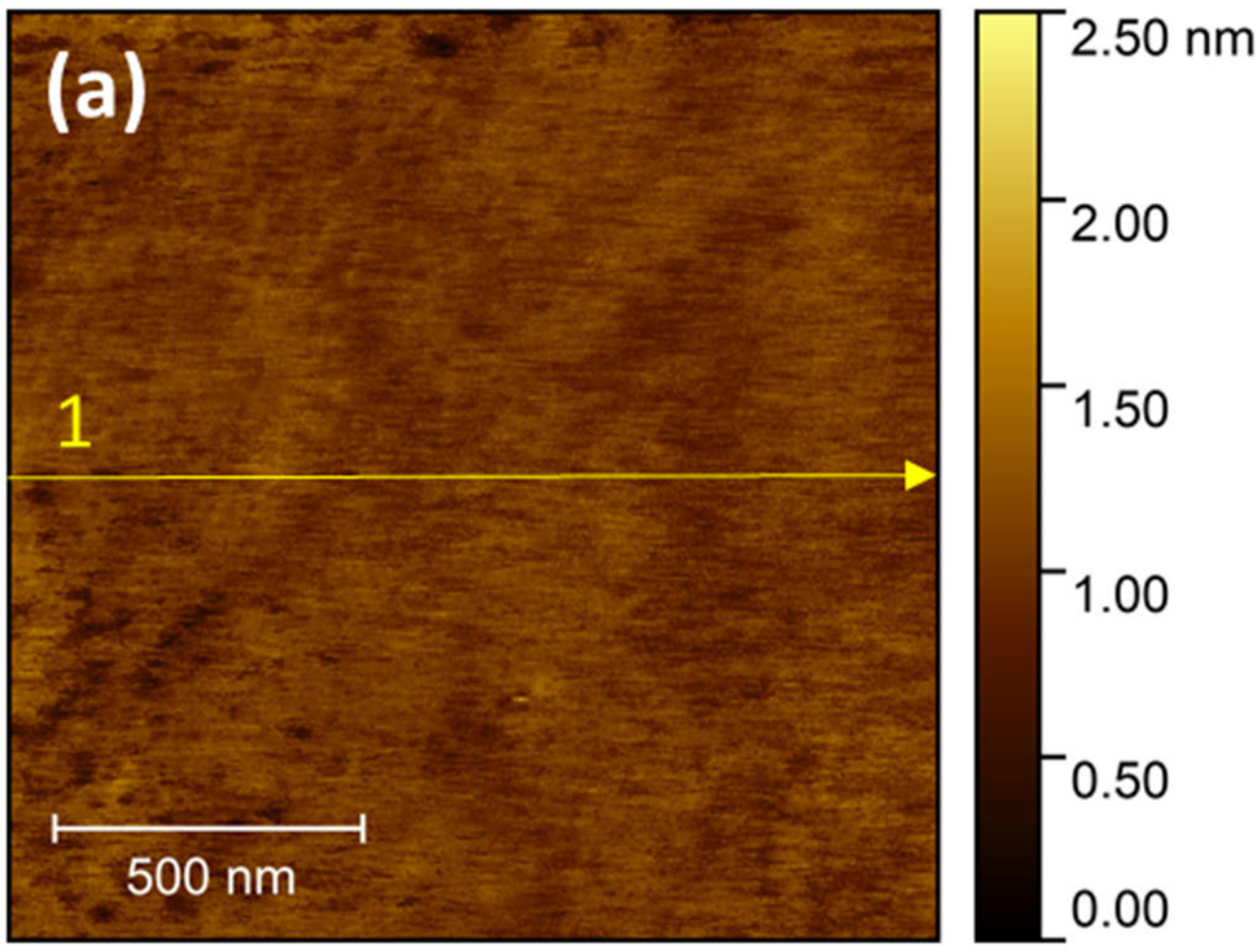

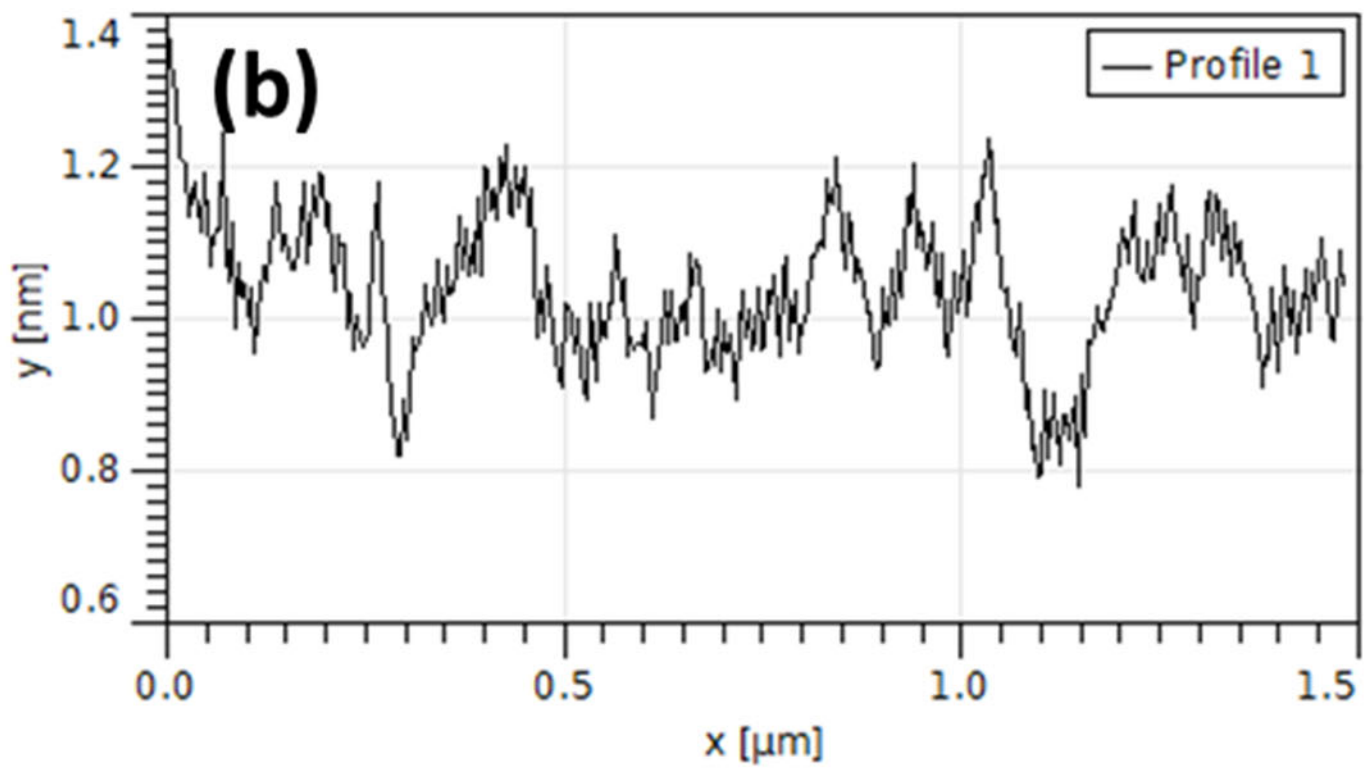

Figure S1 : (a) Clean silica surface analyzed with AFM equipped with a SCM-PIT-v2® tip (nominal radius of curvature 25 nm). The RMS roughness of this surface is 0.14 nm. The initial surface does not exhibit any protrusion and any grain. (b) topographic profile across the surface

2. Calibration of the KPFM tip on Bruker's standard sample

The main data obtained from the KPFM experiments are values of contact potential difference (CPD) that results from a difference between the work function of the sample and the work function of the tip. It is essential to ensure that this difference is computed with the correct sign and also to calibrate the value for the tip. To calibrate the Pt-Ir conductive tips used for our experiments, we performed measurements using the so-called PF-KPFM mode on the Bruker's standard sample (PFKPFM-SMPL), which is made from a silicon substrate specifically patterned with gold and aluminum stripes. The PF-KPFM mode is a double pass mode, where the topography is first acquired in Peak-Force tapping® mode and the surface potential is recorded during the second pass with a Frequency Modulated (FM) lock-in detection, after the tip was lifted at a height of 115 nm. Data exhibit clear and stable CPD and work function patterns. An example of calibration results is shown in Figure S2 where the topography images and the CPD images (Figure S*2*-a, b) are visible along with the associated height and CPD profiles (Figure S*2*-c). Here, CPD is measured at –1.1 V on the aluminum side. The work function of the gold being set at 5.0 eV, the tip work function is found around 5.0 eV, using equation 1 (Method section in the main text). Table S 1 gives the values of the CPD

measured on this reference sample which allows calculating the values of the work function of gold, alumina thin films and silica thin films in air.

In our experiments we have systematically carried out CPD measurements of the Bruker reference sample before and after scanning over the surfaces with ferritin in order to make sure the tip did not change during scanning and we disclosed results when contaminations or damages occurred which was extremely rare.

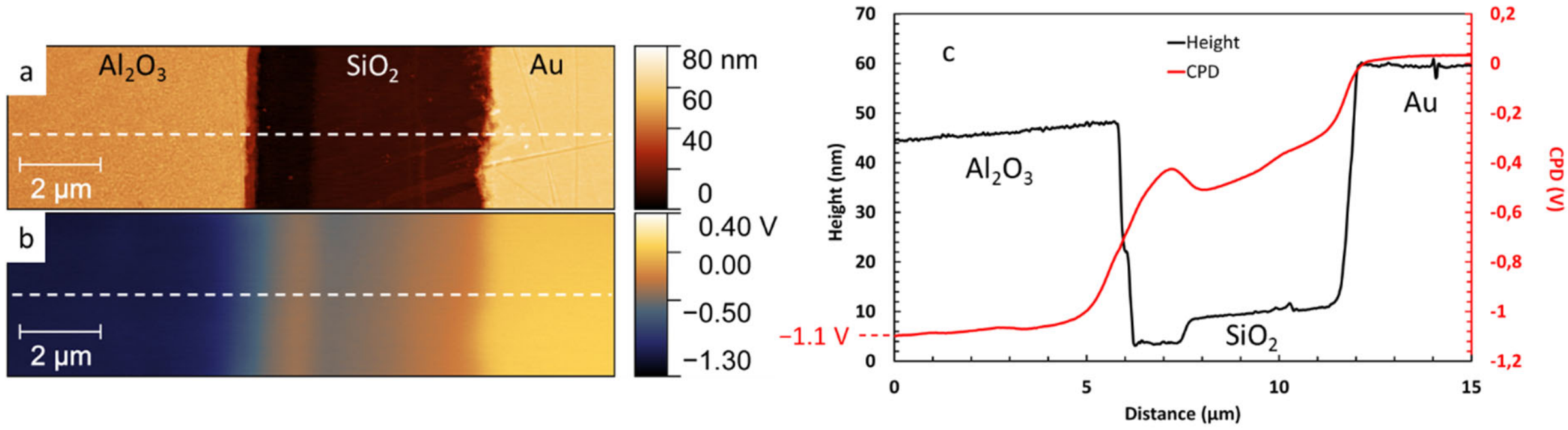

Figure S2: Topography (a) and relative CPD (b) images (15 x 4 µm²) of PFKPFM-SMPL Bruker's reference sample. (c) Height (black) and CPD (red) profiles taken across the surface. The LSH is set at 115 nm.

Table S 1. CPD values measured on the reference sample from Bruker and the resulting values of the work function for gold silica and alumina thin films. KPFM is performed within the PF-KPFM mode. The values are calculated assuming the work function of gold is 5.0 eV in air.

	CPD	***WF***
Au	+0.030 V	5.0 eV
Si/SiO₂	−0.430 V	4.54 eV
Al/Al₂O₃	−1.100 V	3.97 eV

3. AFM and KPFM images of ferritin nanocages on silica

Topography and CPD images obtained from KPFM measurements of ferritin nanocages (AfFtn-AA) on silica are shown below for all the iron loadings of the present study (Figure S3 and Figure S4). Images show a 1µm² area of the sample surfaces where AfFtn-AA have been deposited. Height distributions and averages CPD in the main text are computed from the Figure S3 and Figure S4, respectively.

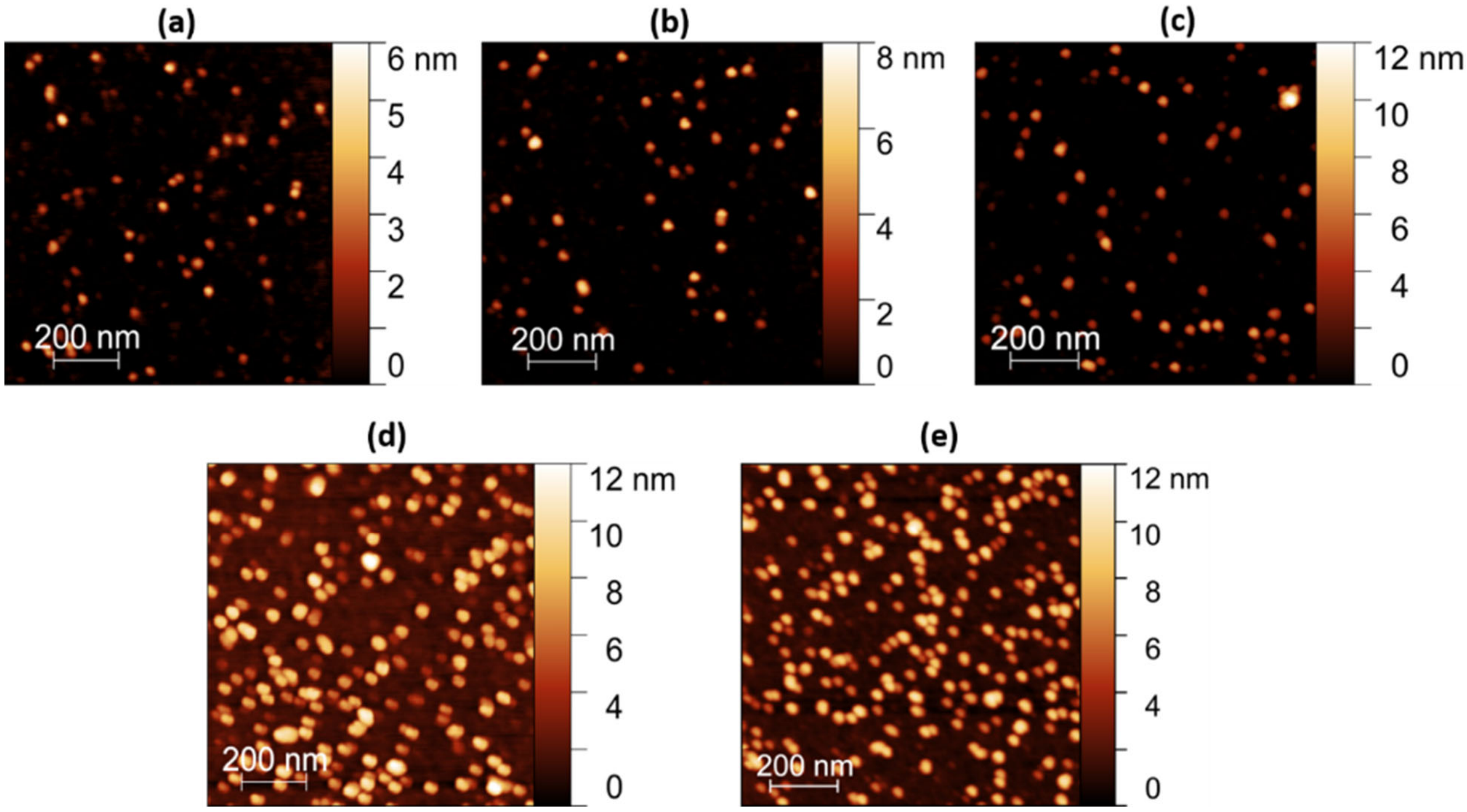

Figure S3 : Topography images (1 × 1 µm²) of ferritin with different iron contents deposited on silica, obtained by KPFM measurements. (a) Fe 600; (b) Fe1200; (c) Fe2400; (d) Fe3600; (e) Fe4800.

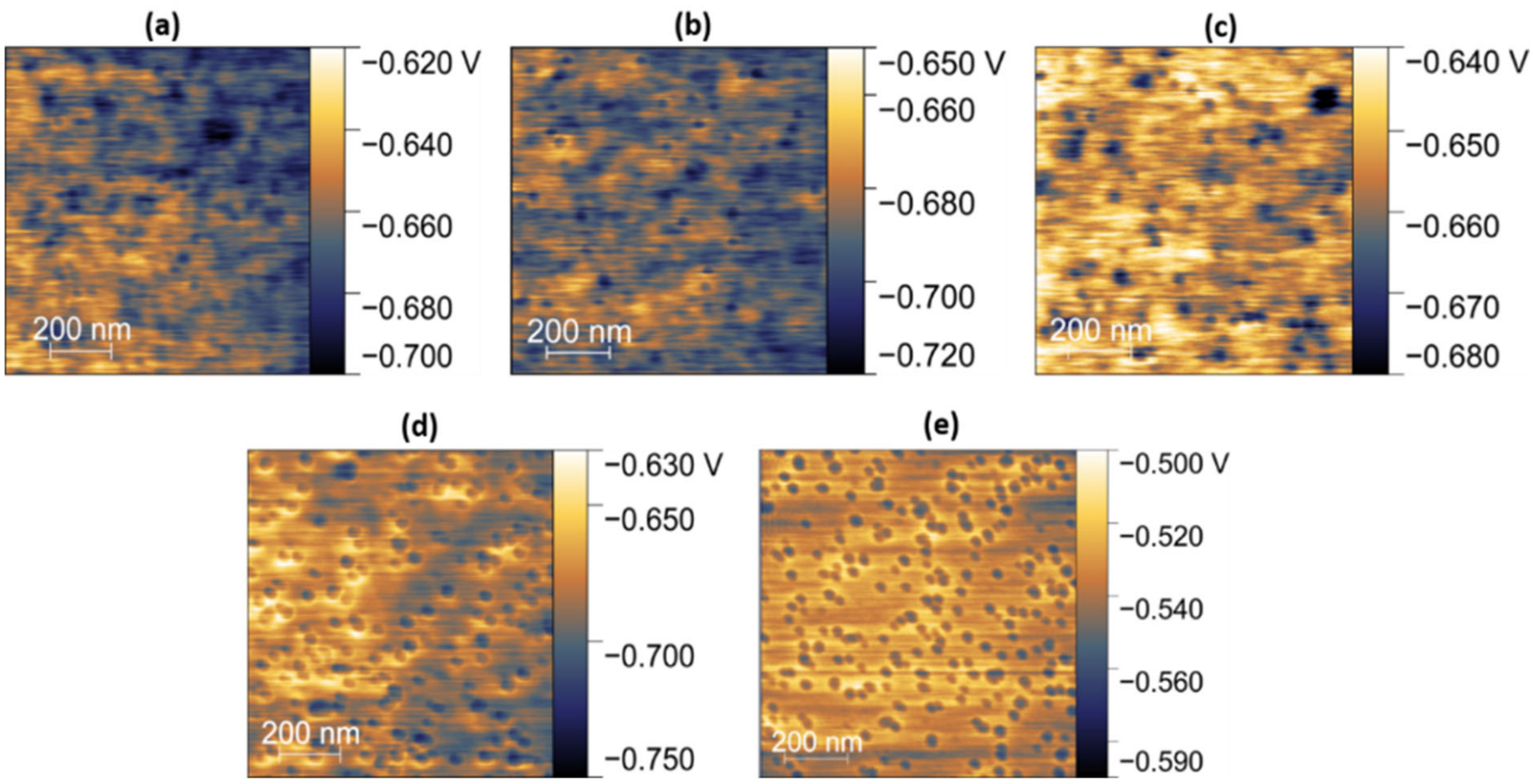

Figure S4: Relative CPD images (1 × 1 µm²) of ferritin with different iron contents deposited on silica, obtained by KPFM measurements. (a) Fe 600; (b) Fe1200; (c) Fe2400; (d) Fe3600; (e) Fe4800.

4. Lateral expansion of the ferritin nanocages when its heigh decreases

For all the loadings, the AfFtn-AA lateral width was measured at 32 nm (standard deviation of 1.5 nm) with a ~ 25 nm radius tip (SCM-PIT-V2). However, when the height of the ferritin decreases, we would expect to measure also an increase of the lateral size. How strong do we expect this lateral expansion to be?

We assume that the protein maintains a constant perimeter with variable heights and widths, adopting an elliptic profile. An ellipse is defined by its semi-major axis a and semi-minor axis b (see Figure S5). There is no exact analytical formula for calculating its perimeter, but we can use the following approximate formula, that is exact at 5% if a/b remains smaller than 3:

Perimeter $$p \approx 2\pi.\sqrt{\frac{a^2+b^2}{2}}$$

The (4800Fe)AfFtn-AA nanocage displays a profile in an ellipsoid shape with a height of 8.8 nm and a lateral size known to be 12 nm, and we can calculate the perimeter: p = 33.1 nm. The (600Fe)AfFtn-AA exhibits a flattened shape (height of 3.6 nm) and the lateral size is now 14.4 nm (see Figure S5). The ferritin undergoes a lateral variation of just +2.4 nm. This is not surprising that such a small lateral increase is not detected with the AFM due to the resolution limitation induced by the tip convolution. These values are summarized in Table S 2. The Figure S5 below shows a simple representation of two ferritin nanocages with different iron loadings immobilized on a surface. It results that this limited lateral size change is non-measurable with the AFM because the Pt-Ir coated tip used for these experiments exhibits a radius of ~ 25 nm.

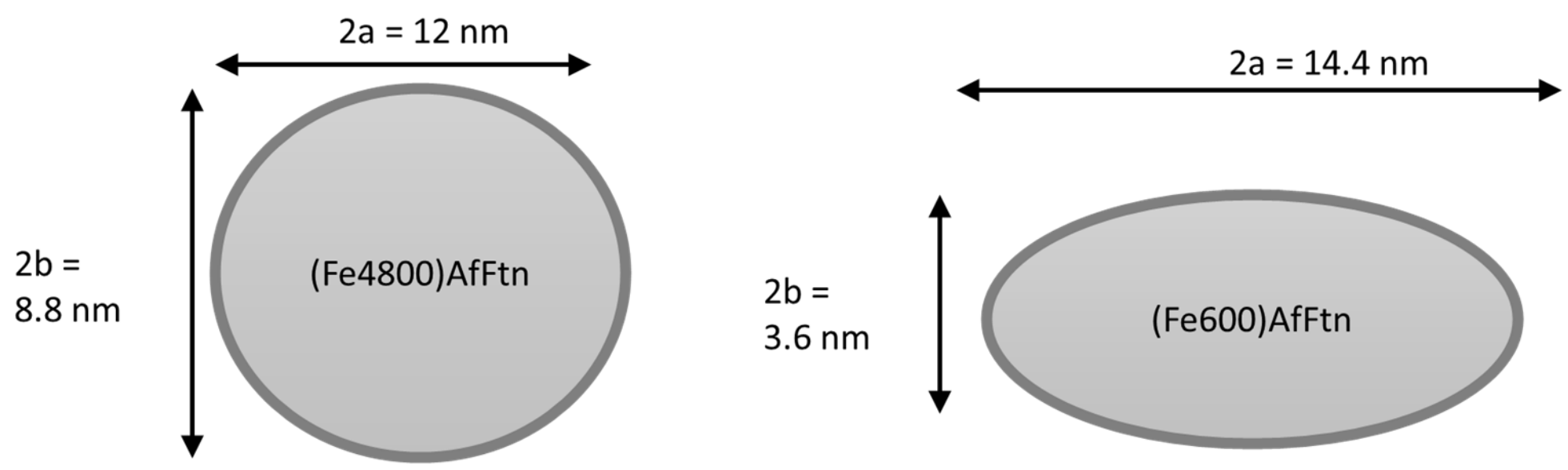

Figure S5 : Schematic of a ferritin nanocage loaded with Fe4800 (left) and Fe600 (right). The perimeter of the ferritin is supposed to remain unchanged ~ 33 nm. The height values (b) are obtained from the topography KPFM measurements.

Table S 2. Value of the heights of the ferritins measured with AFM and calculation of the corresponding width, if we consider that the perimeter remains the same for all the iron loadings.

**Ferritin**	**Fe600**	**Fe1200**	**Fe2400**	**Fe3600**	**Fe4800**
**Height, 2*b* (nm)**	3.6	4.8	6.5	7.9	8.8
**Width, *2a* (nm)**	14.4	14.1	13.4	12.6	12.0

From these observations, we performed lateral width measurements of ferritins using a sharper tip to obtain a better lateral resolution. Topography images of Fe4800 and Fe2400 ferritin nanocages (AfFtn-AA) on silica surfaces were recorded with a ~ 2 nm radius tip (ScanAsyst-Air® – silicon nitride-coated tip). Results are shown in Figure S6 and show images of a 500 x 500 nm² area of both samples, with zoom-in on a single ferritin particle. The height profiles across one ferritin particle from both samples were overlapped and shown on Figure S6-c. The profiles describe ferritin particles with similar full width at half maximum (FWHM) around 20 nm. Furthermore, FWHM were measured from topography images and the averages calculated (on 30 ferritin particles) at 18.6 ± 1.1 nm and 20.7 ± 1.4 nm for Fe4800 and Fe2400, respectively. These results show a small lateral variation of about ~ 2 nm that is coherent with the lateral width variation between Fe4800 and Fe2400 of +1.4 nm, calculated above (see Figure

S6). Therefore, the lateral expansion of ferritin particles upon adsorption on silica surfaces can be observed by using a very sharp tip, but with which we could not perform surface potential measurements.

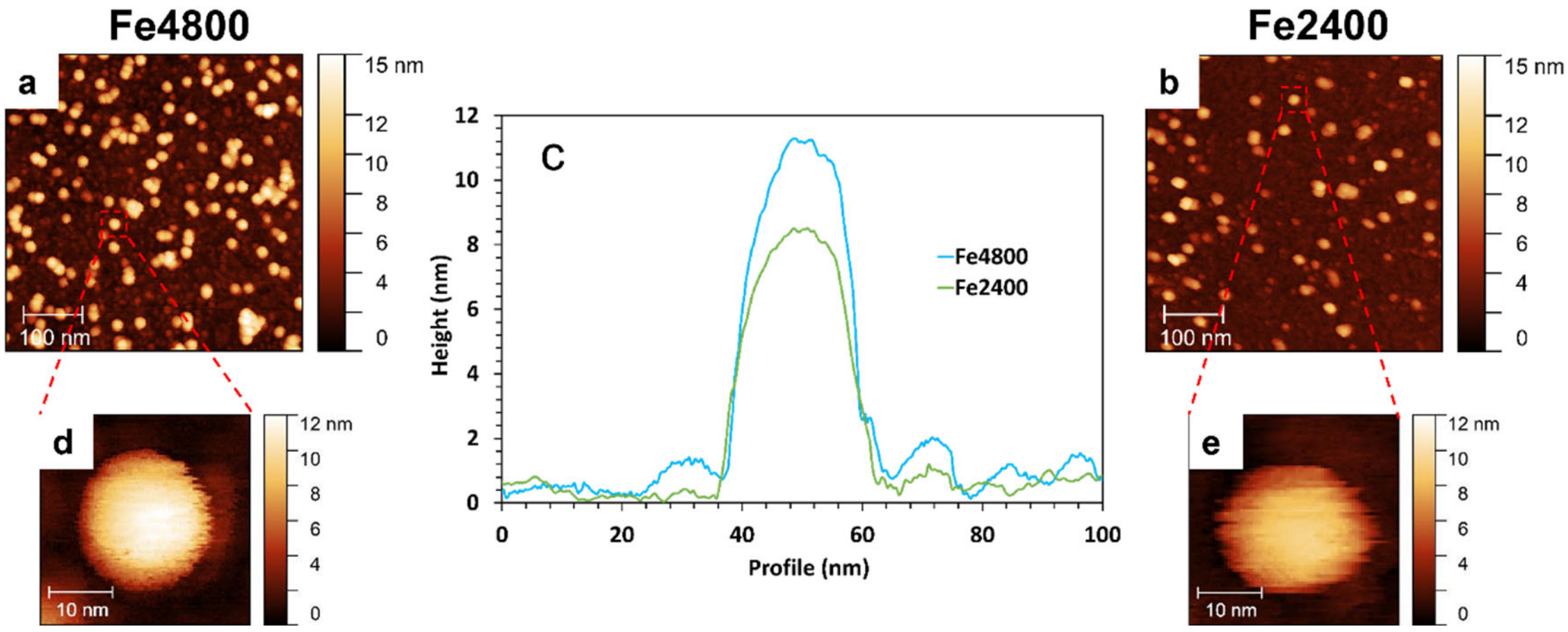

Figure S6 : Topography images of (a) Fe4800 ferritins and (b) Fe2400 ferritins on silica surface, recorded with a ~ 2 nm radius tip (ScanAsyst-Air®). (c) Height profiles over one ferritin Fe4800 (blue) and one ferritin Fe2400 (green) on silica surface. For the sake of clarity, Y scales are shifted to superpose both curves. (d) Single Fe4800 ferritin. (e) Single Fe2400 ferritin.

5. KPFM images of iron nanoparticles on silica

The Figure S7 below shows the topography and CPD images obtained by measurement of single iron nanoparticles (FeNPs) and FeNPs monolayer with the KPFM. Height and CPD profiles are shown.

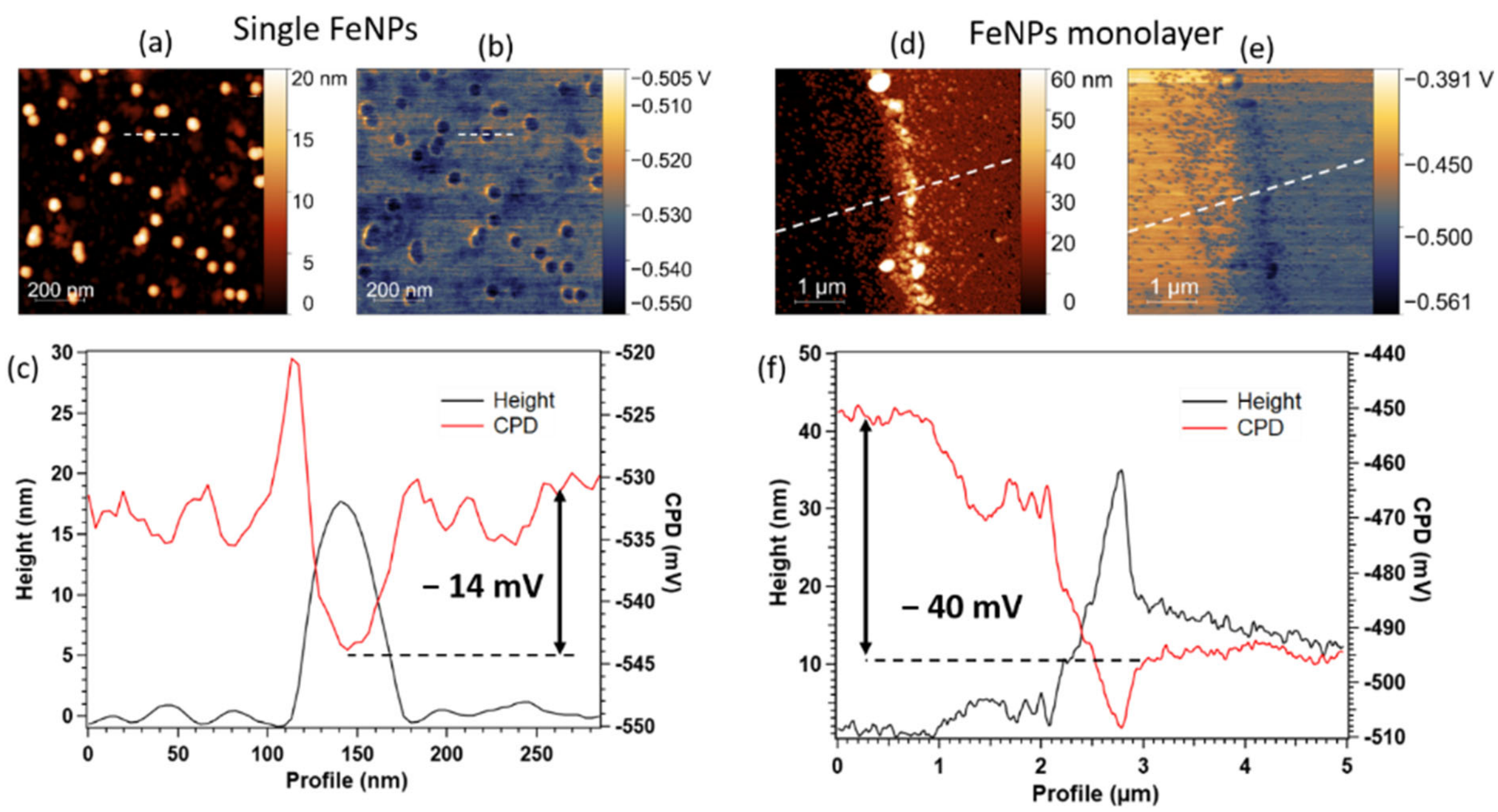

Figure S7: Topography (a) and relative CPD (b) images of FeNPs on Si. (c) Height (red) and CPD (black) profiles taken on one FeNP (from white dashed line). Topography (d) and

relative CPD (e) images of FeNPs monolayer on Si. (f) Height (red) and CPD (black) profiles (f) (white dashed line).

6. KPFM images of monolayers of ferritin nanocages at different loadings

Topography and CPD images obtained from KPFM measurement of AfFtn-AA monolayer deposited on silica are shown below (Figure S8). Images show a 5 × 5 µm² area of samples surface where ferritins have been deposited as a monolayer. CPD profiles (Figure 4.d) in the main text are from the CPD images (f-j) and profiles are taken perpendicular to the silica/AfFtn-AA boundaries.

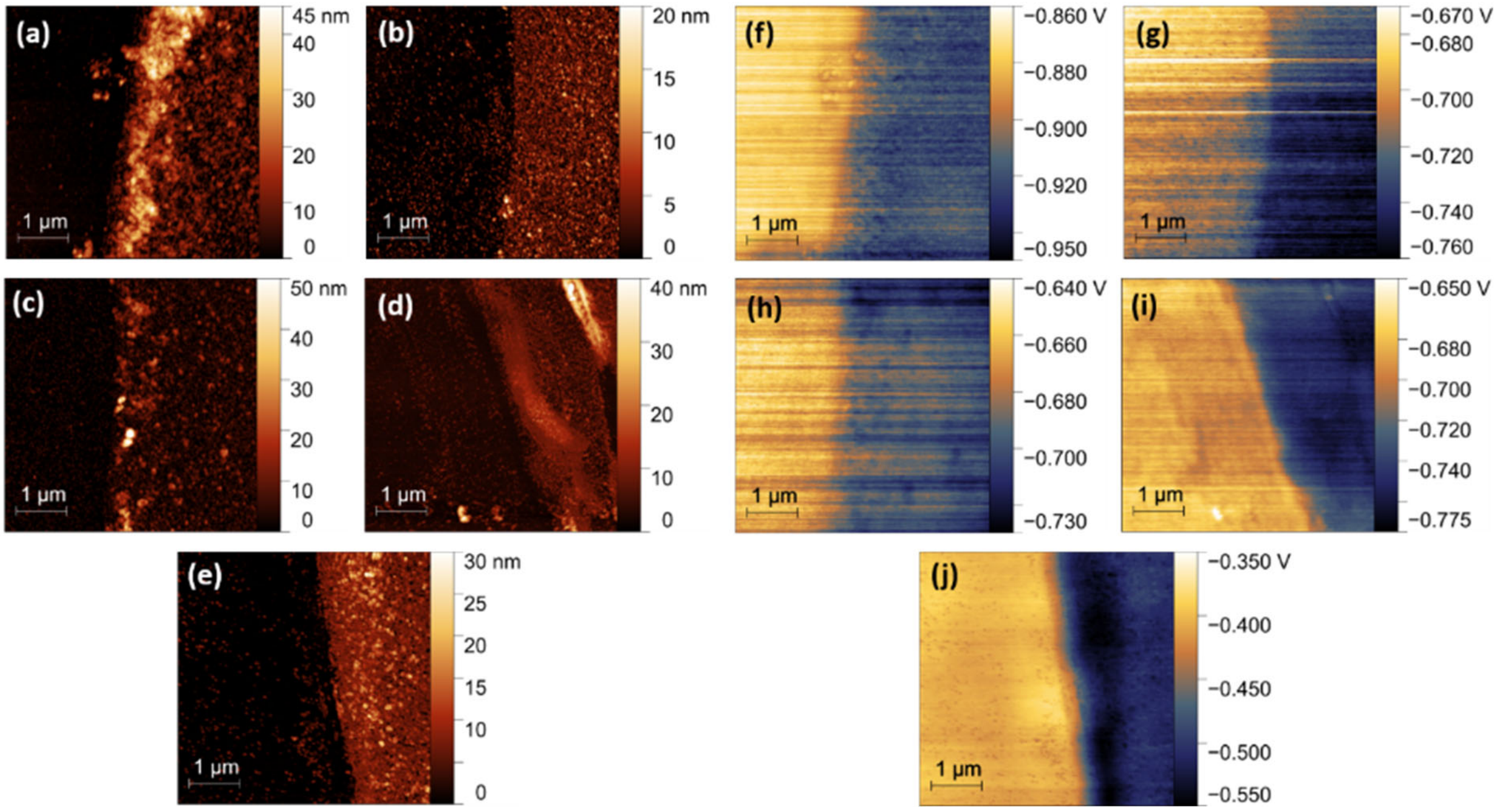

Figure S8: Topography (a-e) and relative CPD (f-j) images (5 × 5 µm²) of ferritin monolayer with different iron content deposited on silica, obtained by KPFM measurements. (a)(f) 4800Fe; (b)(g) 3600Fe; (c)(h) 2400Fe; (d)(i) 1200Fe; (e)(j) 600Fe.

7. AFM images of the immobilization of ferritin on silica as a function of the sodium concentration in the buffer solution

In order to test the role of the NaCl in the buffer solution for immobilizing ferritin on silica, we have carried out AFM experiments, where (Fe4800)AfFtn-AA was dissolved in buffer solutions containing different concentrations of NaCl, ranging from 400 mM down to 0 mM. The actual concentration of sodium cations Na+ was evaluated, taking into account the addition from the NaOH solution used for adjusting the pH. For each drop-cast, a minimum of three areas of 1 × 1 µm² were recorded with the AFM at different locations on the sample. The average surface concentration along with the corresponding standard deviations are indicated in Table S 3 below and are plotted in Figure 2f of the main text.

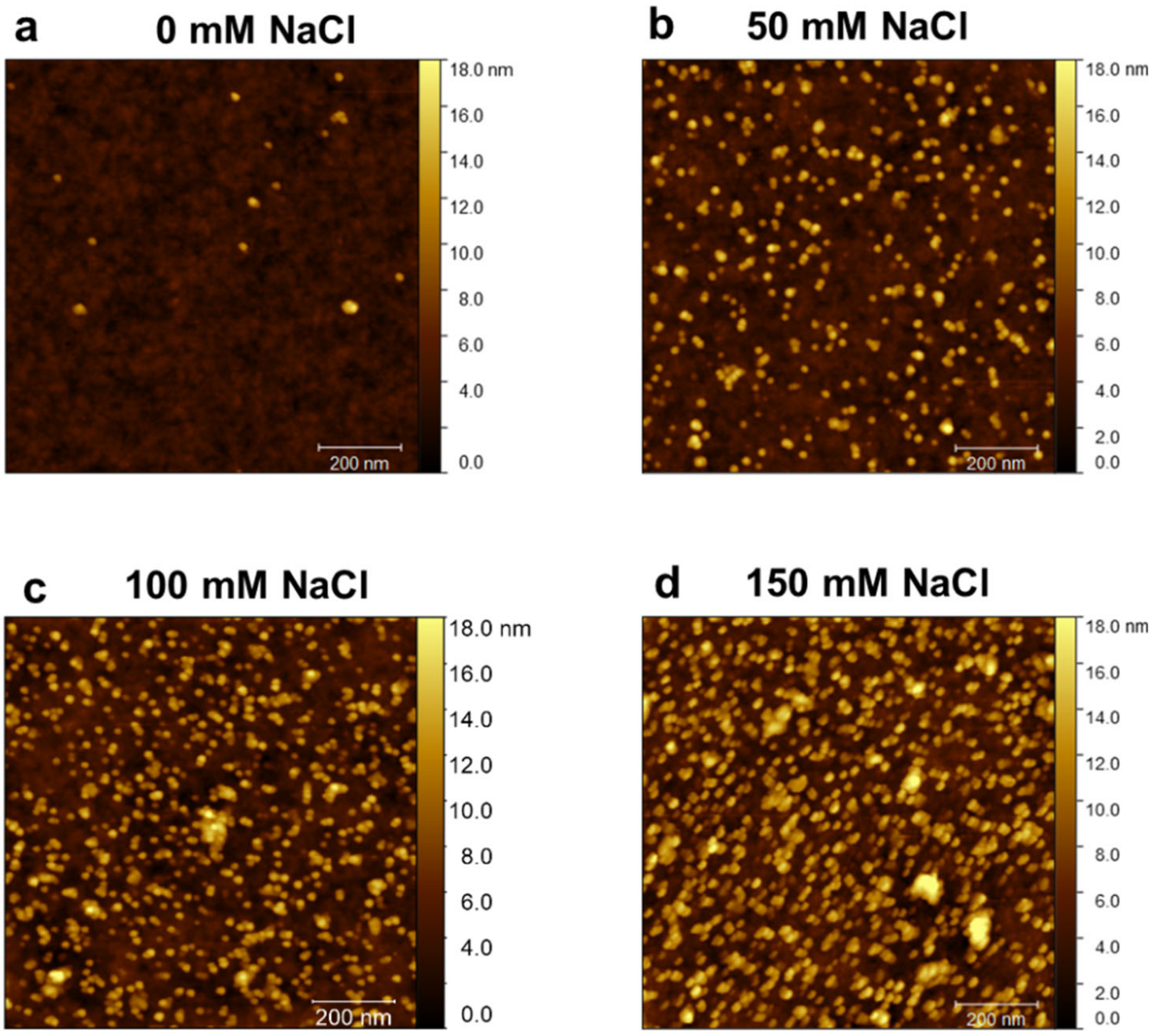

Figure S9: AFM image 1 × 1 µm² of AfFtn-AA drop-casted for 1 min on silica, when the buffer solution contained respectively (a) 0 mM NaCl, (b) 50 mM NaCl, (c) 100 mM NaCl and (d) 150 mM. AFM image were recorded in PeakForce tapping mode with a ScanAsyst-Air® tip from Bruker (nominal radius is 2 nm).

Table S 3. Surface coverage of (Fe4800)AfFtn-AA for increasing concentrations of Na^+ .

NaCl concentration	0 mM	50 mM	100 mM	150 mM	400 mM
Na^+ concentration	11 mM	61 mM	111 mM	161 mM	411 mM
Surf conc. (Ftn/µm²)	47	374	667	836	836
Std dev (Ftn/µm²)	± 40	± 15	± 33	± 49	± 148

8. Control KPFM experiments on silica + HEPES, NaCl. Role of the sodium cations

Control experiments were carried out with the HEPES buffer solution in order to verify if the CPD of the silica surface is modified by ions and counterions deposited by this buffer solution. The HEPES buffer solution was prepared with 50 mM of NaCl (See Methods section of the Main Text).

First, the CPD map of the clean silica surface was characterized with KPFM as shown in Figure S10. The surface exhibits an average value for the CPD of −645 mV.

This silica surface is made of silanol and silanolate groups and some water molecules as depicted in Figure S10d and following the model published by the group of S. Roke. [1]

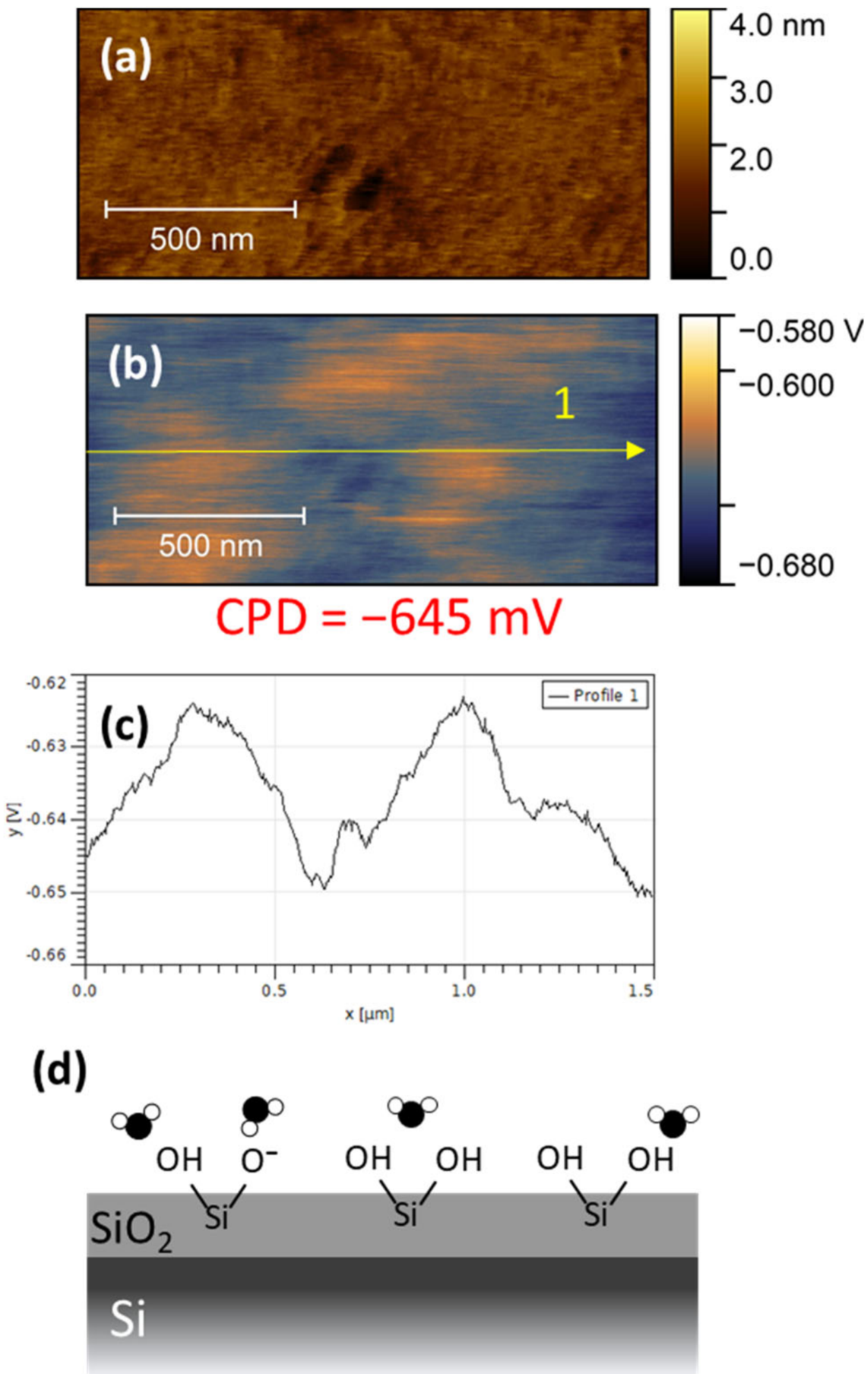

Figure S10: Characterization of the initial, clean silica surface with KPFM over 1.5 × 0.7 µm² showing (a) the topography and (b) the CPD map recorded with a LSH of 40 nm. The average value of the CPD is −645 mV. (c) CPD profile along line 1, showing that the potential is homogeneous with slow fluctuations over distances of no more than 20 mV over 200 nm. (d) Sketch of the silica surface in air showing some silanol and silanolate end groups and water molecules.

In a second stage, a drop of the buffer solution was deposited on two identical silica substrates and left for 2 hours (See Figure S11-a), protected from evaporation by a petri dish. Then, for the first sample, the buffer solution was blown away and dried without any rinsing. The AFM and KPFM images (Figure S11-b and c) show that the roughness did not change, whereas the CPD values decreased of 240 mV and reached an average CPD value of −885 mV. The second sample was rinsed with deionized water and blown dried prior to AFM and KPFM characterizations. The results are shown in Figure S11-d and e. The surface roughness did not evolve and the CPD value increased of just +20 mV compared to the initial surface. The different values are summarized in Table S 4. These experiments were carried out with the same KPFM tip (SCM-Pit-v2®) and within the same day, so that these CPD values can be compared.

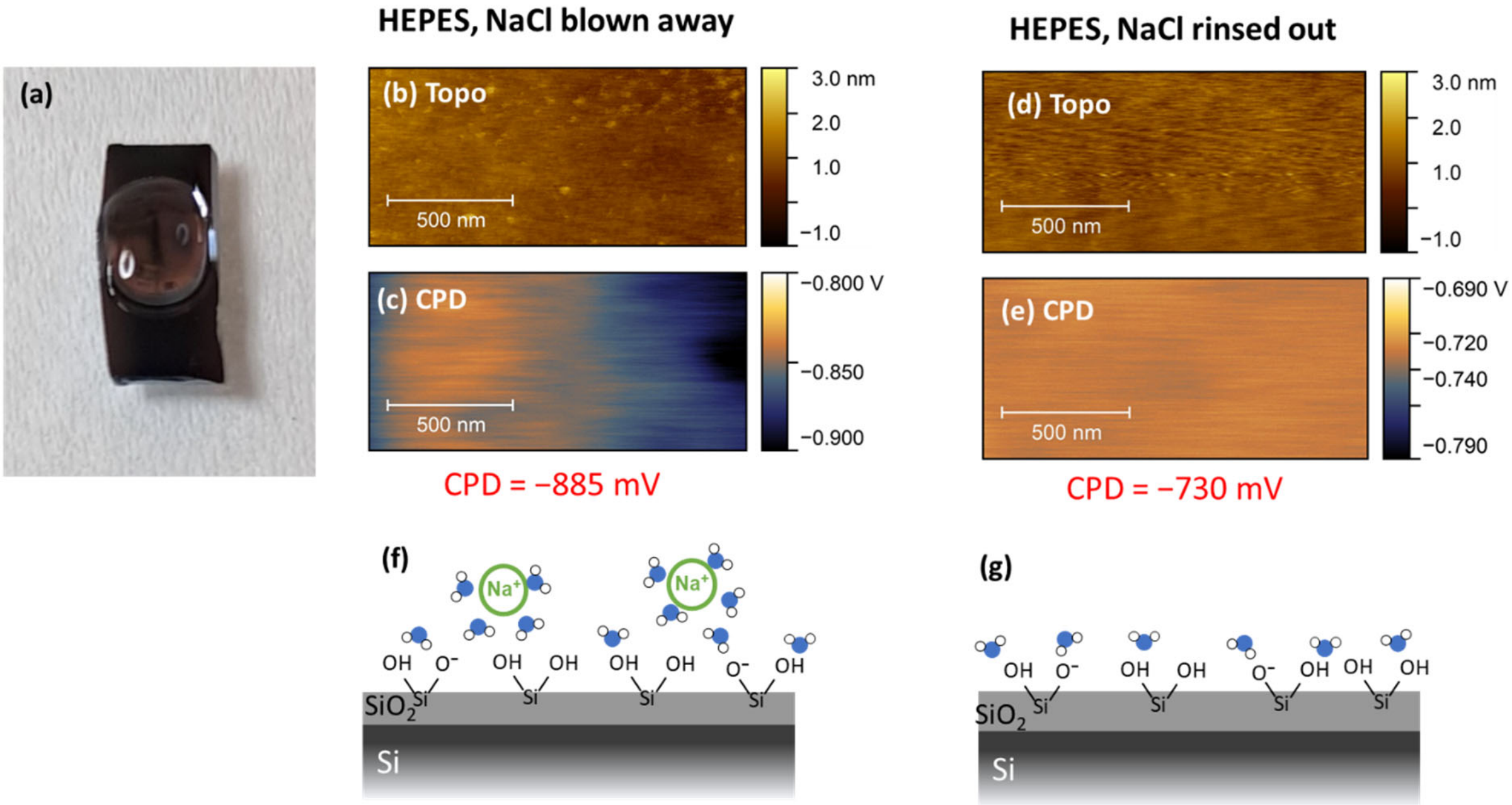

Figure S11 (a) Silica surface with a drop of buffer solution (HEPES, NaCl). (b) AFM image (1.5 x 0.7 μm²) of the surface after the drop was blown away, without any rinsing. (c) CPD map of this same surface. The average CPD value is measured at −885 mV. (d) AFM image (1.5 x 0.7 μm²) of the surface after the drop was rinsed with DI water. (e) CPD map of this same surface after rinsing. The average CPD value is now −730 mV. Both CPD maps are recorded with a LSH of 40 nm. (f) Schematic representation of the surface when the buffer is only blown away, leaving some Na+ counterions. (g) Schematic representation of the surface after rinsing, where the counterions have been entirely removed.

Table S 4. Variation of the CPD of the different surfaces

Sample name	*CPD (mV) clean*	*CPD after contact with HEPES, NaCl*	*CPD after HEPES, NaCl and rinsing*	*ΔCPD*
Si-HEPES 1	-645 mV	-885 mV		-240 ± 20 mV
Si-HEPES 2	-755 mV		-730 mV	+20 ± 20 mV

The CPD values obtained with these experiments show that the contact of the buffer solution of HEPES with NaCl does not modify the surface charge of the silica if it is rinsed with deionized water. However, the contact of HEPES, NaCl and their removing by blowing away the solution results in a strong decrease of the CPD values. If positive charges are left on the surface, we expect a decrease of the CPD values [2]. Therefore these results suggest that some Na+ counterions have been deposited on the surface as represented in Figure S11-f. This situation is confirmed by a recent study that shows that Na^+ ions are embedded in the Stern layer within the electric double layer at the surface of silica beads [1]. The presence of these positively charged counterions at the surface plays an essential role for explaining the adsorption of the negatively charged ferritins.

9. AFM topography and surface density of ferritin in the monolayer

The topography of the full monolayer of ferritin was recorded simultaneously to the CPD map at the edge of the ferritin drop. This topography is acquired with the SCM-PIT-v2® tip which exhibits a typical apex diameter of 25 nm. This lateral resolution is sufficient for

distinguishing the individual ferritin and count their surface density. The count is done manually as shown in Figure S12.

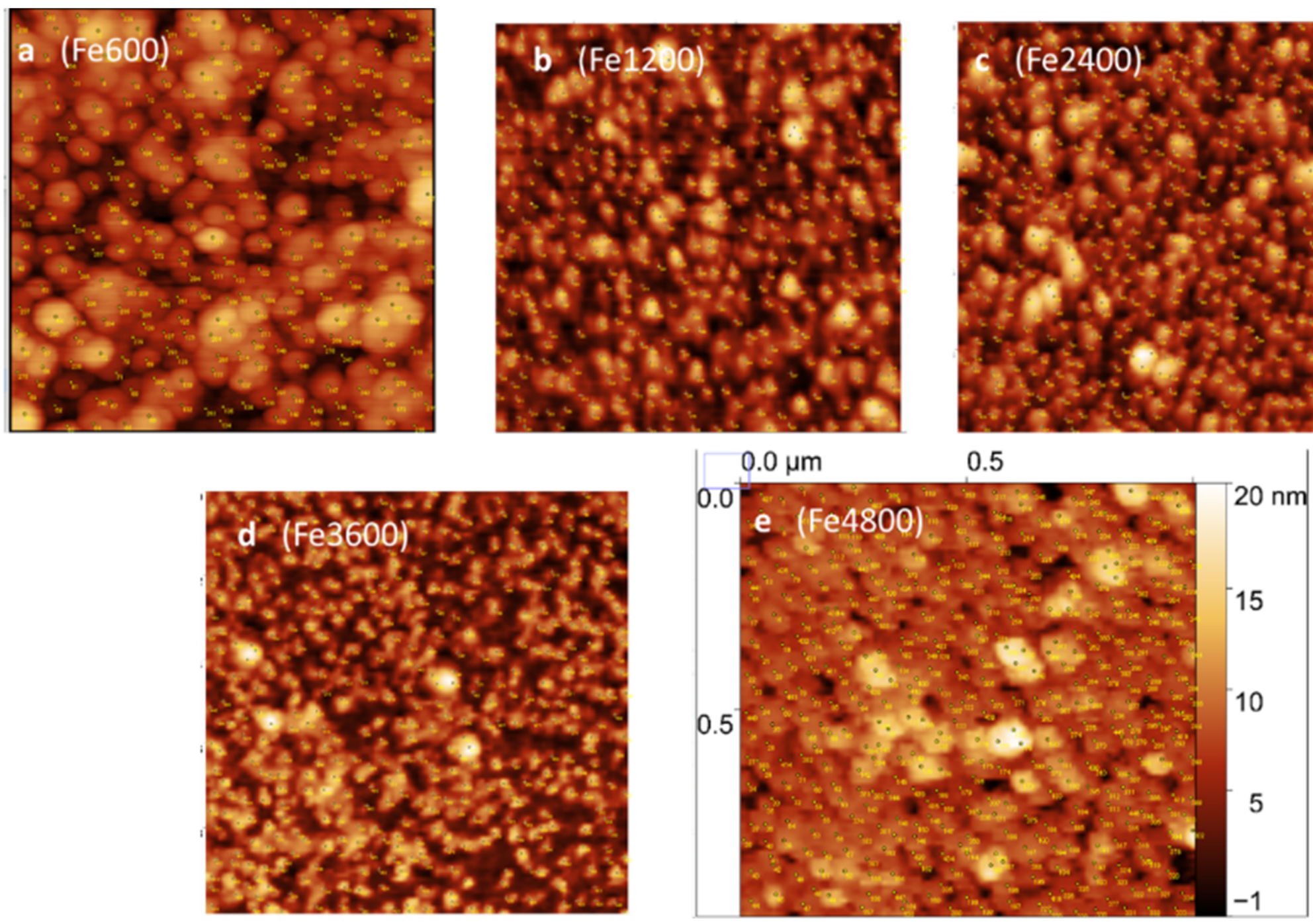

Figure S12: KPFM topography images (1 × 1 µm²) of ferritin monolayers obtained simultaneously to the CPD measurement. The surface coverage was counted as shown by the tagged nanostructures and it yielded the following results: **a.** Fe600: 290 NPs/µm²; **b**. Fe1200: Fe1200: 430 NPs/µm²; **c.** Fe2400: 500 NPs/µm²; **d.** Fe3600: 470 NPs/µm²; **e.** Fe4800: 450 NPs/µm².

10. Comparison of the amount of ferritin immobilized on silica in stop-flow configurations with QCM-D

QCM-D experiments were carried out in the stop-flow configuration, where the flow of solution containing the Fe4800 AfFtn-AA was stopped 15 min after introduction. Since the immobilization of Ftn is very weak, two cases were considered: the case where the Ftn was dissolved in a buffer solutions of 25 mM HEPES and 50 mM NaCl (pH 7.5) shown in Figure S13a and the case where the buffer solutions did not contain any NaCl (Figure S13b). In the presence of NaCl, the frequency shift is $\Delta f = -165$ Hz, which indicates a clear Ftn adsorption. Without NaCl, the frequency shift is very low and is barely measurable ($\Delta f = -2.5$ Hz) indicating almost no adsorption on the surface.

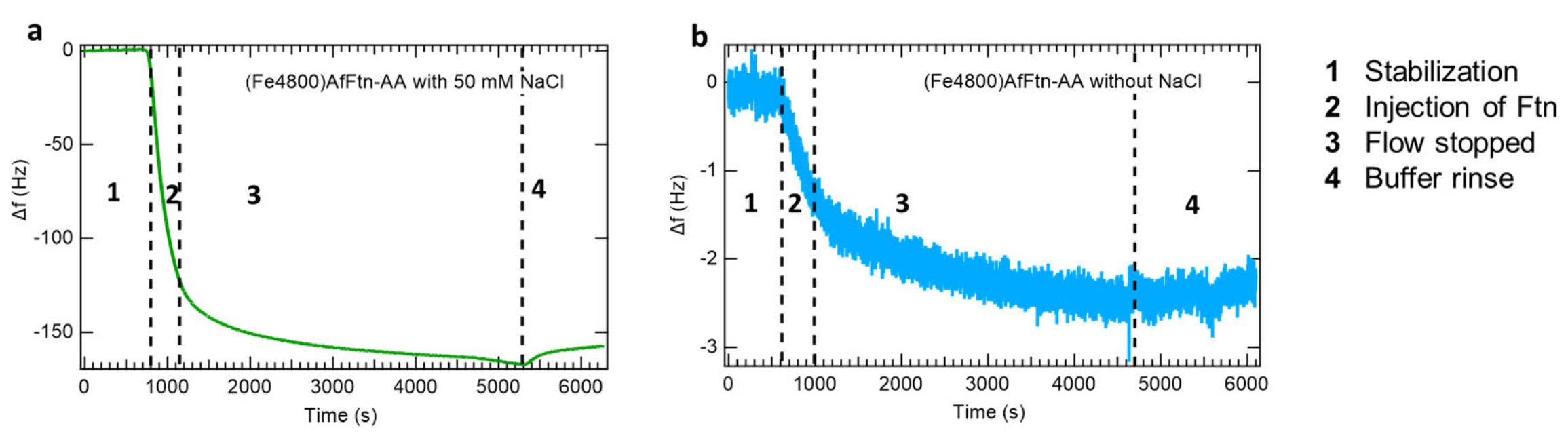

Figure S13: Frequency shift obtained with QCM performed in stop-flow experiments with 4800Fe AfFtn-AA immobilized on quartz. Each experiments includes four stages and the meaningful frequency shift is measured in stage 3. (a) The buffer solution contains HEPES and 50 mM NaCl. The frequency shift is $\Delta f = -165$ Hz. (b) The same experiment is carried out but with no NaCl in the buffer solution. The frequency shift is $\Delta f = -2.5$ Hz.

11. D−F plots for the different iron loadings in QCM-D

In the main text, the QCM-D data of Figure 5-a shows the shift in frequency upon ferritin adsorption, and the related dissipation increase. These data correspond to the case where the surface was functionalized with APTES in order to force the immobilization of Ftn on the quartz surface. These QCM-D experiments were carried out while the solution was flowing through the cell. By plotting the dissipation variation ΔD as a function of the frequency shift Δf, the curve exhibits a linear portion where the slope k was calculated as shown in Table S 5. In the Figure S14 below, the corresponding linear portions were plotted along with their linear fit. These plots provide information on the rigidity of the ferritin nanocages. As explained in the main text, small k values are characteristic of rigid layers, while higher values are related to viscoelastic ones.[3]

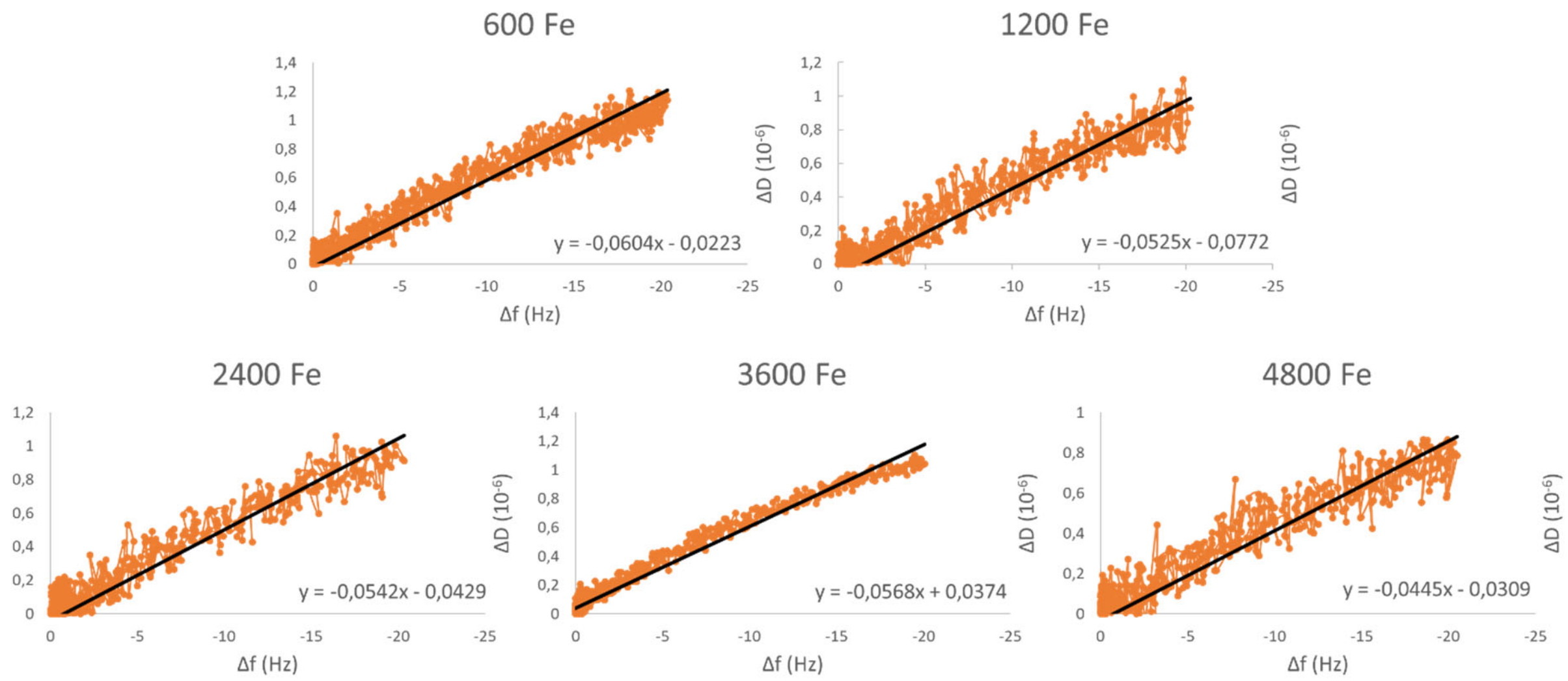

Figure S14: Zoom-in on the linear portion of the *D-f* plots for the various iron loadings, from which the slope *k* is calculated.

Table S 5. Values of the slope k obtained from the QCM-D experiments for the five iron loadings considered in the study

Ferritin	Fe600	Fe1200	Fe2400	Fe3600	Fe4800
k (10^{-6}.Hz^{-1})	0.060	0.053	0.054	0.057	0.045

12. Estimation of acoustic and optical mass from QCM-D/LSPR measurements

The acoustic masses were determined following the equation:

$$\Delta F = -n\frac{\Delta m_{QCM}}{C_f}$$

With: C_f *the 5 MHz crystal constant (17.7 ng/Hz/cm²) and n the harmonic number (1,3,5,7 …)*

The optic masses were determined following the equation:

$$m_{LSPR} = d_i \frac{\Delta\lambda_{NPS}}{S_0\left(e^{-2d_{i-1}/L_z} - e^{-2(d_{i-1}+d_i)/L_z}\right)dn_i/d_c}$$

With: **$\Delta\lambda_{NPS}$** the measured LSPR shift,
dn_i/d_c the refractive index increment for proteins. The refractive index of the flowing solution (HEPES) is n = 1.371 and the refractive index increment was taken as an average value for protein (0.185 mL/g).
d_i the thickness of the sample layer determined from QCM-D data.
d_{i-1} is the thickness of the starting layer, i.e. organic layer, and was approximated to nil.
S_0 is the sensitivity of the substrate (the gold nanostructured sensors are coated with a SiO2 layer, which displays an LSPR sensitivity of 60 nm/RIU).
L_z is the evanescent field decay length (30 nm, data from the sensor manufacturer).

Table S 6. Optical and Acoustic Mass of the Ferritin Layers *on APTES-modified nanostructured quartzs*

Sample	*600Fe*	1200Fe	*2400Fe*	*3600Fe*	*4800Fe*
Acoustic mass (ng/cm²)	1075	1101	1061	1093	1035
Optical mass (ng/cm²)	158	274	260	260	306

13. Calculation of the electrostatic surface charge of AfFtn-AA in solution

The electrostatic surface charge of the ferritin (AfFtn-AA) was numerically calculated using the PyMol software from Schrödinger®. This software is a system of molecular visualizations used for structural biology. From the protein data bank code (3KX9 for AfFtn-AA), it displays a 3D ribbon representation of one ferritin nanocage showing the 24 folded subunits (Figure S15.a) and indicates the position of each amino acid in the nanocage. In addition, the software calculates the electrostatic surface charge by solving the Poisson-Boltzmann equation. In our work, the electrostatic surface charge of one AfFtn-AA nanocage in an ionic solution that reproduces the HEPES buffer was calculated (i.e., a solution at pH 7.4 with monovalent counter-ions (Na^+ and Cl^-) at an ionic strength I = 50 mM) The was temperature set at T = 300 K, and the dielectric constants are set at ε_W = 78 and ε_f = 3.34 for water and ferritin, respectively. As a result, the software displays an electrostatic potential on the molecular surface. In Figure S15.b, the result for AfFtn-AA is shown and presents mostly a negative surface charge (in red). However, we can distinguish few positive areas (in blue) situated around some of the 3-fold axes (Figure S15.c), which originate from lysine amino acids. These latter are positioned on a helix at the exterior of the nanocage' shell, which gives easy access to the solvent. Furthermore, the lysine has a long alkane side chain terminated with a positively charged amino group (NH_3^+) at physiological pH.

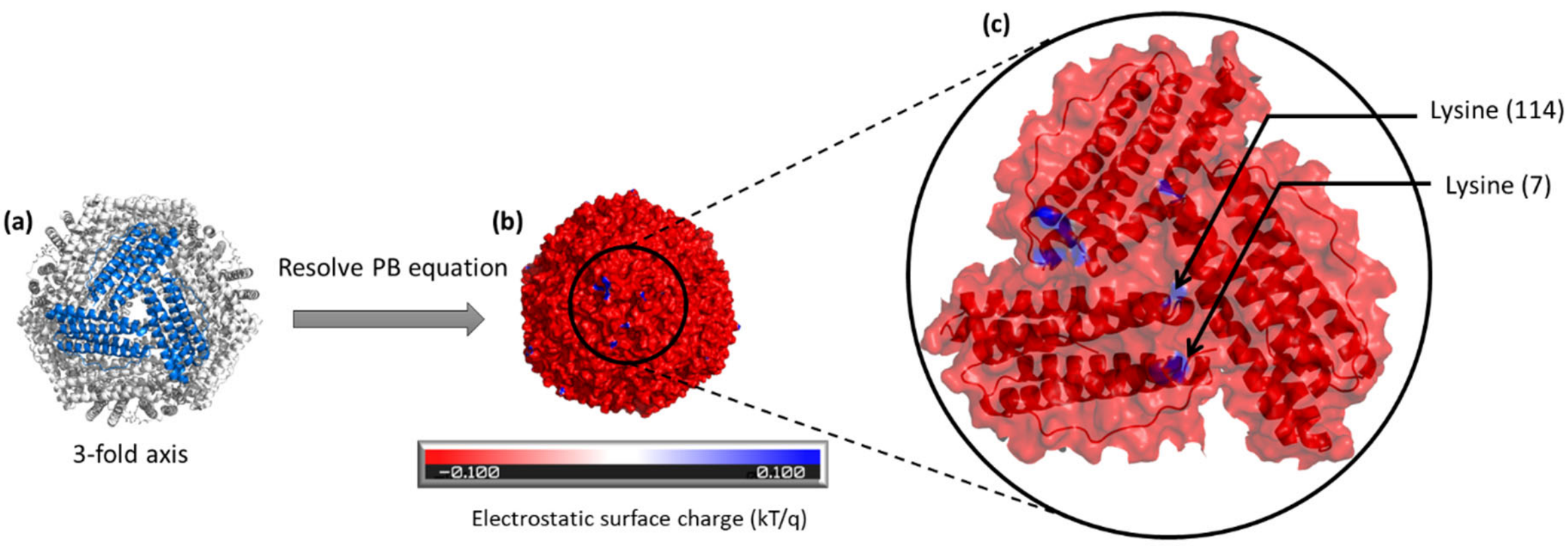

Figure S15: 3D PyMol representations of ferritin AfFtn-AA at 3-fold axes. (a) ribbon representations of AfFtn-AA. The subunits forming the 3-fold are indicated in blue. (b)

representations of the electrostatic potential on the molecular surface after resolution of the Poisson-Boltzmann (PB) equation. The positive areas are in blue, and the negative areas are in red. The scale is shown at the bottom and ranges from −0.1 to +0.1. (c) Zoom-in on the 3-fold axis indicates position of the lysines. The number corresponds to the position of the amino acid in the primary structure.

Supplementary References

1 Marchioro, A. *et al.* Surface Characterization of Colloidal Silica Nanoparticles by Second Harmonic Scattering: Quantifying the Surface Potential and Interfacial Water Order. *The Journal of Physical Chemistry C* **123**, 20393-20404, https://doi.org/10.1021/acs.jpcc.9b05482 (2019).
2 Nony, L. in *2nd German-French summer school on noncontact-AFM* (Osnabrück, Germany, 2016).
3 Mazouzi, Y. *et al.* Biosensing Extracellular Vesicle Subpopulations in Neurodegenerative Disease Conditions. *ACS Sensors* **7**, 1657-1665, https://doi.org/10.1021/acssensors.1c02658 (2022).
