# Supplementary material for: Direct measurement of protein dipoles reveals the mechanism for like-charge adsorption on silica
Source: Nat Commun. 2026 Sep 15;17:10860. doi: 10.1038/s41467-026-76501-8 (PMC13672354; doi:10.1038/s41467-026-76501-8)
Supplement: Supplementary file 2 [file 41467_2026_76501_MOESM2_ESM.pdf]

nature portfolio

Peer Review File

Direct Measurement of Protein Dipoles Reveals The Mechanism For Like-Charge Adsorption on Silica

Corresponding Author: Professor Olivier Pluchery

This file contains all reviewer reports in order by version, followed by all author rebuttals in order by version.

Attachments originally included by the reviewers as part of their assessment can be found at the end of this file.

Version 0:

Reviewer comments:

Reviewer #1

(Remarks to the Author)

The manuscript authored by Lechaptois et al. is a KPFM study on Ferritin molecules adsorbed to silica surfaces. The authors have applied a number of techniques to the problem of Ferritin adsoprtion to silica: FTIR spectroscopy, AFM, KPFM, QCM-D and LSPR. Overall the study appears to be an attempt to leverage inferences from KPFM and relate them in some form to the like-charge attraction problem. Both the KPFM aspect and the attempt to shed light on like-charge attraction are in principle interesting and important. However there are several quantitative aspects of the study that are less clear, appear a bit disconnected and speculative, ultimately raising more questions than appear to be answered. Specifically, it is not clear what important mechanistic understanding actually emerges from this study.

Figs. 1 and 2 furnish evidence that Ferritin which is net negatively charged adsorbs to negatively charged silica surfaces. In order to clearly make the case at the outset for a counterintuitive/anomalous mechanism at work in the protein-surface interaction, it maybe useful to present in fig. 1 a few surface electrostatic potential or charge maps of Ferritin at a few pH values of interest (an example is shown in Fig. S8). An overall conceptual schematic including a negative silica surface would present the background of the case for the like-charge attraction problem that the authors claim to investigate in the title of the manuscript.

Figs. 3-4 threw me off a bit for many reasons. If the Ferritin 24-mer is indeed almost uniformly negatively charged as shown in Fig. S8, it is not entirely clear from where the measured molecular dipole stems or what role the dipole really plays in the problem. In the schematics of Fig. 6 the distribution of iron in the core of the assembly seems to be fairly isotropic. The surface charge appears to be isotropically negative. The experimental observation of protein adsorption to silica certainly suggests an attractive force between the like-charged ferritin and the silica surface, causing deposition and monolayer formation as shown in Figs. 1 and 2. But how does the dipole measured using KPFM add any further understanding than what is seen already in Figs 1 and 2? In fact it is not immediately obvious that there should be a net dipole arising the from the arrangement of charge as shown. Is it possible that the net dipole being reported on arises from more microscopic structural sources? How does the symmetry of the isotropic charge distribution in the 24-mer Ferritin get broken by iron ions to generate a dipole of the kind shown? Is it possible that the mechanistic origin of the Ferritin dipole shown is rather an interpretation? -- in which case that would need to be made very clear. On that note, how reliable are the reported inferences on dipole moment orientation? Is there a control experiment that could be done where the dipole moment undergoes an inversion and the CPD displays the expected modulation? Are there other protein systems that show an inverted dipole orientation? That said it is not clear at all how and whether knowledge that this dipole may exist contributes to an enhanced understanding of the mechanism of the interaction between the protein and the surface.

A couple other conceptual points: The authors refer to Lyklema and suggest that KPFM may be able to probe the potential drop between an interface and the bulk solution. Ref. 35 was specifically referring to interfaces in a solvent. Any method which relies on a measurement with respect to a reference surface/interface - which is what KPFM offers to my understanding - will by definition not be able to probe the required absolute potential difference between the surface and the bulk solvent/electrolyte. I suggest removing this comment as it is misleading. I might further dissuade the authors from using

non-standard anthropomorphic terminology such as "hostile adsorption". If adsorption occurs then there is nothing "hostile" about the process per se, but it is certainly counterintuitive, if that's what the authors mean.

The authors conclude by saying their observations "do not conflict with DLVO theory". It is not clear what is meant by this statement as the electrostatic part of DLVO theory rules out attraction between like-charged objects. Further, any short range attraction between a dipole and a charged surface would be expected to orient the dipole in the opposite orientation to that measured as shown in Fig. 6a. The experimentally reported configuration shown in Fig. 6b would not be easily rationalizable within DLVO theory.

The conclusion of the study speculates that the protein undergoes reorganization that generates an attraction to a like charged surface. The outcome of such reorganization would still constitute a counterintuitive result, since the negative part of the protein appears to be attracted to the negative surface if the reported KPFM result is taken at face value. But this claim may then further suggest that the measured net dipole on the surface would not exist in Ferritin molecules in free solution, or indeed for Ferritin adsorbed to a chemically different/uncharged surface. Can this be shown? If so, it could provide some evidence for a statement of this nature that otherwise appears purely speculative.

Overall, I think the study attempts to address an important question. But the assemblage of results presented unfortunately come across as rather disjointed, and fails to provide a coherent picture or mechanism that advances the understanding of the underlying interaction. A key reason behind the sense of disjointedness created by the various bits of the study is that the observation of adsorption points to like-charge attraction in and of itself. Understanding of the mechanism behind this observation however is not enhanced very much by the measurement of the orientation of the molecular dipole by KPFM (granted the KPFM result on proteins may well be an important technical advance on its own?). Are the authors suggesting a major role in the molecule-surface interaction for the overall molecular dipole? If this is the case an in-depth KPFM study would need to be performed that shows how the adsorption is modulated by the magnitude and orientation of this dipole, fleshing this point out sufficiently. If however, the intention of the authors was to showcase the KPFM result as an achievement in itself then perhaps it would be better to ensure that the message of the manuscript is clear on this, and limited to the technical front, and only mention in discussion in the text how they believe the interaction is related to the like charge attraction problem. As such the title and abstract raise expectations that the manuscript does not successfully meet.

Reviewer #2

(Remarks to the Author)
In this manuscript, the authors studied ferritin adsorption on silica under different iron intakes with the Kelvin probe force microscopy. From the surface potential measurement, the authors presented their discoveries: (1) negative end side of the protein particle faces the negative charged surface of the substrate; (2) adsorption on surface significantly increases the dipoles of the protein; intake of the irons makes the ferritin rigid—which may cause charge redistribution. These discoveries, if convincingly validated, are important and worth attentions of the research community.

The manuscript is elegantly written, the logic is well thought with only a few glitches noted below.
However, since the discovery components of the manuscript are important, here I provide my criticism, in order to improve it and increase the credibility of the work.

A research problem. The discussion of “hostile surface”, and illustration is based on individual protein. However, the useful data is from monolayers. The issue is, in monolayer formation, the neighboring dipoles usually influence each other, i.e., induce the in-plane orientation alignment. This influence is not discussed. The individual measurement of the protein particle, of course, is more meaningful, if the resolution of KPFM could be high enough and less noisy (the current KPFM images of the individual particle (Figure 3b) was very streaky). A recent progress of KPFM is the pulsed force KPFM (The Journal of Physical Chemistry C, 128, 24, 9813), which delivers about ~10 nm spatial resolution under ambient conditions. The author could ask Bruker to implement this mode, I believe. The sub-par KPFM research tool that the authors were using was holding back their discovery.

The authors did not consider the small counter irons from the solution in the adsorption of the negatively charged protein to the negatively charged silica surface. The sample were created by drop cast, which means that there could be small positive ions in the solution that acts as a “glue” to bind two negatively charged surface. The authors did not discuss the such a possibility, or why it is not the case in the studies.

In the manuscript, the authors used “iron” to refer to the intake. I would say it is better to distinguish if it is Iron (elemental form), Fe_2O_3, or Fe_3O_4, or Fe^{3+}. To refer to the form of Iron is important, because they could have very different effects. For example, the ion form (Fe^{3+}) can move and change the dipole moment and polarizability. Fe_2O_3/Fe_3O_4 particles are solid, which actually contribute to the change of the rigidity with the iron intake. This aspect of discussion is not mentioned in this work. Discussion on the form of the iron inside ferritin would be very helpful for the readers to understand the presented phenomenon.

Line 53, the author needs to expand what is the DLVO theory, give it a brief description. The manuscript will be more self-contained.

Line 248. The estimation of the spatial resolution is not convincing. ~30 nm seems to be optimistic. A typical resolution for FM-KPFM or PeakForce KPFM is about 50 nm. A benchmark measurement is needed. The authors can perform such

measurements with Brukers' calibration sample. A calibration sample would also help understanding the relationship between the change of the measured surface potential values with the dipole orientation of the proteins. (so that to make sure there was no mix up on determining the sign of the charge/orientation of the surface potential. This is important.)

Figure 1c. The AFM image shows the tip is contaminated (or something sticky to the tip). The particles have the same orientation (tilted at ~30 degree from the vertical axis of the frame), which is highly unlikely under natural conditions.

Figure 3c. The schematic of Figure 3c does not match the caption.

Figure 4a. The figure panel labels "Si" and the caption is "Silica". Silica is SiO_2, not Si (Silicon).
The title has an acronym KPFM, it should be fully spelled, as KPFM is not yet a common word.

Reviewer #3

(Remarks to the Author)
Firstly, the adsorption of charged species, such as proteins, liposomes, or other species with complex surface charge distributions, on like-charged surfaces is neither new nor particularly mysterious. In fact, in the case of liposomes, the effects of varying the internal charge distributions of the constituent lipids has been investigated with inverse phosphocholine lipids. Qualitatively, the effects are explained by charge fluctuations and counterion effects, as well as local, vs. average, surface charge distributions. What is the evidence that a permanent dipole moment plays a role?

Secondly, the authors claim in line 70, that "Proteins are not orientationally symmetric". Ferritin, in particular, exhibits 432 symmetry with 24 symmetry elements (Harrison PM, Arosio P. The ferritins: molecular properties, iron storage function and cellular regulation. Biochim Biophys Acta. 1996 Jul 31;1275(3):161-203. doi: 10.1016/0005-2728(96)00022-9). It is one of the most orientationally symmetric proteins found in nature (Goodsell DS, Olson AJ. Structural symmetry and protein function. Annu Rev Biophys Biomol Struct. 2000;29:105-53. doi: 10.1146/annurev.biophys.29.1.105).

Line 79: "Based on these considerations, we hypothesize that interface-induced changes in dipole moments of asymmetric proteins can provide a long-range attractive contribution to particle surface interaction and may overcome Coulombic repulsion." – ferritin is not "asymmetric".

Then, Figure 1a shows a dipole moment of ferritin, but there is no description of how that dipole moment was calculated or what its value is, nor of its relationship with the symmetry of ferritin.

Third, in line 113, the authors say that "… KPFM is capable of challenging J. Lyklema's assertion that surface potentials are part of the immeasurable quantities" – that would depend on the plane, at which the potential is measured, which was basically . Lyklema's point… At any rate, I am not sure what the relevance of this comment is.

To the data (Figure 5) and their interpretation:

The authors claim, in line 371, that "Since LSPR is a refractometric technique, the wavelength shift is induced by the change in the refractive index on the surface induced by protein adsorption. Therefore, the water molecules possibly trapped within the protein layer, especially when this layer is not rigid, have no impact on the response." - that is true, but the refractive index of the layer is a function of the iron content (Gupta R, Alamrani NA, Greenway GM, Pamme N, Goddard NJ. Method for Determining Average Iron Content of Ferritin by Measuring its Optical Dispersion. Anal Chem. 2019 Jun 4;91(11):7366-7372. doi: 10.1021/acs.analchem.9b01231; see, in particular, Figure 4 for the comparison between ferritin and apoferritin). Have the authors not tried to determine the adsorbed mass?

Also, I am not sure how the comparison between adsorption on the negative-charged silica and positive-charged APTES is relevant to the conclusions of the paper.

Finally, in line 377: "Finally, both QCM-D and LSPR show that the nature of the ferritin layer is dependent of the iron content. At low iron content, the formed layer is viscoelastic and highly hydrated while at high iron content the layer is rigid and compact, with a level of hydration twice lower. The data in Figure 5c suggest that the 2400 loading is the inflection point of this profound change in the ferritin layers properties where the internal structural change occurs." – This is a good example of how QCMD data is misinterpreted. In the case of adsorbed particles, QCMD does not provide information about the particles' viscoelastic properties, only their size and contact stiffness, which may, indeed, depend on the iron loading.

It would seem to me that what the authors are measuring is an asymmetry in the protein surface charge distribution induced by the adsorption to a charged surface and the counterion effects. What are the KPFM results on APTES surfaces? If the authors were to measure CPD maps on different surfaces, SiO2, gold, APTES SiO2 and gold, and relate that to properly measured adsorbed mass, protein amounts, etc., that would lead to an interesting picture of how protein surface charge distribution changes as a function of the properties of the surface to which the protein is adsorbing. It is indeed possible, even likely, that adsorption to a surface changes the charge distribution on the surface of the protein, and that those changes depend on the iron loading. It is also possible that there is a structural transition in the protein as a function of iron loading around the loading filling up the entire space available in the protein corona. However, to claim symmetry breaking due to the iron loading in terms of a dipole moment from the data that is presented is not convincing.

Minor comment: Line 336: what is "nanostructured quartz"? LSPR surfaces are silica-coated QCMD sensors, which themselves are gold-coated quartz.

Reviewer #4

(Remarks to the Author)
The paper deal with the protein's adhesion mechanisms on silica surface focusing on electric dipole contribution. The authors provided a multiscale experimental insight on ferritin adhesion on silica surface combining surface potential measurement at nanoscale (KPFM) and LSR/QCM. The developed experimental approached is interesting and the results provided in this study could have a great impact as the mechanism underlying the protein adhesion due to electrostatic interaction remain poorly understood. However, this paper suffers from a lot of issues which need to be addressed before publication.
1. According to the author this paper focus on ferritin adhesion on silica surface. Unfortunately, the investigated surface is poorly defined and characterized. As surface as a real impact on adhesion this part needs to be clarified.
• First of all, the authors argue that ferritin adhesion on silica is investigated. However, the surface investigated is confusion. Indeed, in experiment part only silicon is addressed. No information is provided about silica layer processing. In the present form, this imply silicon surface is native oxide. However, this surface is unsuitable to investigate adhesion as this is not stochiometric oxide and the properties are uncontrolled. Moreover, on Figure 4 silicon is indicated instead of silica (title). Please clarify the surface used.
• As adhesion on surface is addressed, the surface needs to be characterized accurately. Please provide information about silica thickness, roughness, hydrophobicity….
• Concerning surface properties, all measurements are done on "silica". However, QCM and LSR are performed on nanostructured quartz with APTES surface conditioning. This surface is really different to silica surface. So, these results are difficult to be compared previous one on silica. Please discuss this.
2. CDP measurement
• According to Metliz et al (ref. 56), when the DC bias is applied on the tip, VDC=VCDP=(WFsample-WPtip)/lql. Please correct the text page 14 (lines 553).
• Moreover, please indicate what configuration is used (as PF-KPFM is used, the DC should be applied on sample). This aspect is important to discuss the type of charge (electron/hole) in KPFM.
3. Spatial resolution for topography and KPFM measurement
• As protein dimension is small (8-12nm), please justify the fact to performed measurement with large curvature radius tip (Ptlr coated tip) instead for low curvature radius one (doped Si for example). This has a strong impact on result accuracy and performance.
o Concerning surface topography measurement on isolated ferritin particles. The authors argue that ferritin lateral width is constant whatever the Fe content (lines 180 pages 4). However, surface topography profiles (figure 2c) show clearly an increasing of the FWHM from 30nm (600Fe) to 60nm (4800Fe). Moreover, this width increasing is depicted on summary scheme (Figure 6d). Please discuss and clarify this point.
o Concerning surface topography measurement on monolayer, Figure S6 demonstrates that films are not continuous and these results permit to extract surface coverage. Please discuss the impact of poor lateral resolution of surface topography, on surface coverage determination.
• Concerning CDP measurement, the contrast could occur from material difference and/or trapped charges. Pages (lines 214-216), the negative CDP is attributed to to charge distribution. Please justify this assumption. Indeed, this negative CDP could be attributed by proteins themselves instead of charge.
• The part dedicated to CDP measurement on isolated ferritin nanoparticle, is less convincing. Indeed, as error bars have the same order of magnitude as CDP variation with Fe loading, the trend is not convincing. Moreover, the lateral resolution is impacted by parasitic capacitance and topography lateral resolution (as measurements are performed in lift mode). The evolution in figure 3e could be related to Ferritin FWHM evolution (and related decreasing of the silica contribution to parasitic capacitance) on CDP. Please discuss this part.
• Quite the same effect could explain for ferritin layer (figure 4). As the ferritin layer is not continuous (mainly for low Fe loading), the ferritin CDP could be impact by substrate CDP. Please discuss this.
• On figure 4d, the CDP profiles present a large transition area (around 1µm) at Si/Ferritin interface. Moreover, for 4800Fe a negative peak is observed (variation of 25mV compare to ferritin). Please discuss these.
4. All measurements preformed by KPFM are done on dry surface instead of ferritin adhesion occurred in liquid media. In liquid, ions could have an impact on electrostatic interaction and ferritin dipolar moment is impact by the media (liquid VS dry air). Please discuss the validity of the transposition of results obtained on dry media to phenomenon occurring in liquid one.
5. Minor remarks
• Page 3: please justify the fact that a ferritin monolayer is obtained. On figure 1c, white dots present height of around 18nm which could correspond to a ferritin nanoparticle on the top of layer. Please discuss this.
• QCM and LSR measurements are performed in dynamic mode. Please justify this choice. Indeed, adhesion under shear stress flow undergoes different mechanisms than static condition. Please indicate the flow and related pressure.
• Figure 5, please used the same parameter for figures and caption (K, F…)

Version 1:

Reviewer comments:

Reviewer #2

(Remarks to the Author)
The majority of my concerns have been addressed. The manuscript is much improved with the updated figures and expansion of discussions. It is easier to read now compared with previous version. The work is close to the level of recommendation of publication.

Reviewer #4

(Remarks to the Author)
First of all, I want to thank the authors to addressed all remarks with relevant information. I just want to come back on remarks [28] concerning the sign of the CDP related to Melitz et al paper. I apologize if my remark was not clearly explained. I agree with the definition of the CPD: VCPD= (WFsample-WFtip)/|q|. However, I do not agree with the relation between VDC and VCPD. According to Metliz et al., a DC bias applied on sample implies, to null the electrostatic force, that VDC = - VCPD (equation 2.8).
This point is extremely important as the CDP sign impacts charges sign and consequently all the discussion in the paper. In this case, two options are identified: (i) the CDP discuss in all the paper correspond to – VDC obtained in KPFM measurement and all the discussion is OK or (ii) the CDP correspond to VDC obtained by KPFM and consequently the sign is false and the discussion is impacted (probed charges are no more negative but positive) and the paper need to be rewritten.
Please check this issue.
Consequently, I consider that the paper can’t be published in the present form.

Reviewer #5

(Remarks to the Author)
See the attached file (Ferritin.pdf)

Reviewer #6

(Remarks to the Author)
Task: This work already went thorough one round of peer review with us and the authors have revised the study according to reviewers' comments. Reviewer 1 is unfortunately unable to review. We therefore ask if you could assess authors' responses to Reviewer 1, and let us know if they are sufficiently addressed. We do not require a full, new report, but an assessment of responses to Reviewer 1 only.

I believe the authors have largely addressed the questions raised by Reviewer #1; however, I am raising concerns regarding the significance of the study and the clarity of the central message the authors are trying to communicate.
The manuscript prepared by Lechaptois et al. investigated ferritin adsorption on silica and framed this phenomenon as a form of like-charge attraction mediated by cationic “electrostatic bridges.” However, prior work by Krishnan et al. specifically demonstrated like-charge attraction as a solvent-mediated, long-range effect between negatively charged particles in water, and further showed that the addition of salt (e.g., NaCl) weakens the attraction due to screening instead of strengthening it. Rather, the study done by the authors demonstrates clearly that the observed adsorption is a counterion mediated effect, which is already well established. And it is not entirely clear how the measured dipole moment plays a role in the adsorption.
While the experimental approach has been carefully re-examined and the KPFM measurements are technically interesting, the manuscript does not convincingly demonstrate a new mechanism of like-charge attraction beyond established counterion-mediated adsorption. Moreover, it remains unclear how the measured dipole moment contributes mechanistically to the adsorption process, as the dipole appears more likely to be a consequence of ion-mediated adsorption rather than a driving force.
In summary, I believe the significance of the manuscript lies more in the characterization of the adsorbed state of ferritin than in advancing the fundamental understanding of interactions between like-charged particles.

Specific comments following Reviewer #1’s questions:
[1] /[3] Have the authors attempted measurements at different pH values? Figure 1 naturally raises question about how the adsorption state and the measured dipole moment would change with pH. While I understand the authors might prefer to focus on physiological conditions, control experiments at around pH 2 (both ferritin and silica being positively charged) and pH 4 (ferritin being positive while silica being negative) would aid to the robustness of the CPD measurements following Reviewer #1’s request.
[5] DLVO theory describes colloidal stability and interaction potentials of particles in solution. It is therefore unclear how this study is directly relevant or transferable to the dry-state measurements performed by KPFM.
[6] One concerning point for me is the use of variable deposition times and the drying process of the buffer solution. How do the authors actively control the final salt concentration since it can increase substantially in the drying (“blowing away”) process, such that ferritin is simply being “salted out”?
Also, it would be helpful to clarify what is expected during the transient drying stage in terms of the state of protein adsorption and the dipole moment. How representative is the KPFM result measured at the air-silica interface compared to water-silica interface in solution?
[7] While the experimental work is careful and technically interesting, I do agree with Reviewer #1 that the central message of the manuscript is not yet sufficiently clear.

Version 2:

Reviewer comments:

Reviewer #4

(Remarks to the Author)
The reviewer wants to thank these authors to address all remarks and improve the manuscript. I think that manuscript could be published in the present form.

Reviewer #5

(Remarks to the Author)
I am not convinced that the layers are dense films
The QCM part is below that current state of the art in my view

Reviewer #6

(Remarks to the Author)
The authors have addressed my previous concerns to some extent. While the experimental methods are technically rigorous and novel, the biological mechanistic claims appear slightly overstated. The dipole measurements are performed in a dry, post-adsorption state, which limits the extent to which the results can be interpreted in the context of adsorption mechanisms in aqueous environments.

Overall, I do not believe the manuscript is ready for recommendation to Nature Communications.

Version 3:

Reviewer comments:

Reviewer #5

(Remarks to the Author)
The authors have shortened the QCM part. That was a step into the right direction.
The paper can be published from my side

Reviewer #6

(Remarks to the Author)
I am still not convinced that the dipole measurements obtained in the dry, post-adsorption state can be directly translated to interfacial behaviour in an aqueous environment. While the authors acknowledge this limitation in the rebuttal, it is not clearly stated or sufficiently discussed in the manuscript itself, which I find potentially misleading. In addition, the manuscript does not explain how the surface charge state of the silica substrate evolves when transitioning from the aqueous environment to the dry measurement conditions, further limiting the interpretation of the results.

Overall, I do not see substantial improvement in addressing this key concern or in openly discussing it in the manuscript, and I do not support publication of the manuscript in Nature Communications.

Open Access This Peer Review File is licensed under a Creative Commons Attribution 4.0 International License, which permits use, sharing, adaptation, distribution and reproduction in any medium or format, as long as you give appropriate credit to the original author(s) and the source, provide a link to the Creative Commons license, and indicate if changes were made.
In cases where reviewers are anonymous, credit should be given to 'Anonymous Referee' and the source.
The images or other third party material in this Peer Review File are included in the article's Creative Commons license, unless indicated otherwise in a credit line to the material. If material is not included in the article's Creative Commons license and your intended use is not permitted by statutory regulation or exceeds the permitted use, you will need to obtain permission directly from the copyright holder.
To view a copy of this license, visit https://creativecommons.org/licenses/by/4.0/

Response to the reviewers

Report received on 19-Dec-2024

Dear Reviewers,

We are thankful for the numerous and relevant comments made by the four reviewers. We have done our best to improve our manuscript according to these comments and we have performed a large set of complementary experiments including new QCM-D, new AFM with sharper tip and better resolution, and new KPFM measured with new experimental conditions. We have added eight new figures, mostly in the Supplementary Information. At the end, our understanding has deepened and we think our overall study grew stronger.

Changes in the manuscript are highlighted.

Please find below the reviewer's comments along with our point-by-point responses. We have added numbers and titles to the reviewer's comments.

The authors.
30-April-2025

Reviewer #1 (Remarks to the Author):

The manuscript authored by Lechaptois et al. is a KPFM study on Ferritin molecules adsorbed to silica surfaces. The authors have applied a number of techniques to the problem of Ferritin adsoprtion to silica: FTIR spectroscopy, AFM, KPFM, QCM-D and LSPR. Overall the study appears to be an attempt to leverage inferences from KPFM and relate them in some form to the like-charge attraction problem. Both the KPFM aspect and the attempt to shed light on like-charge attraction are in principle interesting and important. However there are several quantitative aspects of the study that are less clear, appear a bit disconnected and speculative, ultimately raising more questions than appear to be answered. Specifically, it is not clear what important mechanistic understanding actually emerges from this study.

[1] a clear schematic in Fig1
Figs. 1 and 2 furnish evidence that Ferritin which is net negatively charged adsorbs to negatively charged silica surfaces. In order to clearly make the case at the outset for a counterintuitive/anomalous mechanism at work in the protein-surface interaction, it maybe useful to present in fig. 1 a few surface electrostatic potential or charge maps of Ferritin at a few pH values of interest (an example is shown in Fig. S8). An overall conceptual schematic including a negative silica surface would present the background of the case for the like-charge attraction problem that the authors claim to investigate in the title of the manuscript.

Indeed, we have added a scheme in Fig 1-a to clarify the situation of charged surface and charged ferritin.

[2] Resulting dipole and counter-ions cf. [10, 16, 23]
Figs. 3-4 threw me off a bit for many reasons. If the Ferritin 24-mer is indeed almost uniformly negatively charged as shown in Fig. S8, it is not entirely clear from where the measured molecular

dipole stems or what role the dipole really plays in the problem. In the schematics of Fig. 6 the distribution of iron in the core of the assembly seems to be fairly isotropic. The surface charge appears to be isotropically negative. The experimental observation of protein adsorption to silica certainly suggests an attractive force between the like-charged ferritin and the silica surface, causing deposition and monolayer formation as shown in Figs. 1 and 2. But how does the dipole measured using KPFM add any further understanding than what is seen already in Figs 1 and 2? In fact it is not immediately obvious that there should be a net dipole arising the from the arrangement of charge as shown. Is it possible that the net dipole being reported on arises from more microscopic structural sources? How does the symmetry of the isotropic charge distribution in the 24-mer Ferritin get broken by iron ions to generate a dipole of the kind shown? Is it possible that the mechanistic origin of the Ferritin dipole shown is rather an interpretation? -- in which case that would need to be made very clear.

We thoroughly worked this question in the revised manuscript. Indeed, we modified Fig 3c to represent more scientifically the direct results from our KPFM data prior to any interpretation: the negative variation of the CPD for each Ftn we have measured means that each adsorbed ferritin results in an outward dipole. We agree that this adsorbed species might also include counter ions such as Na^+. This negative value corresponds to an outward dipole. This is an experimental result that we use as starting point for the interpretation and discussion. We have added the following sentences page 6:

> *These systematic negative values of the CPD are indicative of a specific charge distribution induced by the ferritins, where the negative charges accumulate closer to the surface and the positive ones further away as illustrated in Fig 3c-c. This outward dipole moment is an unexpected result and will be discussed in detail.*

[3] Supplemental KPFM control experiments?

On that note, how reliable are the reported inferences on dipole moment orientation? Is there a control experiment that could be done where the dipole moment undergoes an inversion and the CPD displays the expected modulation? Are there other protein systems that show an inverted dipole orientation? That said it is not clear at all how and whether knowledge that this dipole may exist contributes to an enhanced understanding of the mechanism of the interaction between the protein and the surface.

The relationship between CPD changes and the dipole moment orientation is discussed in the references indicated in our text: Ref 49 (Heimel 2011); Ref 50 (Asyuda 2020) and Ref 51 (Bossard-Giannesini 2023)

In order to strengthen the interpretation of the CPD values, we have also added a figure S2 and the following sentence below page 8. This proves that our CPD values have the correct sign (see also comment [13] below.

> *In Figure S2, we show how KPFM is sensitive to the WF of the substrate materials, with CPD values evolving from −1100 mV, −430 mV and +0.030 mV when scanning through alumina, silica and gold, respectively*

[4] Our comments on Lyklema anthropomorphic wording. Cf [19]

A couple other conceptual points: The authors refer to Lyklema and suggest that KPFM may be able to probe the potential drop between an interface and the bulk solution. Ref. 35 was specifically referring to interfaces in a solvent. Any method which relies on a measurement with respect to a reference surface/interface - which is what KPFM offers to my understanding - will by definition not be able to probe the required absolute potential difference between the surface and the bulk solvent/electrolyte. I suggest removing this comment as it is misleading. I might further dissuade the authors from using non-standard anthropomorphic terminology such as "hostile adsorption". If adsorption occurs then there is nothing "hostile" about the process per se, but it is certainly counterintuitive, if that's what the authors mean.

We have removed this mention of Lyklema on line 113, since indeed this was refereeing to protein in liquid.
We have also replaced the wording “hostile” with more scientific wording in 5 places in the manuscript

[5] DLVO theory. Cf [12]
The authors conclude by saying their observations "do not conflict with DLVO theory". It is not clear what is meant by this statement as the electrostatic part of DLVO theory rules out attraction between like-charged objects. Further, any short range attraction between a dipole and a charged surface would be expected to orient the dipole in the opposite orientation to that measured as shown in Fig. 6a. The experimentally reported configuration shown in Fig. 6b would not be easily rationalizable within DLVO theory.

We have clarified the conclusion regarding DLVO theory, it reads :

> *The interaction of proteins with surfaces is typically described by the Derjaguin–Landau–Verwey–Overbeek (DLVO) theory, which was initially developed to explain the kinetic stability of colloidal dispersions and later extended to encompass interactions between charged species in general.[16,17] In DLVO theory, repulsive electrostatic forces between like-charged entities in aqueous media prevent their close approach and interaction. When sufficiently strong, these repulsive forces confer colloidal stability by inhibiting aggregation or adsorption.*

[6] control KPFM experiment
The conclusion of the study speculates that the protein undergoes reorganization that generates an attraction to a like charged surface. The outcome of such reorganization would still constitute a counterintuitive result, since the negative part of the protein appears to be attracted to the negative surface if the reported KPFM result is taken at face value. But this claim may then further suggest that the measured net dipole on the surface would not exist in Ferritin molecules in free solution, or indeed for Ferritin adsorbed to a chemically different/uncharged surface. Can this be shown? If so, it could provide some evidence for a statement of this nature that otherwise appears purely speculative.

Your remark has pushed us to perform several complementary experiments and unveil the role of the charged sodium cations as electrostatic bridge between the silica surface and the ferritin:

- *we have measured the presence of Na+ physisorbed on the surface when there is no rinsing with MiliQ water. See Section 8 in the SI*
- *We have performed an adsorption test of Ftn as a function of NaCl concentration, that we have characterized with AFM (section 9 of the SI) and QCM-D performed in the stop-flow configuration (section 10 of the SI).*

[7] Reorient the global objective of the article?
Overall, I think the study attempts to address an important question. But the assemblage of results presented unfortunately come across as rather disjointed, and fails to provide a coherent picture or mechanism that advances the understanding of the underlying interaction. A key reason behind the sense of disjointedness created by the various bits of the study is that the observation of adsorption points to like-charge attraction in and of itself. Understanding of the mechanism behind this observation however is not enhanced very much by the measurement of the orientation of the molecular dipole by KPFM (granted the KPFM result on proteins may well be an important technical advance on its own?). Are the authors suggesting a major role in the molecule-surface interaction for the overall molecular dipole? If this is the case an in-depth KPFM study would need to be performed

that shows how the adsorption is modulated by the magnitude and orientation of this dipole, fleshing this point out sufficiently. If however, the intention of the authors was to showcase the KPFM result as an achievement in itself then perhaps it would be better to ensure that the message of the manuscript is clear on this, and limited to the technical front, and only mention in discussion in the text how they believe the interaction is related to the like charge attraction problem. As such the title and abstract raise expectations that the manuscript does not successfully meet.

We took your comment into consideration and reorient our article

Reviewer #2 (Remarks to the Author):

In this manuscript, the authors studied ferritin adsorption on silica under different iron intakes with the Kelvin probe force microscopy. From the surface potential measurement, the authors presented their discoveries: (1) negative end side of the protein particle faces the negative charged surface of the substrate; (2) adsorption on surface significantly increases the dipoles of the protein; intake of the irons makes the ferritin rigid—which may cause charge redistribution. These discoveries, if convincingly validated, are important and worth attentions of the research community.

The manuscript is elegantly written, the logic is well thought with only a few glitches noted below. However, since the discovery components of the manuscript are important, here I provide my criticism, in order to improve it and increase the credibility of the work.

[8] in monolayer, in-plane dipole mutual influence ?
A research problem. The discussion of "hostile surface", and illustration is based on individual protein. However, the useful data is from monolayers. The issue is, in monolayer formation, the neighboring dipoles usually influence each other, i.e., induce the in-plane orientation alignment. This influence is not discussed. The individual measurement of the protein particle, of course, is more meaningful, if the resolution of KPFM could be high enough and less noisy (the current KPFM images of the individual particle (Figure 3b) was very streaky).

Thanks for your comment.
First, in the revised article we have modified Fig 1-c and replace the AFM image taken with the KPFM tip with an AFM recorded with a sharper tip. The image is much less noisy. It shows that the ferritins are not so close-packed.
Second, we measured that our "monolayers" are made of ~450 Ftn/µm². Whereas a close-packed layer would contain 2000 Ftn/µm².
Finally, for these reasons, we have decided not to tackle this question of the mutual dipole-dipole interaction.

[9] High-res pulsed KPFM
A recent progress of KPFM is the pulsed force KPFM (The Journal of Physical Chemistry C, 128, 24, 9813), which delivers about ~10 nm spatial resolution under ambient conditions. The author could ask Bruker to implement this mode, I believe. The sub-par KPFM research tool that the authors were using was holding back their discovery.

Thanks a lot for pointing this recent result. We are considering using this approach for further studies. But for the present result, and using the approach with the monolayer we believe we had overcome our issue of lateral resolution and we obtained reliable values of the dipole moment.

[10] Counter-ions cf. [2, 16, 23]

The authors did not consider the small counter irons from the solution in the adsorption of the negatively charged protein to the negatively charged silica surface. The sample were created by drop cast, which means that there could be small positive ions in the solution that acts as a “glue” to bind two negatively charged surface. The authors did not discuss the such a possibility, or why it is not the case in the studies.

We must really thank the reviewer for this comment which pushes us carrying out complementary experiments for unraveling the role of the counterions and namely the sodium cations.
We have considerably developed our text to include the role played by this cation. We have added three sections in the S.I. with additional experiments to explain the role of Na+: see sections 8, 9 and 10.

[11] Fe2O3? Fe3O4? in the Ftn?
In the manuscript, the authors used “iron” to refer to the intake. I would say it is better to distinguish if it is Iron (elemental form), Fe2O3, or Fe3O4, or Fe^3+. To refer to the form of Iron is important, because they could have very different effects. For example, the ion form (Fe^3+) can move and change the dipole moment and polarizability. Fe2O3/Fe3O4 particles are solid, which actually contribute to the change of the rigidity with the iron intake. This aspect of discussion is not mentioned in this work. Discussion on the form of the iron inside ferritin would be very helpful for the readers to understand the presented phenomenon.

The iron core inside ferritin has been long studied by different groups in the field. Depending on the amount of iron loading and the loading method, the composition in the core varies. Our recent work contributes to understanding of the iron core in the specific *Archaeoglobus ferritin* in this study [https://doi.org/10.1016/j.msec.2021.112282]. Multiple nucleation sites have been identified within the ferritin which contributes to the growth of the Fe_xO_y crystals in the core. As the reviewer mentions, as the crystal grows, the core fills-up resulting in the change in rigidity.

The text has been improved to better reflect the composition of the iron core as follows:

Discussion
“The nucleation occurs at multiple sites in the core and grows into the lumen of the cavity forming crystals of iron oxide nanoparticles.[59] The formation of iron oxide nanoparticles explains the resulting in the change of the viscoelastic property of the ferritin. »

And,

“ Due to the orientation of the protein on the surface, and since the bond is likely to be formed through the 4-fold axis (positive residues), the protein is arranged so the three other 4-fold axes point outwards as represented in Figure 6d. The negatively charged residues may have interacted with the sodium ions in the buffer imposing additional dipole moment. Furthermore, the presence of both iron(II) and iron(III) in the core of AfFtn-AA provide mobile elements that contribute to the change of dipole in the core.

Method

Loading of 4800 molar ratio of iron(II) into AfFtn-AA results in different composition of positively charged iron(II) and iron(III) depending on the method.[59] Loading at room temperature with aerobic condition results in higher iron(II) to iron(III) ratio (60:40) while at high temperature (i.e., 65°C) with anaerobic condition the ratio of iron(II) to iron(III) is

relatively equal (55:45). In this study, iron has been loaded at room temperature with aerobic condition and we expect to have higher iron(II) composition compared to iron(III). Furthermore, multiple nucleation sites have been identified in AfFtn-AA. As more iron(II) is converted into iron(III) and nucleate, iron oxide particles form and the core fills up resulting in potentially more rigid structures that is more resistant to deformation.[43]

[12] DLVO theory cf. [5]
Line 53, the author needs to expand what is the DLVO theory, give it a brief description. The manuscript will be more self-contained.

The following explanation was added in the introduction. It now reads:

The interaction of proteins with surfaces is typically described by the Derjaguin–Landau–Verwey–Overbeek (DLVO) theory, which was initially developed to explain the kinetic stability of colloidal dispersions and later extended to encompass interactions between charged species in general.16,17 In DLVO theory, repulsive electrostatic forces between like-charged entities in aqueous media prevent their close approach and interaction. When sufficiently strong, these repulsive forces confer colloidal stability by inhibiting aggregation or adsorption

[13] KPFM control experiments on sign changes for CPD
Line 248. The estimation of the spatial resolution is not convincing. ~30 nm seems to be optimistic. A typical resolution for FM-KPFM or PeakForce KPFM is about 50 nm. A benchmark measurement is needed. The authors can perform such measurements with Brukers' calibration sample. A calibration sample would also help understanding the relationship between the change of the measured surface potential values with the dipole orientation of the proteins. (so that to make sure there was no mix up on determining the sign of the charge/orientation of the surface potential. This is important.)

We have added the AFM/KPFM images recorded on the Bruker calibration sample in Figure S2 with detailed explanations. Indeed, we systematically performed a check of the values before and after each of our KPFM session in order to verify that our tip was not modified or contaminated during the scans. We confirm that we did not mix the signs for the CPD.

[14] KPFM tip contaminated
Figure 1c. The AFM image shows the tip is contaminated (or something sticky to the tip). The particles have the same orientation (tilted at ~30 degree from the vertical axis of the frame), which is highly unlikely under natural conditions.

Indeed, the image in Figure 1c was extracted from a 5x5 μm^2 image recorded with a SCM-PITv2 tip and could explain the artefact that appears on the surface.
However, we modified the Figure 1-c with a new topography image of (Fe4800)AfFtn-AA on silica recorded with a sharper tip (2 nm radius), that displays better resolve ferritin particles on the surface without artefacts.

[15] to be corrected
Figure 3c. The schematic of Figure 3c does not match the caption.

The caption has been corrected

Figure 4a. The figure panel labels "Si" and the caption is "Silica". Silica is SiO_2, not Si (Silicon).
The title has an acronym KPFM, it should be fully spelled, as KPFM is not yet a common word.

“Si” label was replaced by “Silica” on the Figure 4.a

Reviewer #3 (Remarks to the Author):

[16] counter-ions cf. [2, 10, 23]

Firstly, the adsorption of charged species, such as proteins, liposomes, or other species with complex surface charge distributions, on like-charged surfaces is neither new nor particularly mysterious. In fact, in the case of liposomes, the effects of varying the internal charge distributions of the constituent lipids has been investigated with inverse phosphocholine lipids. Qualitatively, the effects are explained by charge fluctuations and counterion effects, as well as local, vs. average, surface charge distributions. What is the evidence that a permanent dipole moment plays a role?

Thanks for your comment. We considerably modified our manuscript. Now we include the essential role of the sodium cation for explaining the immobilization of negative protein on a negatively charged silica surface.

[17] is Ftn asymmetric?

Secondly, the authors claim in line 70, that “Proteins are not orientationally symmetric”. Ferritin, in particular, exhibits 432 symmetry with 24 symmetry elements (Harrison PM, Arosio P. The ferritins: molecular properties, iron storage function and cellular regulation. Biochim Biophys Acta. 1996 Jul 31;1275(3):161-203. doi: 10.1016/0005-2728(96)00022-9). It is one of the most orientationally symmetric proteins found in nature (Goodsell DS, Olson AJ. Structural symmetry and protein function. Annu Rev Biophys Biomol Struct. 2000;29:105-53. doi: 10.1146/annurev.biophys.29.1.105).

Line 79: “Based on these considerations, we hypothesize that interface-induced changes in dipole moments of asymmetric proteins can provide a long-range attractive contribution to particle surface interaction and may overcome Coulombic repulsion.” – ferritin is not “asymmetric”.

The authors have meant to distinguish the shapes of inorganic nanoparticles from those of the ferritin. For clarity the text in the introduction has been revised to

Unlike solid inorganic microparticles that typically assume spherical shapes, proteins – particularly those with multiple subunits and cage-like structures (e.g., ferritin, viral capsid) – have inherent structural symmetry but rarely a perfect sphere; instead they assume polyhedral shapes (e.g., cube, octahedron, dodecahedron, icosahedron).[22,23]

And,

The model ferritin is derived from the archaeon Archaeoglobus fulgidus (AfFtn; tetrahedron) and engineered to reduce iron release from the protein core (AfFtn-AA; octahedron).[29,30]

[18] Fig 1a = calculated dipole

Then, Figure 1a shows a dipole moment of ferritin, but there is no description of how that dipole moment was calculated or what its value is, nor of its relationship with the symmetry of ferritin.

The dipole moment of the free ferritin was calculated (79 D) and is taken from the Protein Dipole Moments Server established by Felder, Prilusky and Sussman. Our text states it clearly now :

These values are notably larger than the ferritin in solution whose dipole moment was calculated to be 79 D.[52]

[19] Lyklema cf. [4]

Third, in line 113, the authors say that "… KPFM is capable of challenging J. Lyklema's assertion that surface potentials are part of the immeasurable quantities" – that would depend on the plane, at which the potential is measured, which was basically . Lyklema's point… At any rate, I am not sure what the relevance of this comment is.

We have removed this mention of Lyklema on line 113, since indeed this was refereeing to protein in liquid.

[20] LSPR

To the data (Figure 5) and their interpretation:

The authors claim, in line 371, that "Since LSPR is a refractometric technique, the wavelength shift is induced by the change in the refractive index on the surface induced by protein adsorption. Therefore, the water molecules possibly trapped within the protein layer, especially when this layer is not rigid, have no impact on the response." - that is true, but the refractive index of the layer is a function of the iron content (Gupta R, Alamrani NA, Greenway GM, Pamme N, Goddard NJ. Method for Determining Average Iron Content of Ferritin by Measuring its Optical Dispersion. Anal Chem. 2019 Jun 4;91(11):7366-7372. doi: 10.1021/acs.analchem.9b01231; see, in particular, Figure 4 for the comparison between ferritin and apoferritin). Have the authors not tried to determine the adsorbed mass?

Thank you. Following your comments we have now calculated the acoustic and optical mass. The data is now added to the SI section

[21] Silica substrate and quartz-APTES substrates

Also, I am not sure how the comparison between adsorption on the negative-charged silica and positive-charged APTES is relevant to the conclusions of the paper.

We have added new experiments of QCM-D of the immobilization directly on the non-functionalized silica surface with stop-flow experiments. These new experiments confirm that the immobilization of ferritin is controlled by the presence of the Na+ counterions. Meanwhile the QCM-D experiments with the adsorption on the APTES-treated silica still serves for measuring the viscoelastic properties of the ferritin, and assess the rigidity increase.

We fully agree with the reviewer that the ideal comparison would have been with an identical surface. However, we encountered a technical challenge: when adsorbing ferritin under the QCM-D conditions coupled with LSPR, the experiments must be conducted under flow. Even at very low flow rates, the ferritin is displaced, preventing proper study of its interaction with the surface.

We attempted to overcome this limitation by stopping the flow as soon as ferritin reached the surface. The resulting QCM-D curve for the 4800Fe system has been added to the Supporting Information, Figure S13a. Unfortunately, stopping the pump significantly affects the dissipation signal, which is expected because changes in flow rate alter the quality of the resonance. As a result, dissipation data—and thus any analysis of differences in the viscoelastic properties of the adsorbed layers—are not exploitable under these conditions. Nevertheless, the frequency shifts recorded in these experiments, as reported in the table above, confirm that the amount of ferritin adsorbed on the surface is identical regardless of the iron content. These observations support the conclusions we drew regarding the

adsorbed ferritin layer. While we agree that the interface between ferritin and the surface likely changes, the behavior of the adsorbed layer remains comparable.

**QCM-D with flow stopped upon adsorption**	600 Fe	1200 Fe	2400 Fe	3600 Fe	4800 Fe
Frequency shifts (Hz)	-165	-165	-160	-140	-160

[22] QCM-D and viscoelastic values

Finally, in line 377: "Finally, both QCM-D and LSPR show that the nature of the ferritin layer is dependent of the iron content. At low iron content, the formed layer is viscoelastic and highly hydrated while at high iron content the layer is rigid and compact, with a level of hydration twice lower. The data in Figure 5c suggest that the 2400 loading is the inflection point of this profound change in the ferritin layers properties where the internal structural change occurs." – This is a good example of how QCMD data is misinterpreted. In the case of adsorbed particles, QCMD does not provide information about the particles' viscoelastic properties, only their size and contact stiffness, which may, indeed, depend on the iron loading.

A detailed explanation on how QCM-D can be used to assess the viscoelastic properties of the layer can be found in the following references:

- Cho, N. J., C. W. Frank, B. Kasemo, and F. Höök. "Quartz Crystal Microbalance with Dissipation Monitoring of Supported Lipid Bilayers on Various Substrates." *Nature Protocols* 5, no. 6 (2010): 1096-106. https://doi.org/10.1038/nprot.2010.65.
- Höök, F., B. Kasemo, T. Nylander, C. Fant, K. Sott, and H. Elwing. "Variations in Coupled Water, Viscoelastic Properties, and Film Thickness of a Mefp-1 Protein Film During Adsorption and Cross-Linking:: A Quartz Crystal Microbalance with Dissipation Monitoring, Ellipsometry, and Surface Plasmon Resonance Study." *Analytical Chemistry* 73, no. 24 (Dec 2001): 5796-804. https://doi.org/10.1021/ac0106501.
- Reimhult, E., C. Larsson, B. Kasemo, and F. Höök. "Simultaneous Surface Plasmon Resonance and Quartz Crystal Microbalance with Dissipation Monitoring Measurements of Biomolecular Adsorption Events Involving Structural Transformations and Variations in Coupled Water." *Analytical Chemistry* 76, no. 24 (Dec 2004): 7211-20. https://doi.org/10.1021/ac0492970..
- Rodahl, M., F. Hook, C. Fredriksson, C. A. Keller, A. Krozer, P. Brzezinski, M. Voinova, and B. Kasemo. "Simultaneous Frequency and Dissipation Factor Qcm Measurements of Biomolecular Adsorption and Cell Adhesion." *Faraday Discussions* 107 (1997): 229-46.

The simplified explanation on how the QCM-D set-up is utilized here allows measuring the visco-elastic properties of the layer can be found at the instrument supplier webpage: https://www.biolinscientific.com/measurements/qcm-d#information-you-can-extract-with-qcm-d
It states: The collected f and D at multiple harmonics allow for both qualitative information, such as revealing whether a molecule-surface interaction is taking place or not, as well as quantitative information on mass, thickness, and viscoelastic properties of the formed layer.

Therefore we are confident that our conclusions drawn from the QCM-D experiments are correct.

[23] counter-ions cf. [2, 10, 16]

It would seem to me that what the authors are measuring is an asymmetry in the protein surface charge distribution induced by the adsorption to a charged surface and the counterion effects. What are the KPFM results on APTES surfaces? If the authors were to measure CPD maps on different surfaces, SiO2, gold, APTES SiO2 and gold, and relate that to properly measured adsorbed mass, protein amounts, etc., that would lead to an interesting picture of how protein surface charge distribution changes as a function of the properties of the surface to which the protein is adsorbing. It is indeed possible, even likely, that adsorption to a surface changes the charge distribution on the surface of the protein, and that those changes depend on the iron loading. It is also possible that there is a structural transition in the protein as a function of iron loading around the loading filling up the entire space available in the protein corona. However, to claim symmetry breaking due to the iron loading in terms of a dipole moment from the data that is presented is not convincing.

> Following the reviewer comments, we have modified our text. In particular we now include a clear effect of the sodium counterions. I think we already answered to this comments in our answers to the previous reviewsers. See our response to comments [2], [10] and [16].

[24] Corrections

Minor comment: Line 336: what is “nanostructured quartz”? LSPR surfaces are silica-coated QCMD sensors, which themselves are gold-coated quartz.

> Thank you for this comment. While classical quartz crystals are indeed coated with a thin layer of gold, such substrates are not suitable for performing coupled QCM-LSPR measurements, as implemented in this study. In order to enable this coupling, the substrate must support a localized surface plasmon resonance (LSPR), which requires nanostructuring. For this reason, we employed nanostructured quartz substrates, where islands of nanogold are deposited to induce plasmonic activity and allow the detection of the LSPR band. These nanogold islands are subsequently coated with a thin silica layer.
>
> This was clarified in the methods section as follows:
> *Measurements were conducted using Acoulyte AT-cut quartz crystal sensors (Insplorion AB, Gothenburg, Sweden), which are QCM-D compatible substrates featuring randomly distributed gold nanodisks (100 nm diameter, 20 nm height) coated with a thin silica layer (5–10 nm). The interaction of light with these nanodisks induces a collective oscillation of conduction electrons—known as Localized Surface Plasmon Resonance (LSPR)—resulting in a pronounced absorption peak centered around 700 nm. Variations in the refractive index near the sensor surface lead to shifts in the LSPR peak position (denoted as Δλ_max), enabling real-time monitoring of biomolecular interactions. Frequency and dissipation changes were analyzed using QTools software (v3.1.33.567, Biolin Scientific, Sweden), while LSPR signal analysis was performed with Insplorer software (v1.3.1, Insplorion AB, Sweden).*

Reviewer #4 (Remarks to the Author):

The paper deal with the protein’s adhesion mechanisms on silica surface focusing on electric dipole contribution. The authors provided a multiscale experimental insight on ferritin adhesion on silica surface combining surface potential measurement at nanoscale (KPFM) and LSR/QCM. The developed experimental approached is interesting and the results provided in this study could have a great impact as the mechanism underlying the protein adhesion due to electrostatic interaction remain poorly understood. However, this paper suffers from a lot of issues which need to be addressed before publication.

1. According to the author this paper focus on ferritin adhesion on silica surface. Unfortunately, the investigated surface is poorly defined and characterized. As surface as a real impact on adhesion this part needs to be clarified.

Indeed, we have added a characterization of the initial surface: See section 1 of the SI

[25] Clarify Si, SiO2

• First of all, the authors argue that ferritin adhesion on silica is investigated. However, the surface investigated is confusion. Indeed, in experiment part only silicon is addressed. No information is provided about silica layer processing. In the present form, this imply silicon surface is native oxide. However, this surface is unsuitable to investigate adhesion as this is not stochiometric oxide and the properties are uncontrolled. Moreover, on Figure 4 silicon is indicated instead of silica (title). Please clarify the surface used.

Inded we are dealing with silica. “Si” has been replaced by “silica” on Figure 4-a, b and d

[26] Substrate characterization

As adhesion on surface is addressed, the surface needs to be characterized accurately. Please provide information about silica thickness, roughness, hydrophobicity….

The information was added in the **Methods** section. It now read:

***Silicon wafers** were used “as-received” in our experiments and the silica surfaces of this study corresponds to the 2 nm thick thermal oxide grown on the substrate. A careful cleaning was systematically carried out but without modifying the surface oxide.*

[27] Clarify Si, SiO2 cf. [25]

Concerning surface properties, all measurements are done on “silica”. However, QCM and LSR are performed on nanostructured quartz with APTES surface conditioning. This surface is really different to silica surface. So, these results are difficult to be compared previous one on silica. Please discuss this.

Thanks for your comment. Your question is similar to the comment [24] from Reviewer#3. We have modified our text accordingly (Methods section)
The nanostructured quartz used here are coated with a thin silica layer (5–10 nm). So the top surface is in both cases silica-terminated.

2. CDP measurement

[28] Sign convention in the KPFM

According to Metliz et al (ref. 56), when the DC bias is applied on the tip, VDC=VCDP=(WFsample-WPtip)/IqI. Please correct the text page 14 (lines 553).

• Moreover, please indicate what configuration is used (as PF-KPFM is used, the DC should be applied on sample). This aspect is important to discuss the type of charge (electron/hole) in KPFM.

We do not understand this comment. Indeed, our equation is identical to that of Melitz: we have only used other notations written $|q|$ instead of e to remove any ambiguity about the sign of $|q|$ since sometime q is used for the charge of the electron (negative) or the

elementary charge (positive). And in our setup the the potential is applied to the sample, while the tip is grounded. We have clarified these points in the text.

Moreover we have added in section 2 of the SI, the results obtained with KPFM over a reference sample made of alternating stripes of aluminum, silicon and gold.

3. Spatial resolution for topography and KPFM measurement

[29] KPFM resolution and tip

As protein dimension is small (8-12nm), please justify the fact to performed measurement with large curvature radius tip (PtIr coated tip) instead for low curvature radius one (doped Si for example). This has a strong impact on result accuracy and performance.

For recording the KPFM image, we used a conductive tip coated with PtIr (SCM-PITv2 from Bruker). This tip has a nominal radius of 25 nm and our AFM-KPFM images were recorded with the double-pass PeakForce Tapping KPFM-AM mode. This allows obtaining the topography and CPD map in exactly the same conditions.
Nevertheless we have added AFM topography images of AfFtn-AA recorded with a sharper silicon tip with a nominal radius of 2 nm (ScanAsyst-Air from Bruker). They offer a much better resolution but they are not conductive and do not allow recording KPFM images. These better resolved images were added in our text in Fig 1-d and in the Supporting Information in Fig. S4.

[30] AFM tip convolution

Concerning surface topography measurement on isolated ferritin particles. The authors argue that ferritin lateral width is constant whatever the Fe content (lines 180 pages 4). However, surface topography profiles (figure 2c) show clearly an increasing of the FWHM from 30nm (600Fe) to 60nm (4800Fe). Moreover, this width increasing is depicted on summary scheme (Figure 6d). Please discuss and clarify this point.

To record the KPFM images, we used a SCM-Pitv2 tip with a ≈ **25 nm radius**. The observed lateral width increase is due to the tip convolution with the ferritin particles. The height difference between ferritins creates the observed difference of lateral widths. To support our assumption, we performed lateral width measurements on (Fe4800) and (Fe2400) ferritins on silica surfaces using a ≈ **2 nm radius tip** (ScanAsyst-Air). The results show smaller lateral widths for both ferritin (≈ 19 nm) and a lateral width variation of 2 nm. This variation is coherent with the lateral width variation (+1.4 nm) calculated from ferritin perimeters (see Table 1 in Supp. Information). Therefore, the lateral expansion of ferritin particles upon adsorption on silica surfaces can be observed by using a very sharp tip, but with which we could not perform surface potential measurements. We propose a new figure in the Supplementary Data (Figure S4) showing the AFM images and height profiles from which results are described here. Moreover, even if we cannot observe with KFPM measurement that ferritin expands upon adsorption, it is more likely to happen, as we explain on the Figure 6-d.

[31] clarify Si, SiO2

Concerning surface topography measurement on monolayer, Figure S6 demonstrates that films are not continuous and these results permit to extract surface coverage. Please discuss the impact of poor lateral resolution of surface topography, on surface coverage determination.

We have added several AFM images recorded with a sharper tip (SCAN-Asyst-air, tip radius 2 nm) and prove that the counting was possible. But we agree that with the KFPM tip (SCM-Pit-

v2, radius 25 nm) the evaluation of the surface coverage is more difficult. We have included the error bars in Table 1 to take into account this limitation.

- [32] Concerning CDP measurement,

the contrast could occur from material difference and/or trapped charges. Pages (lines 214-216), the negative CDP is attributed to to charge distribution. Please justify this assumption. Indeed, this negative CDP could be attributed by proteins themselves instead of charge.

As explained in our text the negative value for the CPD is not due to a material difference, since we compared to iron nanoparticles of approximatively the same size (or slightly bigger) and the CPD values are still greater for Ftn that for the iron nanoparticles (details in Fig. 3e and the related text).
Moreover, the CPD values increase with the iron loading (addition of positively charges Fe^{2+} and Fe^{3+}) and we show with QCM that it only changes the internal structure inside the ferritin. It means that the CPD values truly expresses internal charge redistribution, that we measure with the change of the dipole moment.

- [33] The part dedicated to CDP measurement on isolated ferritin nanoparticle, is less convincing. Indeed, as error bars have the same order of magnitude as CDP variation with Fe loading, the trend is not convincing. Moreover, the lateral resolution is impacted by parasitic capacitance and topography lateral resolution (as measurements are performed in lift mode). The evolution in figure 3e could be related to Ferritin FWHM evolution (and related decreasing of the silica contribution to parasitic capacitance) on CDP. Please discuss this part.

We agree that KPFM does not have the sufficient resolution to come up with absolute values of CPD changes for isolated ferritin in Figure 3. This is the reason why we also measure the case of monolayers in Figure 4. However we trust that the combination of Fig 3 and Fig 4, that show the same trend are strong enough to support our conclusion that the dipole moment effectively undergoes an increase when the iron content increases.

- [34] Quite the same effect could explain for ferritin layer (figure 4).

As the ferritin layer is not continuous (mainly for low Fe loading), the ferritin CDP could be impact by substrate CDP. Please discuss this.

In the case of the monolayers of Ftn of Fig 4, the LSH for the KPFM was set at 20 nm and tip radius was 25 nm. therefore the tip record a surface potential which is globally average over 50-60 nm. We agree that our measure is an averaged over the Ftn and non-covered substrate. This “reduced” value of CPD is taken into account by the Helmhotz formula that we use to compute the dipole moment. We are confident that our values of dipole moments are correct.

- [35] On figure 4d,

the CDP profiles present a large transition area (around 1µm) at Si/Ferritin interface. Moreover, for 4800Fe a negative peak is observed (variation of 25mV compare to ferritin). Please discuss these.

We agree that the CPD profile over the edge of the Fe4800 monolayer exhibits a negative peak close the edge. We have notice that there were a trend of accumulation of the ferritin near the edge of the drop. Therefore, we have taken the CPD value (-110 mV in Fig 4c) a bit away from the edge.

4. [36] the question of measuring KPFM in air, vs. adsorption was in liquid

All measurements preformed by KPFM are done on dry surface instead of ferritin adhesion occurred in liquid media. In liquid, ions could have an impact on electrostatic interaction and ferritin dipolar moment is impact by the media (liquid VS dry air). Please discuss the validity of the transposition of results obtained on dry media to phenomenon occurring in liquid one.

Thanks for your comment. Indeed we have completed our results and discussion, in particular in making clear the role of the Na^{+} cations. See our answers to comments comments [2], [10], [16] and [23] above.

5. Minor remarks

• [37] Page 3:
please justify the fact that a ferritin monolayer is obtained. On figure 1c, white dots present height of around 18nm which could correspond to a ferritin nanoparticle on the top of layer. Please discuss this.

As explained above, we have modified Figure 1c with another AFM image recorded with a sharper tip. We have very limited occurrences of ferritin aggregates, although they are not zero. We consider this phenomenon as negligible.

• [38] QCM and LSR measurements are performed in dynamic mode.
Please justify this choice. Indeed, adhesion under shear stress flow undergoes different mechanisms than static condition. Please indicate the flow and related pressure.

Thanks for your comment. We have performed complementary QCM experiments in a stop-flow configurations as shown in Section 10 of the SI. These new results show in this condition, ferritin adsorb only if Na+ cations are present in the buffer solution.

• [39] **(Luis)** Figure 5,
please used the same parameter for figures and caption (K, F…)

Name of the parameters were corrected in the caption and in the text

Dear reviewers,

Thanks for your careful evaluation of our manuscript "**Unveiling the Immobilization Mechanism of Negatively Charged Protein Particles on Silica through Direct Measurement of the Electric Dipole"** authored by Lechaptois *et al*.

Following various comments of the six reviewers who have evaluated our manuscript, we believe we have strengthened our results. Following this second round of revision, we have only slightly amended our manuscript (text highlighted in blue) but we respond in details to the questions of the reviewers below. Finally, we would like to stress the three main findings of our article, and we are certain they will make an impact in the community of biophysics and biochemistry:

(1) Our study is the first experimental evidence of the adsorption of individual proteins on negative surfaces even if these proteins are negatively charged. We provide an explanation of this counter-intuitive observation by evidencing the mediation of the sodium counterion.
(2) This is also the first time KPFM is used to measure the electrostatics of proteins surfaces: we were able to measure the electric dipole of the adsorbed species in both configurations: on a single protein and on monolayers of protein. We have even provided values for the electric dipoles, that confirm theoretical prediction by other authors (theoreticians).
(3) Finally, we have measured the various consequences of loading more and more iron into our model protein: it leads to the morphologic distortion and to the non-monotonic variation of the electric dipole. These morphologic changes were interpreted thanks of QCM-D experiments.

Since the 1st submission of our manuscript in Oct 2024, we think that our results have grown in strength and we also believe that they bring a sound input to the question of the mechanism of protein adsorption. We also hope they will be helpful for theoreticians who need to design a proper model of adsorbed proteins.

The authors

REVIEWER COMMENTS (received on Jan 5th, 2026)

Reviewer #2 (Remarks to the Author):

The majority of my concerns have been addressed. The manuscript is much improved with the updated figures and expansion of discussions. It is easier to read now compared with previous version. The work is close to the level of recommendation of publication.

Our answer.
We thank you for your time in evaluating our manuscript and your positive comments.

Reviewer #4 (Remarks to the Author):

First of all, I want to thank the authors to addressed all remarks with relevant information. I just want to come back on remarks [28] concerning the sign of the CDP related to Melitz et al paper. I apologize if my remark was not clearly explained.
I agree with the definition of the CPD: VCPD= (WFsample-WFtip)/|q|. However, I do not agree with the relation between VDC and VCPD. According to Metliz et al., a DC bias applied on sample implies,

to null the electrostatic force, that VDC = - VCPD (equation 2.8).
This point is extremely important as the CDP sign impacts charges sign and consequently all the discussion in the paper. In this case, two options are identified: (i) the CDP discuss in all the paper correspond to – VDC obtained in KPFM measurement and all the discussion is OK or (ii) the CDP correspond to VDC obtained by KPFM and consequently the sign is false and the discussion is impacted (probed charges are no more negative but positive) and the paper need to be rewritten. Please check this issue.
Consequently, I consider that the paper can't be published in the present form.

Our answer.
We have taken very seriously your question regarding the sign of the DC bias applied in our KPFM setup. Indeed, this sign is critical for the subsequent discussion.
I have questioned the AFM manufacturer as well as several colleagues (in France and abroad) and I can insure that in our experiments, **the DC bias is applied to the tip**. We performed KPFM experiments with a Bruker Multimode 8, in the so-called PF-KPFM-AM mode. Therefore, we have $V_{DC} = V_{CPD}$ as stated in Equation 1 in our manuscript. I acknowledge that we were not extremely clear on this point. Nevertheless, in our study we constantly checked that the sign was correct, using the data acquired on the reference sample (See our figure S2 in the Supporting Information).

With this, we confirm the validity of the sign of the dipole measured in our manuscript, as well as our conclusions regarding the pivotal role of the sodium cations in the adhesion mechanism of the ferritin proteins.

Finally, we want to thank you for your very careful reading of our manuscript. Although your questions pushed us in revisiting many aspects of the manuscript, we think they truly helped us in improving our study.

Reviewer #5 (Remarks to the Author):

Reviewer #5 expressed his comments in the attached file (Ferritin.pdf), that we are reproducing below but without the figure.

I was asked to only answer the question of whether the third reviewer's comments had been addressed adequately. I am afraid that that is not the case. The reviewer was not very clear, though. The citations provided by the authors, which are supposed to answer the reviewer's objections, deal with films. But what the authors have deposited are not films. These are particles. That is to be expected, but it is also evident in the data. The Df-plots bend downwards, as is common for particulate adsorbates. The AFM micrographs also show particles.

[figure of adsorbed particles, with rocking motion capability]

The problem is sketched above. Particles do not undergo a shear deformation. They rather undergo a rocking-type motion. Also, the liquid undergoes a tortuous flow. That is difficult to model, in the first place. An attempt is formulated in Ref. 1. Ref. 1 deals with bacteria, but the equivalent circuit applies to smaller adsorbed objects, as well. Importantly, soft particles may flatten out at the bottom more than stiff particles. The bending stiffness then *increases*. The bending stiffness critically depends on the contact radius. The particle shown on the right-hand side in the plot above appears to be stiffer to the QCM than the particle on the left. That may or may not be so. Possibly, the softer particles have the lower D-f-ratio, but there is not guarantee. That is a difficult problem. I would not know how to analyze data such as the ones taken by the authors with confidence.

The statement „The protein layer is more viscoelastic at low loading and more rigid at high loading" is unfounded.
Actually, the attachment across Na^+ ions might affect the contact stiffness as seen with the QCM but again, the QCM data is not easily interpreted. Data from multiple overtones might have helped. Repeats with means and standard deviations might have helped.
I am not sure whether the QCM data are needed to support the main message of the work. I understand that the work is mostly concerned with the dipole and the attachment.

Our answer.
We thank you for your time in evaluating the questions raised by Reviewer #3 related to the physical interpretation of our QCM-D data, specifically regarding the dominance of particle rocking. While we agree that isolated particles can undergo a rocking-type motion, this mechanism is not a dominant factor in our specific experimental configuration. Our QCM-D data shows a frequency shift (Δf) of approximately 60 Hz at saturation, which indicates a dense monolayer coverage where lateral interactions between protein shells significantly restrict the freedom of individual particles to rock. As established in the literature, when a dense protein layer is formed, the dissipation shifts are primarily attributed to energy losses within the protein adlayer, specifically dynamic conformational changes and hydration, rather than interfacial slip or rocking. (See Höök, Rodahl, Brzezinski, Kasemo Langmuir 1998, 14, 4, 729–734, lxxtw>33hsm2svk354254653pe=;4<59y)

Furthermore, our study measures relative changes between ferritin variants that share an identical external protein shell and are adsorbed onto the same functional surface. Since the particle-surface attachment geometry and contact radius are constant across all samples, any dissipation driven purely by rocking or contact stiffness should remain similar. Instead, we observed a significant 35% difference in the *D*−*F* slope (*K*) between the low-iron (Fe600) and high-iron (Fe4800) variants. This systematic variation, despite identical attachment conditions, provides strong evidence that the dissipation changes are driven by the variable we altered: the internal iron core structure and its distinct influence on the protein's layer overall rigidity and hydration.

Finally, regarding the characterization of the layer as "viscoelastic," You mentioned that the statement regarding the protein layer being "more viscoelastic" was unfounded. To rigorously quantify this, we utilized Delta_D vs. Delta_F plots to calculate the slope K. (See [1] Höök et al. Proc. Joint Meeting of the European Frequency and Time Forum and the IEEE International Frequency Control Symposium, Besancon, France, 1999, pp. 966-972 vol.2, https://doi.org/10.1109/FREQ.1999.841467 and [2] Mazouzi et al, ACS Sens. 2022, 7, 6, 1657–1665, lxxtw>33hsm2svk354254653egwwirwsw25g46:9<)

Our data perfectly aligns with this model: the high-iron ferritin (Fe4800) exhibits a low K (rigid), while the low-iron ferritin (Fe600) exhibits a high K (viscoelastic). Furthermore, this interpretation is consistent with the large discrepancy between our acoustic mass (QCM-D) and optical mass (LSPR), which confirms that the softer layer is significantly more hydrated.
Therefore, while we acknowledge your point regarding rocking motion in general contexts, the specific comparative data and slope analysis in this study strongly support our conclusion regarding the viscoelastic properties of the ferritin layer.

Reviewer #6 (Remarks to the Author):

Task: This work already went thorough one round of peer review with us and the authors have revised the study according to reviewers' comments. Reviewer 1 is unfortunately unable to review.

We therefore ask if you could assess authors' responses to Reviewer 1, and let us know if they are sufficiently addressed. We do not require a full, new report, but an assessment of responses to Reviewer 1 only.

I believe the authors have largely addressed the questions raised by Reviewer #1; however, I am raising concerns regarding the significance of the study and the clarity of the central message the authors are trying to communicate.
The manuscript prepared by Lechaptois et al. investigated ferritin adsorption on silica and framed this phenomenon as a form of like-charge attraction mediated by cationic "electrostatic bridges." However, prior work by Krishnan et al. specifically demonstrated like-charge attraction as a solvent-mediated, long-range effect between negatively charged particles in water, and further showed that the addition of salt (e.g., NaCl) weakens the attraction due to screening instead of strengthening it. Rather, the study done by the authors demonstrates clearly that the observed adsorption is a counterion mediated effect, which is already well established. And it is not entirely clear how the measured dipole moment plays a role in the adsorption. While the experimental approach has been carefully re-examined and the KPFM measurements are technically interesting, the manuscript does not convincingly demonstrate a new mechanism of like-charge attraction beyond established counterion-mediated adsorption. Moreover, it remains unclear how the measured dipole moment contributes mechanistically to the adsorption process, as the dipole appears more likely to be a consequence of ion-mediated adsorption rather than a driving force.
In summary, I believe the significance of the manuscript lies more in the characterization of the adsorbed state of ferritin than in advancing the fundamental understanding of interactions between like-charged particles.

Our answer.
Here, we show that the findings published by *Krishnan and al* [our Ref 18] obtained on large radius (R = 4.8 µm), inert particles, can be extended to nanoscale proteins. We show that the positive counterions Na^+ are essential in the attachment of the ferritin proteins (see our Fig 2f). Our results clarifies and counterdict the calculations made by *Kubiak-Ossowska et al*. who performed molecular dynamics calculations, and concluded that adsorption was enabled by the positive arginine residue of BSA [Ref 54]. In that sense our results are solving a pending scientific question, of why negative proteins readily adsorbs on negatively charged surfaces. Moreover, this is the first time KPFM is used for bio-interface and our data give access to measurement of the dipole of single proteins. We could even calculate the values of the dipole moment of the adsorbed ferritins (see our Table 1). We anticipate that our results will be very helpful for theoreticians who need to design a proper model of adsorbed proteins.

We also stress that here, the dipole moment is not the origin of the adsorption. However, our ability to measure the dipole provides the local electrostatic configuration of proteins adsorbed on silica as depicted in figures 3c and 6b.

Regarding your remark on the screening of the attraction with high salt concentration, we have not explored these effects. Indeed, with KPFM we measure the resulting adsorbed configuration after the solvent is removed. Our Fig. 2f shows that the sodium cations are needed. The values for Na+ concentration are very different in Krishnan's study (15 µM to 1000 µM in their Fig 1b, [our Ref 18]) and in our study (10 mM to 400mM). Indeed, the sizes of the particles are very different, and therefore the specific surfaces are different in the two systems.

Finally, your comments has encouraged us to better stress the main findings of our study (see the introduction of this letter)

Specific comments following Reviewer #1's questions:

[1] Have the authors attempted measurements at different pH values? Figure 1 naturally raises question about how the adsorption state and the measured dipole moment would change with pH. While I understand the authors might prefer to focus on physiological conditions, control experiments at around pH 2 (both ferritin and silica being positively charged) and pH 4 (ferritin being positive while silica being negative) would aid to the robustness of the CPD measurements following Reviewer #1's request.

[5] DLVO theory describes colloidal stability and interaction potentials of particles in solution. It is therefore unclear how this study is directly relevant or transferable to the dry-state measurements performed by KPFM.

[6] One concerning point for me is the use of variable deposition times and the drying process of the buffer solution. How do the authors actively control the final salt concentration since it can increase substantially in the drying ("blowing away") process, such that ferritin is simply being "salted out"? Also, it would be helpful to clarify what is expected during the transient drying stage in terms of the state of protein adsorption and the dipole moment. How representative is the KPFM result measured at the air-silica interface compared to water-silica interface in solution?

[7] While the experimental work is careful and technically interesting, I do agree with Reviewer #1 that the central message of the manuscript is not yet sufficiently clear.

[1] Since we submitted the present results in Oct 2024, we have carried out a very comprehensive study with the AFM and we have specifically explored the dependance of the adsorption of ferritins with the pH value. This will be the topic of a next paper which is about to be submitted. We clearly distinguish different adsorption packing densities when pH changes from 2 to 8.

[5] DLVO, applies to the adsorption of the protein that takes place in solution. DLVO theory is important during deposition. We are not trying to transfer the DLVO theory into the dry state. And in our case, we measure the configuration of the deposited protein on the surface after removal of the surrounding liquid. We measured the final electrostatic arrangement with the KPFM. And the value and the direction of the dipole moments lead us to the understand that the Na^{+} cation was still underneath the ferritin.

[6] Ftn is not salted out. We performed control experiments with only sodium chloride and we saw no traces of Na+ on the silica surface (no remaining charges) as explained in the SI. We cannot do KPFM during the evaporation. KPFM is not possible in solution with our setup.

[7] This study is the 1st attempt to measure the adsorption of single protein. It should be a starting point for those who are doing calculation of the stability of proteins. Since our first submission : nobody did this kind of study.

Third set of comments received from the reviewers on March 10th, 2026.

Reviewer #4 (Remarks to the Author):

The reviewer wants to thank these authors to address all remarks and improve the manuscript. I think that manuscript could be published in the present form.

The authors thank the reviewer for supporting the publication of the manuscript.

Reviewer #5 (Remarks to the Author):

I am not convinced that the layers are dense films

The QCM part is below that current state of the art in my view

The authors thank the reviewers for the comments. The manuscript had refrained from claiming that the layers were dense films. In our manuscript we used the terminology "monolayer" and not dense films. We have revised the manuscript for clarity and to ensure that any reference on dense films have been removed. We do not claim that we produced "dense monolayers" neither. However, these monolayers are saturated: no more ferritin protein adsorbs spontaneously. The ferritins interact with each other in some way to produce this saturated layer of proteins. It surely prevents the rocking motion pointed by Reviewer #5.

Moreover, Reviewer seems to be puzzled by our statement "The protein layer is more viscoelastic at low loading and more rigid at high loading" (his comment received on Jan 5th, 2026)
We infer that there is probably a misunderstanding, since here the "loading" corresponds to the iron loading inside the ferritin. This is not the loading of ferritin at the surface. We have added clarification in the corresponding paragraph (page 12 in our manuscript).

Finally, the QCMD was performed to provide information on the mechanical property of the layer when the ferritin cages were loaded with different amounts of iron. Keeping the objective in mind, the QCMD performed using standard protocol would suffice. Interestingly, the measurement showed correlation between the mechanical property to the loading amount; hence, the charge transport properties from KPFM. This is the first report on such observation.

Reviewer #6 (Remarks to the Author):

The authors have addressed my previous concerns to some extent. While the experimental methods are technically rigorous and novel, the biological mechanistic claims appear slightly overstated. The dipole measurements are performed in a dry, post-adsorption state, which limits the extent to which the results can be interpreted in the context of adsorption mechanisms in aqueous environments.

Overall, I do not believe the manuscript is ready for recommendation to Nature Communications.

*The authors thank the **Reviewer#1** and **Reviewer#6** who took over the reviewing work, for the critical comments. They have pushed us a lot in adding new data and new insights in our study: starting from the measurement of the electric dipole we have extended our discussion, and proved the key role played by the sodium cation in the immobilization mechanism of ferritin.*

We also thank them for acknowledging the technical rigor and novel methods. The biological mechanistic claims were carefully reworded to refrain from overstating them.

We keep in mind that the adsorption in aqueous environment was a key step and the protein cages remained adsorbed despite multiple rising steps. This observation suggests that the interactions between the cages and the surface had been formed. The interaction might be due the remaining water molecules around the adsorbed protein cages as suggested by [our REF 16, Krishnan et al. https://doi.org/10.1038/s41565-024-01621-5*] in a similar system. Even though the dipole measurement in aqueous condition was impossible due to the limitation of the KPFM technique, the measurements provide correlations between aqueous adsorption and post-adsorption measurement, hence insights on the dipole of the system. The report on the dipole measurement is the first in the field and the values we have obtained are in agreement with calculated values for similar systems (see our refs 51, 52).*

Indeed, the reviewer's comments provide motivation to further in-depth study on the impacts of the aqueous environment on the adsorption and the dipole measurement. The authors are currently being investigating these phenomena and the manuscript is currently in preparation.

The author' response to the comments received from the reviewers on May 22nd, 2026.

REVIEWER COMMENTS

Reviewer #5 (Remarks to the Author):

The authors have shortened the QCM part. That was a step into the right direction.
The paper can be published from my side

The authors thank the reviewer for supporting the publication of the manuscript.

Reviewer #6 (Remarks to the Author):

I am still not convinced that the dipole measurements obtained in the dry, post-adsorption state can be directly translated to interfacial behaviour in an aqueous environment. While the authors acknowledge this limitation in the rebuttal, it is not clearly stated or sufficiently discussed in the manuscript itself, which I find potentially misleading. In addition, the manuscript does not explain how the surface charge state of the silica substrate evolves when transitioning from the aqueous environment to the dry measurement conditions, further limiting the interpretation of the results.

Overall, I do not see substantial improvement in addressing this key concern or in openly discussing it in the manuscript, and I do not support publication of the manuscript in Nature Communications.

Our Response.

Our experiments were performed on immobilized ferritin layers in air after adsorption from solution. We believe that the resulting layer retains a signature of the adsorption process that occurred in the liquid phase. However, we acknowledge that the KPFM measurements themselves were carried out in the dry state, as KPFM cannot currently be performed under the corresponding liquid-phase conditions

Throughout the manuscript and our responses, we have carefully described the experimental conditions and explicitly stated that the final characterization was conducted in air on dried samples. We do not wish to draw conclusions that extend beyond the scope of our measurements, nor to speculate on experiments that cannot presently be performed.

To further clarify the scope of our claims, we have revised the title from:

Unveiling the Immobilization Mechanism of Negatively Charged Protein Particles on Silica through Direct Measurement of the Electric Dipole

to:

Direct Measurement of Protein Dipoles Reveals a New Mechanism for Like-Charge Adsorption on Silica.

This revised title places greater emphasis on the methodological advance of the work and refers to "a" mechanism rather than "the" mechanism, thereby avoiding any implication of a unique or exhaustive explanation. In addition, we have added explicit statements at two locations in the manuscript indicating that the dipole measurements were performed in the dry state. We believe that these revisions clearly communicate the experimental framework and prevent any possible misunderstanding regarding the scope of our conclusions.

I was asked to only answer the question of whether the third reviewer's comments had been addressed adequately. I am afraid that that is not the case. The reviewer was not very clear, though.

The citations provided by the authors, which are supposed to answer the reviewer's objections, deal with films. But what the authors have deposited are not films. These are particles. That is to be expected, but it is also evident in the data. The Df-plots bend downwards, as is common for particulate adsorbates. The AFM micrographs also show particles.

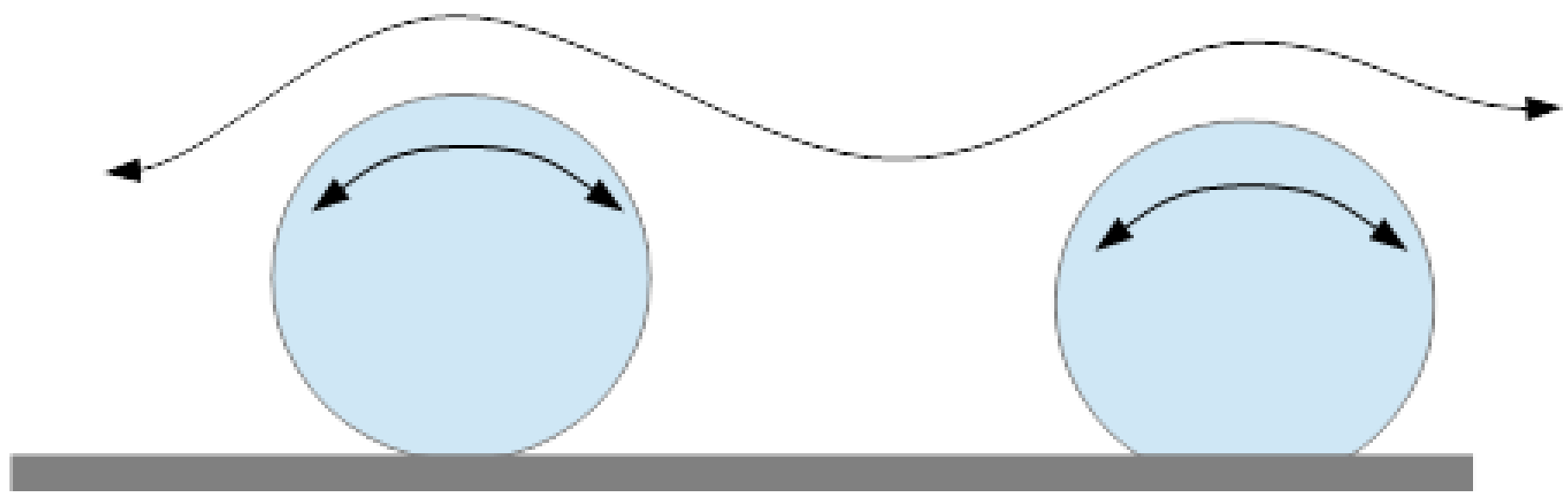

The problem is sketched above. Particles do not undergo a shear deformation. They rather undergo a rocking-type motion. Also, the liquid undergoes a tortuous flow. That is difficult to model, in the first place. An attempt is formulated in Ref. 1. Ref. 1 deals with bacteria, but the equivalent circuit applies to smaller adsorbed objects, as well. Importantly, soft particles may flatten out at the bottom more than stiff particles. The bending stiffness then *increases*. The bending stiffness critically depends on the contact radius. The particle shown on the right-hand side in the plot above appears to be stiffer to the QCM than the particle on the left. That may or may not be so. Possibly, the softer particles have the lower D-f-ratio, but there is not guarantee. That is a difficult problem. I would not know how to analyze data such as the ones taken by the authors with confidence.

The statement „The protein layer is more viscoelastic at low loading and more rigid at high loading" is unfounded.

Actually, the attachment across Na^+ ions might affect the contact stiffness as seen with the QCM but again, the QCM data is not easily interpreted. Data from multiple overtones might have helped. Repeats with means and standard deviations might have helped.

I am not sure whether the QCM data are needed to support the main message of the work. I understand that the work is mostly concerned with the dipole and the attachment.

---

[1] Tarnapolsky, A., und V. Freger. „Modeling QCM-D Response to Deposition and Attachment of Microparticles and Living Cells". *Analytical Chemistry* 90, Nr. 23 (2018): 13960–68. https://doi.org/10.1021/acs.analchem.8b03411.
